# Expanding the chemical diversity of spirooxindoles via alkylative pyridine dearomatization

**DOI:** 10.3762/bjoc.8.111

**Published:** 2012-07-02

**Authors:** Chunhui Dai, Bo Liang, Corey R J Stephenson

**Affiliations:** 1 Department of Chemistry and Center for Chemical Methodology and Library Development (CMLD-BU), Boston University, 590 Commonwealth Avenue, Boston, Massachusetts 02215, USA

**Keywords:** chemical diversity, 1,3-dicarbonyl compounds, Diels–Alder reaction, molecular diversity, pyridine dearomatization, spirooxindole

## Abstract

A mild and practical synthesis of spirooxindole [1,3]oxazino derivatives from *N*-substituted isatins and 1,3-dicarbonyl compounds with pyridine derivatives is reported. The reactions provided good to excellent yields. Further exploration of the molecular diversity of these compounds is demonstrated through Diels–Alder reactions.

## Introduction

**Scheme 1:**
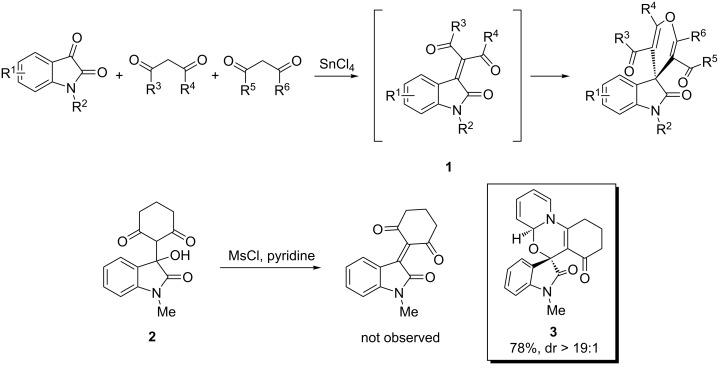
Unexpected alkylative pyridine dearomatization during our previous work on the synthesis of spirooxindole pyranochromenediones.

The spirooxindole is a common structural motif found in a variety of complex alkaloids [1]. Many compounds that possess a spirooxindole moiety exhibit significant biological activity, thus exemplifying their role in drug development [2–8]. Moreover, the challenging molecular architecture of spirooxindoles is appealing to chemists because it evokes novel synthetic strategies that address configurational demands and provides platforms for further reaction development [9–11]. To our knowledge, most studies of these types of molecules focus on spirooxindoles bearing a pyrrolidine ring at the 3-position of the oxindole core, while few reports expand to formulate the syntheses of other spiro rings. As part of our ongoing reaction-screening objective [12–13], we previously reported a Lewis acid catalyzed, three-component synthesis of spirooxindole pyranochromenedione derivatives using isatin and two 1,3-dicarbonyl compounds (Scheme 1) [14]. Mechanistically, we believed this reaction to proceed through an intermediate isatylidene **1** [15–17]. As a means to support our mechanistic hypothesis, we attempted to prepare and isolate the isatylidene; however, our attempts were unsuccessful. Interestingly, treatment of **2** with methanesulfonyl chloride (MsCl) in pyridine provided the dearomatized alkylation product **3** in 78% yield. In the context of developing novel and practical methods for the preparation of diverse heterocyclic compounds, herein, we report our extended investigation on the efficient synthesis of spirooxindole [1,3]oxazino derivatives by means of alkylative pyridine dearomatization [18–21].

## Results and Discussion

### Reactions of *N*-substituted isatins and 1,3-dicarbonyl compounds in pyridine

**Figure 1:**
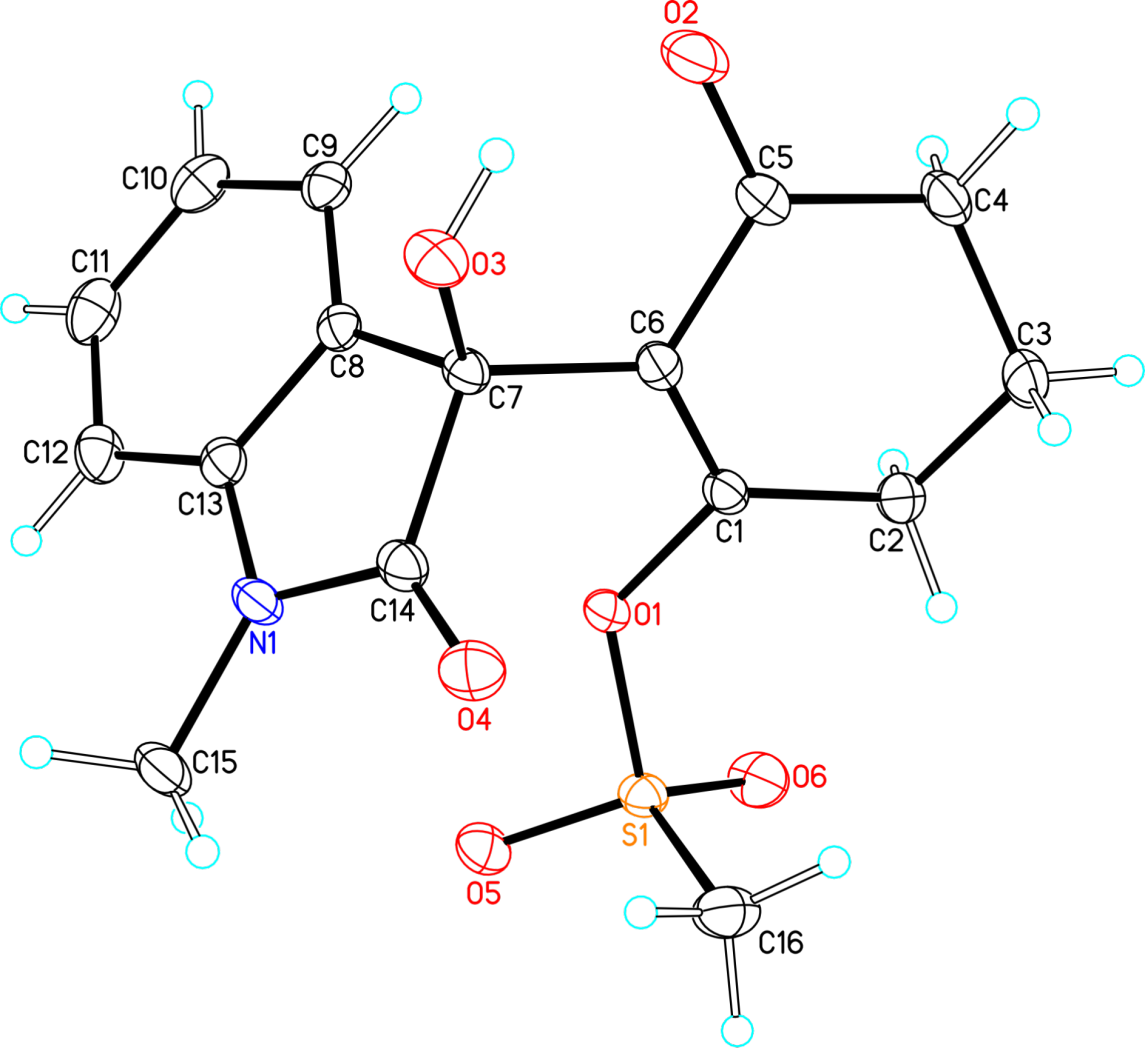
X-ray crystal structure of compound **6b**.

Based on preliminary results, we commenced with the reaction of *N*-substituted isatins **4** and 1,3-dicarbonyl compounds **5** in pyridine. After addition of the reagents, the mixture was allowed to react at room temperature for 2 h to ensure initial coupling, whereupon methanesulfonyl chloride was added slowly over a 1 h period at 0 °C and another 2 h at the same temperature to trap the vinylogous acid as vinyl mesylates **6**. Various *N*-substituted isatins and 1,3-dicarbonyl compounds were then explored and the results are presented in Table 1. Beginning with isatin and 1,3-cyclohexanedione (**5a**) as coupling partners, we isolated a relatively poor yield of product **6a** (Table 1, entry 1). We speculated that the free indole nitrogen was inhibiting the reaction, thus we switched to *N*-substituted isatins and found that the reaction improved to provide moderate to high yields (Table 1, entries 2–10). Subtle substitution effects were observed when the C(5)–H of isatin was replaced with various functionalities. Specifically, an electron-donating group at the 5-position, such as a methyl group, decreased the reactivity and only gave 57% yield (Table 1, entry 3). As a comparison, electron-withdrawing groups at the 5-position, such as chloro and nitro groups, increased the reactivity and provided products in higher yield (Table 1, entries 4–5). Other 1,3-dicarbonyl compounds were also investigated. 5,5-Dimethyl-1,3-cyclohexanedione (**5b**) worked well (Table 1, entry 6), in contrast to 5-phenyl-1,3-cyclohexanedione (**5c**), which gave a slightly lower yield (Table 1, entry 7). Interestingly, nonequivalent 1,3-dicarbonyl compound **5d** afforded single constitutional isomer (Table 1, entry 8), presumably due to the increased sterics and overall ring strain associated with substituents alpha to the vinylogous sulfonyl ester. *N*-Phenylisatin provided a similar yield to *N*-methylisatin (Table 1, entry 9). However, when *N*-acetylisatin was subjected to the reaction conditions, the reaction failed to provide the desired product and instead delivered compound **6j** exclusively in 74% yield (Table 1, entry 10). We predict that the formation of **6j** by dehydration is due to the electron deficiency of the oxindole ring and subsequent stability gained from the ene–trione moiety. The structure of compound **6b** was established by single-crystal X-ray analysis (Figure 1).

**Table 1:** Reaction of isatins **4** and 1,3-dicarbonyl compounds **5** in pyridine.

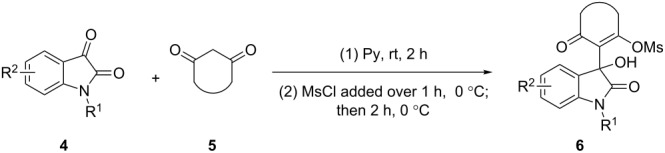					

entry^a^	R^1^	R^2^	**5**	product	yield (%)^b^

1	H	H	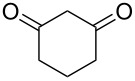 **5a**	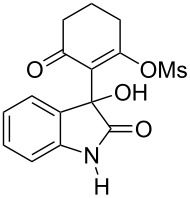 **6a**	31
2	Me	H	**5a**	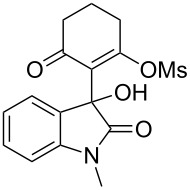 **6b**	80
3	Me	5-Me	**5a**	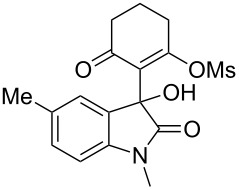 **6c**	57
4	Me	5-Cl	**5a**	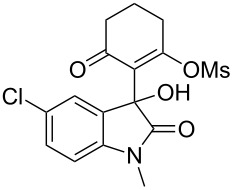 **6d**	84
5	Me	5-NO_2_	**5a**	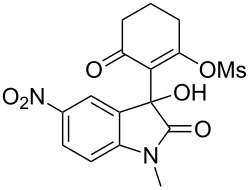 **6e**	89
6	Me	H	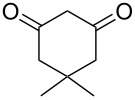 **5b**	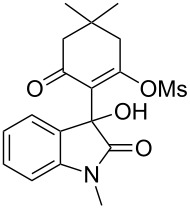 **6f**	87
7	Me	H	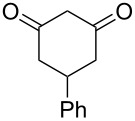 **5c**	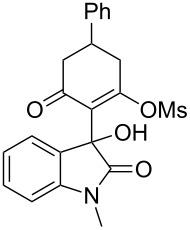 **6g**	61
8	Me	H	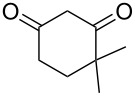 **5d**	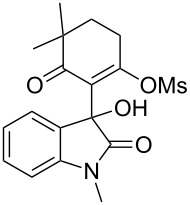 **6h**	71
9	Ph	H	**5a**	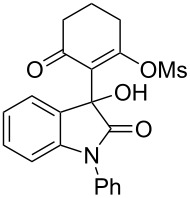 **6i**	73
10^c^	Ac	H	**5a**	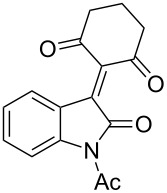 **6j**	74

^a^Reactions were carried out on a 10 mmol scale in pyridine (8.0 mL) with 1.0 equiv of isatins **4** and 1,3-dicarbonyl compounds **5** at room temperature for 2 h, followed by the addition of 1.5 equiv of MsCl at 0 °C over 1 h and another 2 h stirring at the same temperature. ^b^Isolated yield. ^c^The adduct **6j** was isolated as the only product.

**Figure 2:**
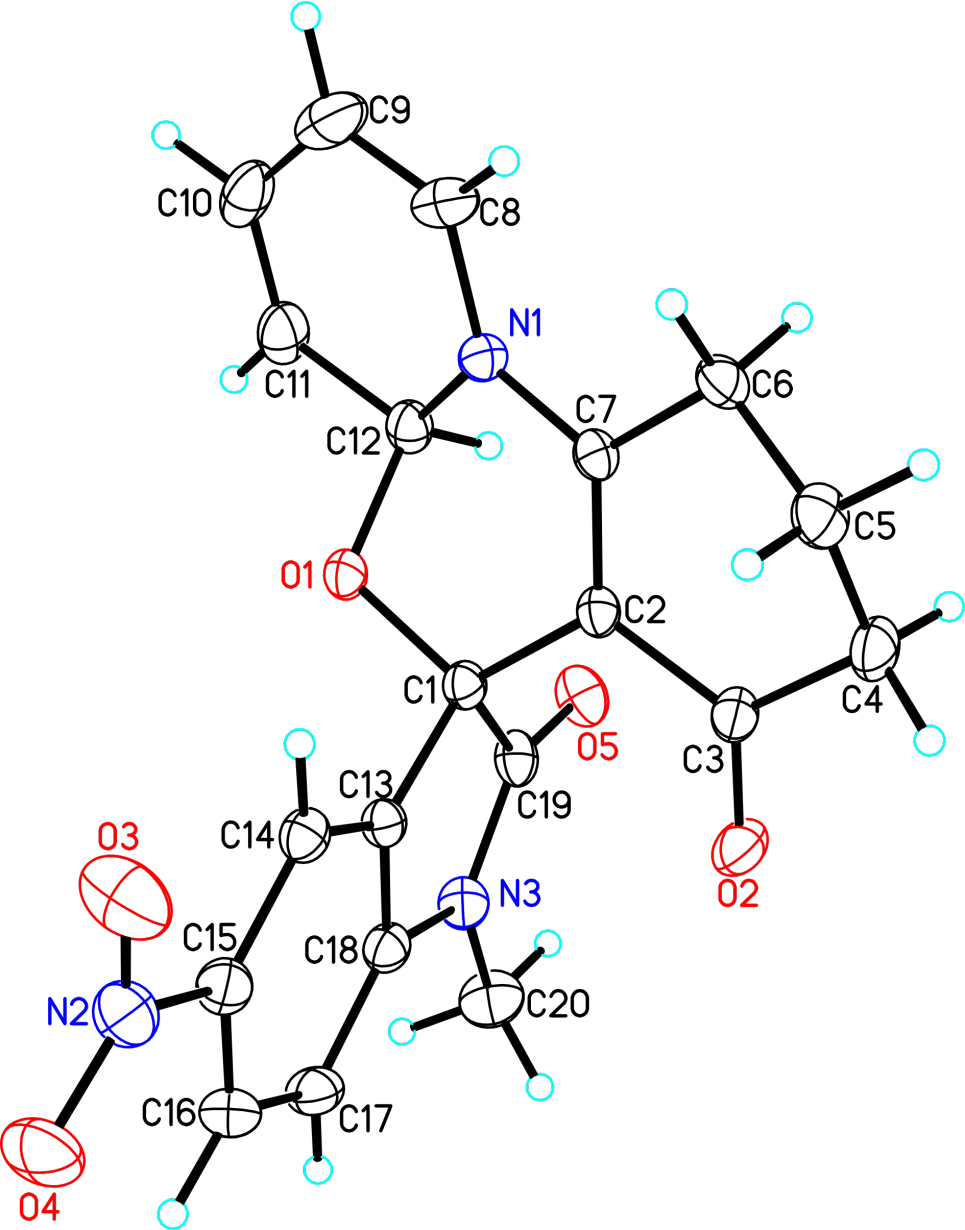
X-ray crystal structure of compound **3d**.

Having established a facile route to our desired vinylogous sulfonyl esters **6**, we next examined their reactivity towards alkylative pyridine dearomatization reactions (Table 2). During optimization studies, we discovered that the reaction performed best at 45 °C using pyridine as the solvent. The substituent groups on the isatin moiety did not have a great effect on the reactivity of compounds **6**. Generally, the reaction was complete in 12 h and provided inseparable diastereoisomers with good to excellent yields (Table 2, entries 1–5). 4-Picoline (**7b**) gave a lower yield after 12 h at 45 °C (Table 2, entry 6). 4-Methoxypyridine (**7c**) provided the desired product **3g** in 78% isolated yield in only 6 h at room temperature (Table 2, entry 7). Isoquinoline (**7d**) and 4-acetylpyridine (**7e**) also underwent the reaction to provide the desired products in moderate yields (Table 2, entries 8–9), but required an elevated temperature (60 °C) and a longer reaction time (18 h). On the basis of the results in Table 2, we speculate that the unexpected formation of compound **3** in Scheme 1 was due to the evaporation of solvent after the reaction at elevated temperature (45 °C), providing an opportunity for the vinylogous sulfonate ester, generated during the reaction, to react with pyridine. Finally, the structure of compound **3d** was established by single-crystal X-ray analysis (Figure 2).

**Table 2:** Reactions of vinylogous sulfonyl esters **6** with pyridine derivatives **7**.

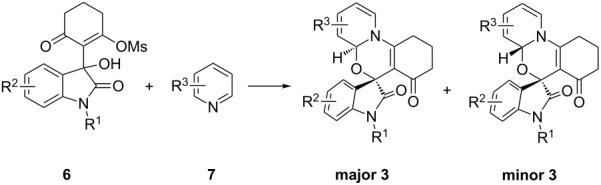							

entry^a^	**6**	**7**	T (°C)	h	product **3**	dr^b^	yield (%)^c^

1	**6b**	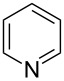 **7a**	45	12	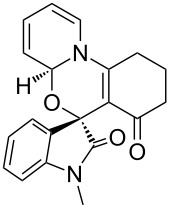 **3a**	5:1	90
2	**6c**	**7a**	45	12	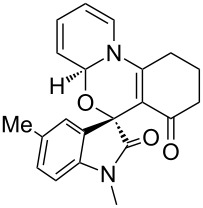 **3b**	9:1	83
3	**6d**	**7a**	45	12	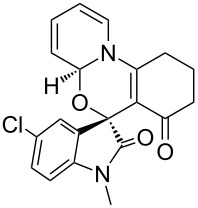 **3c**	7:1	90
4	**6e**	**7a**	45	12	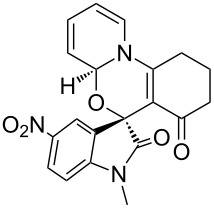 **3d**	5:1	91
5	**6i**	**7a**	45	12	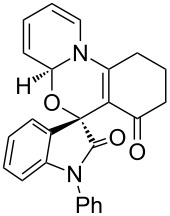 **3e**	5:1	74
6	**6b**	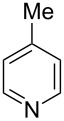 **7b**	45	12	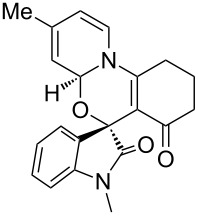 **3f**	8:1	48
7	**6b**	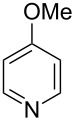 **7c**	23	6	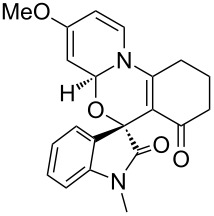 **3g**	3:1	78
8	**6b**	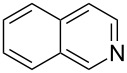 **7d**	60	18	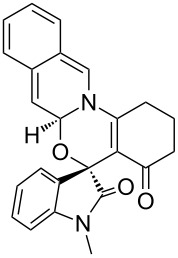 **3h**	2:1	65
9	**6d**	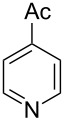 **7e**	60	18	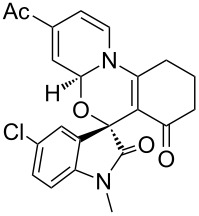 **3i**	8:1	67

^a^Reactions were conducted with vinylogous sulfonyl esters **6** (1.0 mmol) and pyridine derivatives **7** (1.0 mL) at 23–60 °C. Reaction time varied from 6–18 h. ^b^Determined by ^1^H NMR integration. ^c^Isolated yield.

**Scheme 2:**
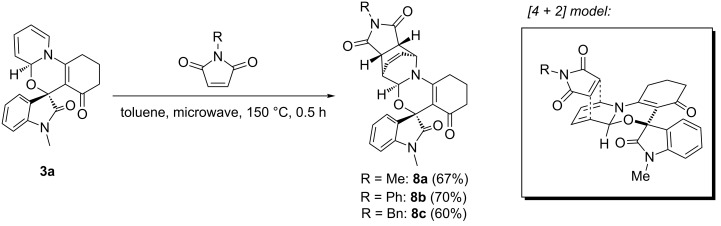
Application of spiro [1,3]oxazino compound **3a** in D–A reactions.

**Figure 3:**
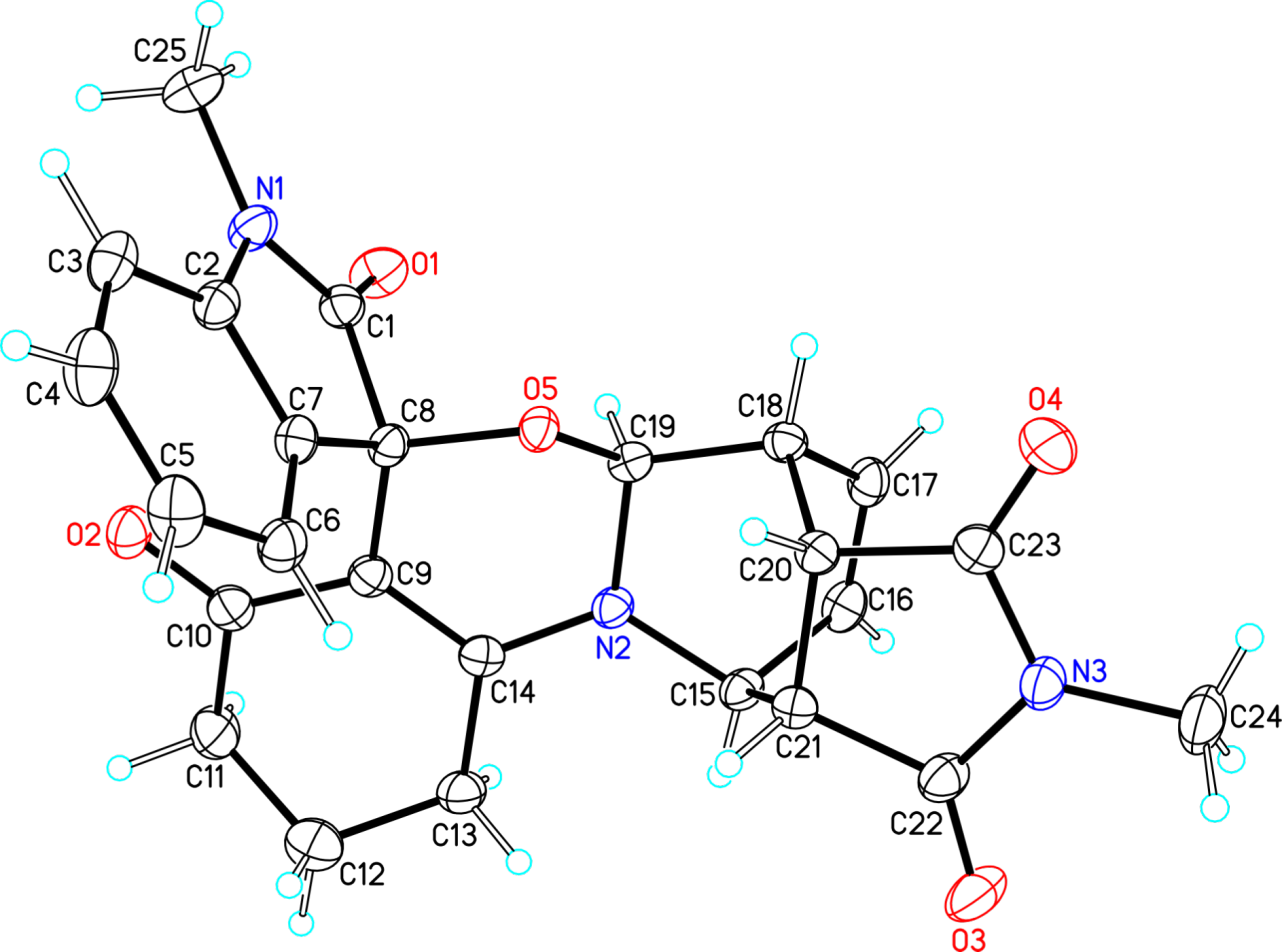
X-ray crystal structure of compound **8a**.

Further plans to expand the molecular diversity of these compounds utilizing available functionalities are currently underway. As an illustrative example, spiro [1,3]oxazino compounds having a diene moiety within their molecular framework are susceptible to Diels–Alder (D–A) reactions [22]. Scheme 2 highlights three examples in which compound **3a** was exposed to *N*-substituted maleimides in toluene at 150 °C under microwave irradiation for 0.5 h, and D–A products **8a**–**c** were isolated in moderate yields. Finally, the structure of compound **8a** was established by single-crystal X-ray analysis (Figure 3).

## Conclusion

In summary, we developed a practical and efficient method to synthesize spirooxindole derivatives with a [1,3]oxazine fused-ring system. The reaction conditions are very mild and tolerant of functional groups, providing moderate to high yields. The application and versatility of these spirooxindole derivatives to quickly access complex molecules is further demonstrated in good yielding D–A reactions.

## Supporting Information

File 1 Full experimental details and analytical data and crystallographic information.
