# Supplementary material for: Expanding the chemical diversity of spirooxindoles via alkylative pyridine dearomatization
Source: Beilstein J Org Chem. 2012 Jul 2;8:986–93. doi: 10.3762/bjoc.8.111 (PMC3458780; doi:10.3762/bjoc.8.111)

Supporting Information

for

Expanding the chemical diversity of spirooxindoles via alkylative pyridine dearomatization

Chunhui Dai, Bo Liang and Corey R. J. Stephenson*

Address: Department of Chemistry and Center for Chemical Methodology and Library Development (CMLD-BU), Boston University, 590 Commonwealth Avenue, Boston, Massachusetts 02215, USA

Email: Corey R. J. Stephenson* - crjsteph@bu.edu

* Corresponding author

Full experimental details and analytical data and crystallographic information

Table of contents

I. **General information and procedure...……………………………………………S2**

II. **Experimental procedures and compound characterization…………..…....S3–S22**

III. **X-ray crystallographic data..……………………………………………..S23–S30**

IV. **^{1}H NMR and ^{13}C NMR spectra….…………………………………...…..S31–S74**

V. **gHMBC spectra of compound 6h………..…………..………………..…S75–S76**

A. General information:

Chemicals were either used as received or purified according to the procedures outlined in *Purification of Common Laboratory Chemicals* [1]. Glassware was dried in a 170 °C oven or flame dried under vacuum and cooled under inert atmosphere before use. All reactions were performed using common dry, inert atmosphere techniques. Reactions were monitored by TLC and visualized by a dual short-wave/long-wave UV lamp and stained with an aqueous solution of potassium permanganate. Column flash chromatography was performed using 230–400 mesh silica gel. Yields refer to chromatographically and spectroscopically pure compounds, unless otherwise noted.

^{1}H and ^{13}C NMR spectra were recorded using an internal deuterium lock on Varian Mercury 300, Varian Unity Plus 400, or a Varian 500 spectrometers. All signals are reported in ppm with the internal reference of 7.26 ppm or 77.0 ppm for chloroform, 2.50 ppm or 39.5 ppm for DMSO as standard. Data are presented as follows: multiplicity (s = singlet, d = doublet, t = triplet, q = quartet, quint = quintet, m = multiplet, br = broad, dd = doublet of doublet, dt = doublet of triplet), coupling constant (J/Hz) and integration. Infrared spectra were recorded on a Nicolet Nexus 670 FT-IR with ATR spectraphotometer. Absorptions are given in wavenumbers (cm^{-1}). High-resolution mass spectra were obtained on a Waters Q-Tof API-US with ESI high resolution mass spectrometer. HPLC analysis was performed on an Agilent 1100 series HPLC System with a diode array detector. Microwave reactions were carried out using a CEM Explorer/Discover system equipped with a dynamic cooling valve.

B. Reagents and solvents

All reagents were purchased from Aldrich and TCI and were used directly without further purification. Substituted *N*-methyl isatins were synthesized according to a literature procedure [2].

Chemical names were generated using ChemDraw Ultra 10.0 (CambridgeSoft).

C. General Procedures

General procedure for reactions of *N*-substituted isatins and 1,3-dicarbonyl compounds in pyridine (Table 1)

A flame-dried 10 mL three-neck round-bottom flask with rubber septums and magnetic stir bar was charged with *N*-substituted isatin **4** (10 mmol, 1.0 equiv), 1,3-dicarbonyl compound **5** (10 mmol, 1.0 equiv) and anhydrous pyridine (8.0 mL). The reaction mixture was stirred at room temperature under an argon atmosphere for 2 h, and then it was cooled to 0 °C. MsCl (15 mmol, 1.5 equiv**)** was added using a syringe pump over 1 h, and the reaction was stirred for another 2 h at 0 °C. The mixture was quenched with 30 mL ice water and the solution was acidified with 1M hydrochloric acid. The acidified solution was then extracted with CH_2Cl_2 (3 × 100 mL) and the combined organic layers were washed with H_2O (100 mL), brine, dried (Na_2SO_4) and concentrated under reduced pressure. The residue was purified by chromatography on SiO_2 (1:1, hexane/EtOAc) to afford the desired product **6** as a solid.

(*Note: Compounds **6** are generally not very stable and slowly decompose at room temperature.*)

General procedure for the synthesis of spirooxindole compounds (Table 2)

A flame-dried 10 mL round-bottom flask with a rubber septum and magnetic stir bar was charged with substrate **6** (1.0 mmol, 1.0 equiv) and pyridine derivative **7** (1.0 mL). The reaction was conducted under argon atmosphere at different temperatures according to the reactivity of substrate **7**. After the reaction was finished, the mixture was cooled to room temperature and the solvent was removed (methods varied for **7**, details are included for each substrate). The residue was purified by chromatography on SiO_2 (60:1, CH_2Cl_2/MeOH) to afford the desired product **3** as a solid.

General Procedure for Diels-Alder Reactions (Scheme 2)

Compound **3** (0.15 mmol, 1.0 equiv) and *N*-substituted maleimide (0.18 mol, 1.2 equiv) were dissolved in 0.3 mL toluene. The reaction was conducted under microwave irradiation at 150 °C for 0.5 h. After the reaction mixture was cooled to room temperature, the solvent was removed under vacuum and the residue was purified by chromatography on SiO_2 (30:1, CH_2Cl_2/MeOH) to afford the product **8** as a solid.

Compound Characterization

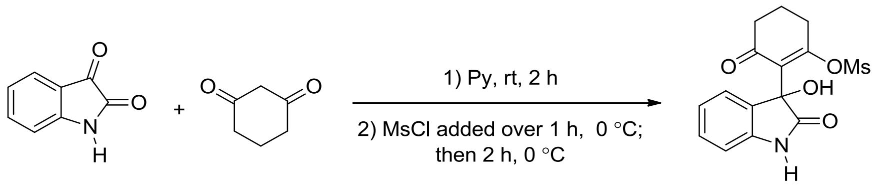

6a

2-(3-Hydroxy-2-oxoindolin-3-yl)-3-oxocyclohex-1-en-1-yl methanesulfonate (6a_Table 1, entry 1): According to the general procedure, isatin (1.47 g, 10.0 mmol) was treated with 1,3-cyclohexanedione (1.12 g, 10.0 mmol) in pyridine (8.0 mL), then MsCl (1.16 mL, 15.0 mmol) was added slowly. The reaction mixture was subjected to the workup protocol and purified by chromatography on SiO_2 using hexane/ethyl acetate (1:2) as the eluent to afford 1.04 g (31% yield) of the title compound as a yellow solid.

R_f (EtOAc/hexanes 6:1): 0.34;

IR (neat): 3258 (br), 3022, 2957, 1709, 1619, 1472, 1360, 1193, 1140, 756 cm^{-1};

^{1}H NMR (DMSO-d_6, 400 MHz): δ 10.22 (s, 1H), 7.21–7.15 (m, 2H), 6.87 (t, J = 7.6 Hz, 1H), 6.78 (d, J = 7.6 Hz, 1H), 6.29 (br s, 1H), 3.42 (s, 3H), 2.95–2.76 (m, 2H), 2.39–2.22 (m, 2H), 1.94–1.87 (m, 2H);

^{13}C NMR (DMSO-d_6, 100 MHz): δ 198.0, 176.9, 165.9, 142.7, 132.2, 129.3, 127.4, 124.0, 121.2, 109.5, 75.5, (one carbon overlaps with DMSO), 36.7, 30.2, 20.1;

MS (ESI) m/z calcd for $C_{15}H_{15}NO_6SNa$ ($[M + Na]^+$) 360.0518, found 360.0526.

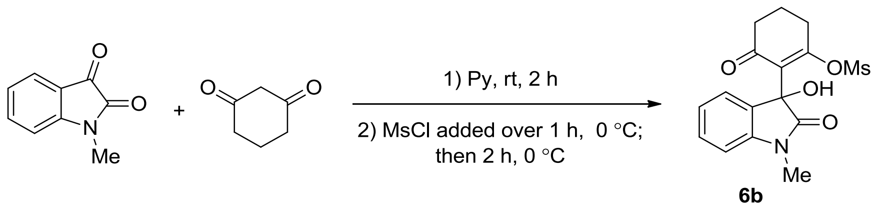

6b

2-(3-Hydroxy-1-methyl-2-oxoindolin-3-yl)-3-oxocyclohex-1-en-1-yl methanesulfonate (6b_Table 1, entry 2): According to the general procedure, *N*-methylisatin (1.61 g, 10.0 mmol) was treated with 1,3-cyclohexanedione (1.12 g, 10.0 mmol) in pyridine (8.0 mL), then MsCl (1.16 mL, 15.0 mmol) was added slowly. The reaction mixture was subjected to the workup

protocol and purified by chromatography on SiO_2 using hexane/ethyl acetate (1:1) as the eluent to afford 2.81 g (80% yield) of the title compound as a colorless solid.

R_f (EtOAc/hexanes 6:1): 0.39;

IR (neat): 3329 (br), 3015, 2930, 1717, 1611, 1472, 1351, 1145, 966, 754 cm^{-1};

^{1}H NMR (DMSO-d_6, 400 MHz): δ 7.31–7.25 (m, 2H), 6.98–6.94 (m, 2H), 6.38 (br s, 1H), 3.40 (s, 3H), 3.10 (s, 3H), 2.95–2.76 (m, 2H), 2.39–2.22 (m, 2H), 1.94–1.87 (m, 2H);

^{13}C NMR (DMSO-d_6, 125 MHz): δ 198.1, 175.3, 165.9, 144.0, 131.4, 129.4, 127.3, 123.7, 122.0, 108.4, 75.1, (one carbon is overlapping with DMSO), 36.7, 30.0, 26.0, 20.0;

MS (ESI) *m/z* calcd for $C_{16}H_{17}NO_6SNa$ ($[M + Na]^+$) 374.0674, found 374.0677.

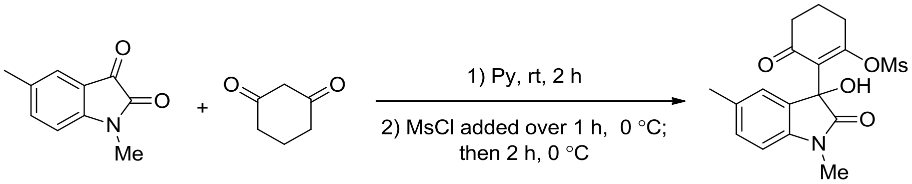

6c

2-(3-Hydroxy-1,5-dimethyl-2-oxoindolin-3-yl)-3-oxocyclohex-1-en-1-yl methanesulfonate (6c_Table 1, entry 3): According to the general procedure, 1,5-dimethylisatin (1.75 g, 10.0 mmol) was treated with 1,3-cyclohexanedione (1.12 g, 10.0 mmol) in pyridine (8.0 mL), then MsCl (1.16 mL, 15.0 mmol) was added slowly. The reaction mixture was subjected to the workup protocol and purified by chromatography on SiO_2 using hexane/ethyl acetate (1:1) as the eluent to afford 2.08 g (57% yield) of the title compound as a light yellow solid.

R_f (EtOAc/hexanes 6:1): 0.37;

IR (neat): 3367 (br), 3018, 2931, 1716, 1621, 1501, 1351, 1141, 957, 750 cm^{-1};

^{1}H NMR (DMSO-d_6, 400 MHz): δ 7.08 (d, J = 7.6 Hz, 1H), 7.07 (s, 1H), 6.85 (d, J = 7.6 Hz, 1H), 6.34 (br s, 1H), 3.40 (s, 3H), 3.08 (s, 3H), 2.94–2.76 (m, 2H), 2.39–2.21 (m, 2H), 2.23 (s, 3H), 1.94–1.87 (m, 2H);

^{13}C NMR (DMSO-d_6, 100 MHz): δ 198.2, 175.3, 165.8, 141.7, 131.4, 130.8, 129.5, 127.2, 124.4, 108.2, 75.2, (one carbon overlaps with DMSO), 36.7, 29.9, 26.0, 20.7, 20.0;

MS (ESI) *m/z* calcd for $C_{17}H_{19}NO_6SNa$ ($[M + Na]^+$) 388.0831, found 388.0822.

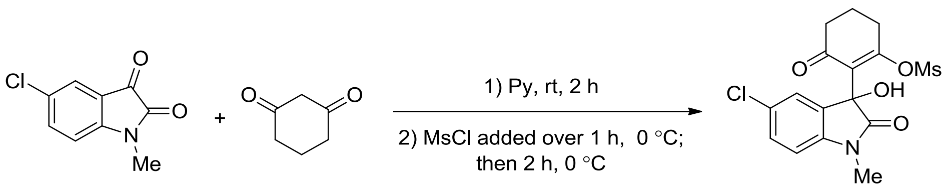

2-(5-Chloro-3-hydroxy-1-methyl-2-oxoindolin-3-yl)-3-oxocyclohex-1-en-1-yl methanesulfonate (6d_Table 1, entry 4): According to the general procedure, 1-methyl-5-chloroisatin (1.95 g, 10.0 mmol) was treated with 1,3-cyclohexanedione (1.12 g, 10.0 mmol) in pyridine (8.0 mL), then MsCl (1.16 mL, 15.0 mmol) was added slowly. The reaction mixture was subjected to the workup protocol and purified by chromatography on SiO_2 using hexane/ethyl acetate (1:1) as the eluent to afford 3.25 g (84% yield) of the title compound as a light yellow solid.

R_f (EtOAc/hexanes 6:1): 0.41;

IR (neat): 3163 (br), 2952, 1713, 1611, 1492, 1375, 1147, 974, 783 cm^{-1};

^1H NMR (DMSO-d_6, 400 MHz): δ 7.35 (dd, J = 8.4, 2.4 Hz, 1H), 7.27 (d, J = 2.4 Hz, 1H), 7.01 (d, J = 8.4 Hz, 1H), 6.55 (br s, 1H), 3.10 (s, 3H), 3.01 (s, 3H), 2.94–2.78 (m, 2H), 2.36–2.26 (m, 2H), 1.95–1.87 (m, 2H);

^{13}C NMR (DMSO-d_6, 100 MHz): δ 198.1, 175.1, 166.4, 143.0, 133.2, 129.2, 125.9, 123.8, 110.0, 75.1, 39.4, 36.6, 30.2, 26.2, 20.0;

MS (ESI) m/z calcd for $C_{16}H_{16}ClNO_6SNa$ ($[M + Na]^+$) 408.0285, found 408.0277.

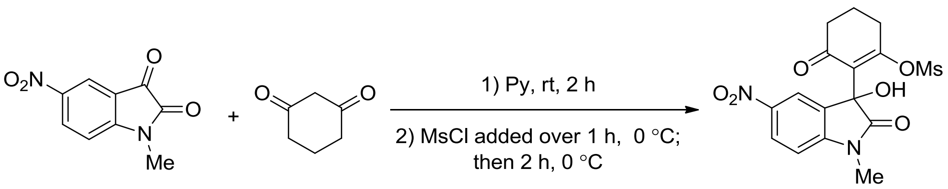

2-(3-Hydroxy-1-methyl-5-nitro-2-oxoindolin-3-yl)-3-oxocyclohex-1-en-1-yl methanesulfonate (6e_Table 2, entry 5): According to the general procedure, 5-nitro-1-methylisatin (2.06 g, 10.0 mmol) was treated with 1,3-cyclohexanedione (1.12 g, 10.0 mmol) in pyridine (8.0 mL), then MsCl (1.16 mL, 15.0 mmol) was added slowly. The reaction mixture was subjected to the workup protocol and purified by chromatography on SiO_2 using

hexane/ethyl acetate (1:1) as the eluent to afford 3.52 g (89% yield) of the title compound as a yellow solid.

R_f (EtOAc/hexanes 6:1): 0.44;

IR (neat): 3385 (br), 3019, 2939, 1740, 1614, 1521, 1335, 1293, 758 cm^{-1};

^{1}H NMR (DMSO-d_6, 500 MHz): δ 8.28 (dd, J = 8.5, 2.5 Hz, 1H), 8.12 (d, J = 2.5 Hz, 1H), 7.23 (d, J = 8.5 Hz, 1H), 6.76 (s, 1H), 3.49 (s, 3H), 3.21 (s, 3H), 2.95–2.83 (m, 2H), 2.37–2.26 (m, 2H), 1.97–1.87 (m, 2H);

^{13}C NMR (DMSO-d_6, 125 MHz): δ 198.2, 175.9, 167.0, 150.1, 142.3, 132.1, 126.8, 119.3, 108.7, 74.6, 39.6, 36.4, 30.3, 26.5, 20.0;

MS (ESI) m/z calcd for $C_{16}H_{16}N_2O_8SNa$ ([M + Na]$^+$) 419.0525, found 419.0534.

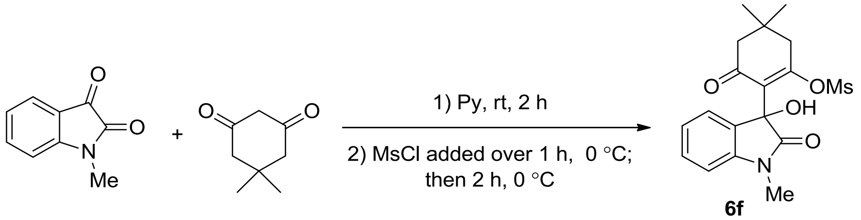

2-(3-Hydroxy-1-methyl-2-oxoindolin-3-yl)-5,5-dimethyl-3-oxocyclohex-1-en-1-yl methanesulfonate (6f_Table 1, entry 6): According to the general procedure, *N*-methylisatin (1.61 g, 10.0 mmol) was treated with 5,5-dimethyl-1,3-cyclohexanedione (1.40 g, 10.0 mmol) in pyridine (8.0 mL), then MsCl (1.16 mL, 15.0 mmol) was added slowly. The reaction mixture was subjected to the workup protocol and purified by chromatography on SiO_2 using hexane/ethyl acetate (1:1) as the eluent to afford 3.31 g (87% yield) of the title compound as a colorless solid.

R_f (EtOAc/hexanes 6:1): 0.57;

IR (neat): 3372 (br), 3019, 2961, 1721, 1611, 1472, 1352, 1183, 967, 754 cm^{-1};

^{1}H NMR (DMSO-d_6, 400 MHz): δ 7.30–7.23 (m, 2H), 6.98–6.94 (m, 2H), 6.41 (s, 1H), 3.43 (s, 3H), 3.10 (s, 3H), 2.76 (dd, J = 57.6, 18.0 Hz, 2H), 2.21 (dd, J = 47.2, 15.6 Hz, 2H), 1.03 (s, 3H), 0.95 (s, 3H);

^{13}C NMR (DMSO-d_6, 125 MHz): δ 198.0, 175.4, 163.9, 144.1, 131.5, 129.4, 126.4, 123.3, 121.9, 108.4, 75.3, 50.3, 43.7, 32.4, 27.2, 26.7, 26.0 (two carbons);

MS (ESI) m/z calcd for $C_{18}H_{21}NO_6SNa$ ([M + Na]$^+$) 402.0987, found 402.0995.

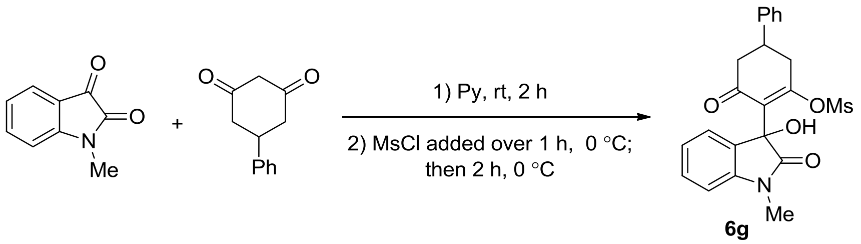

4-(3-Hydroxy-1-methyl-2-oxoindolin-3-yl)-5-oxo-1,2,5,6-tetrahydro-[1,1'-biphenyl]-3-yl methanesulfonate (6g_Table 1, entry 5): According to the general procedure, *N*-methylisatin (1.61 g, 10.0 mmol) was treated with 5-phenyl-1,3-cyclohexanedione (1.88 g, 10.0 mmol) in pyridine (8.0 mL), then MsCl (1.16 mL, 15.0 mmol) was added slowly. The reaction mixture was subjected to the workup protocol and purified by chromatography on SiO_2 using hexane/ethyl acetate (1:1) as the eluent to afford 2.62 g (61% yield) of the title compound as a colorless solid.

R_f (EtOAc/hexanes 6:1): 0.63;

IR (neat): 3350 (br), 3023, 2936, 1721, 1611, 1472, 1354, 1145, 966, 750 cm^{-1};

^1H NMR (CD_2Cl_2, 500 MHz): δ 7.38–7.34 (m, 4H), 7.31–7.24 (m, 3H), 7.10–7.06 (m, 1H), 6.88 (d, J = 8.0 Hz, 1H), 3.48–3.41 (m, 1H), 3.25–3.16 (m, 4H), 3.10–3.01 (m, 4H), 2.89–2.76 (m, 2H);

^{13}C NMR (DMSO-d_6, 100 MHz): δ 197.0, 175.5, 165.0, 144.2, 142.0, 131.1, 129.5, 128.6, 127.1, 123.9, 122.0, 108.4, 75.1, 43.3, (one carbon is overlapped with DMSO), 37.7, 37.3, 26.1;

MS (ESI) *m*/*z* calcd for $C_{22}H_{21}NO_6SNa$ ($[M + Na]^+$) 450.0987, found 450.0999.

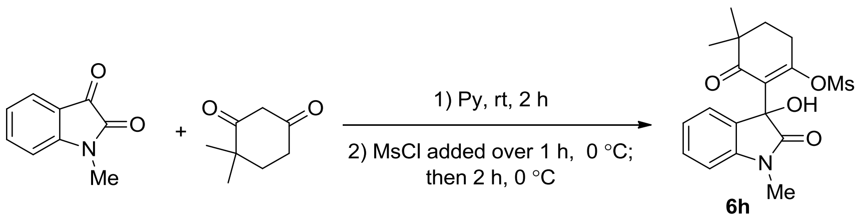

6h

2-(3-Hydroxy-1-methyl-2-oxoindolin-3-yl)-4,4-dimethyl-3-oxocyclohex-1-en-1-yl methanesulfonate (6h_Table 1, entry 8): According to the general procedure, *N*-methylisatin (1.61 g, 10.0 mmol) was treated with 4,4-dimethyl-1,3-cyclohexanedione (1.40 g, 10.0 mmol) in pyridine (8.0 mL), then MsCl (1.16 mL, 15.0 mmol) was added slowly. The reaction mixture was subjected to the workup protocol and purified by chromatography on SiO_2 using hexane/ethyl acetate (1:1) as the eluent to afford 2.68 g (71% yield) of the title compound as a colorless solid.

R_f (EtOAc/hexanes 6:1): 0.56;

IR (neat): 3367 (br), 3018, 2934, 1722, 1611, 1351, 1175, 750 cm^{-1};

^{1}H NMR ($CDCl_3$, 500 MHz): δ 7.33–7.26 (m, 2H), 7.03 (t, *J* = 7.5 Hz, 1H), 6.84 (d, *J* = 7.5 Hz, 1H), 3.24 (s, 3H), 3.01 (s, 3H), 2.89 (t, *J* = 6.3 Hz, 2H), 1.98–1.84 (m, 2H), 1.16 (s, 6H);

^{13}C NMR (DMSO-d_6, 100 MHz): δ 202.4, 175.5, 163.5, 144.1, 131.5, 129.5, 125.8, 123.4, 122.0, 108.5, 75.5, 40.3, 39.5, 32.8, 27.6, 26.0, 23.6, 23.3;

MS (ESI) *m*/*z* calcd for $C_{18}H_{21}NO_6SNa$ ($[M + Na]^+$) 402.0987, found 402.0989.

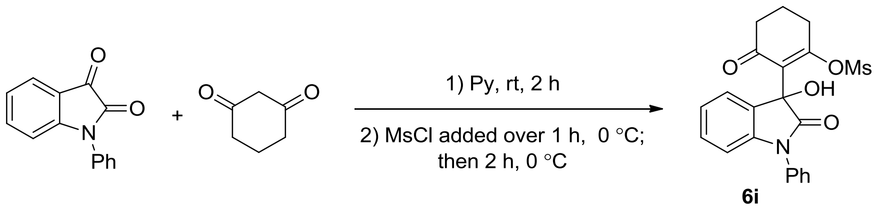

6i

2-(3-Hydroxy-1-methyl-2-oxoindolin-3-yl)-3-oxocyclohex-1-en-1-yl methanesulfonate (6i_Table 1, entry 9): According to the general procedure, *N*-phenylisatin (2.23 g, 10.0 mmol) was treated with 1,3-cyclohexanedione (1.12 g, 10.0 mmol) in pyridine (8.0 mL), then MsCl (1.16 mL, 15.0 mmol) was added slowly. The reaction mixture was subjected to the workup protocol and purified by chromatography on SiO_2 using hexane/ethyl acetate (1:1) as the eluent to afford 3.02 g (73% yield) of the title compound as a colorless solid.

R_f (EtOAc/hexanes 6:1): 0.66;

IR (neat): 3371 (br), 3019, 2936, 1729, 1610, 1500, 1359, 1148, 962, 752 cm^{-1};

^{1}H NMR ($CDCl_3$, 500 MHz): δ 7.56–7.51 (m, 4H), 7.44–7.40 (m, 2H), 7.26–7.23 (m, 1H), 7.08 (dt, J = 7.5, 1.0 Hz, 1H), 6.85 (d, J = 8.0 Hz, 1H), 3.02–2.94 (m, 5H), 2.28–2.53 (m, 2H), 2.14–2.09 (m, 2H);

^{13}C NMR ($CDCl_3$, 100 MHz): δ 200.2, 174.5, 165.9, 143.7, 134.1, 130.3, 129.9, 129.5, 128.1, 126.5, 124.4, 123.5, 109.8, 76.0, 39.5, 37.2, 29.9, 20.1

MS (ESI) m/z calcd for $C_{21}H_{19}NO_6SNa$ ($[M + Na]^+$) 436.0831, found 436.0827.

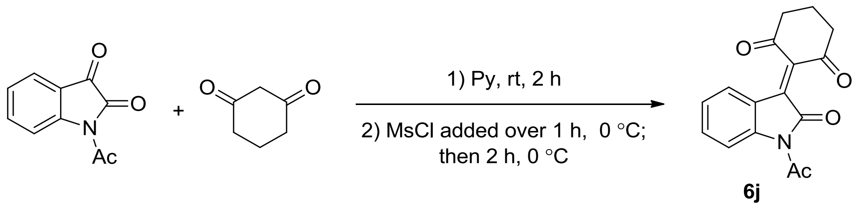

6j

2-(1-Acetyl-2-oxoindolin-3-ylidene)cyclohexane-1,3-dione (6j_Table 1, entry 10): According to the general procedure, *N*-acetylisatin (1.89 g, 10.0 mmol) was treated with 1,3-cyclohexanedione (1.12 g, 10.0 mmol) in pyridine (8.0 mL), then MsCl (1.16 mL, 15.0 mmol) was added slowly. The reaction mixture was subjected to the workup protocol and purified by chromatography on SiO_2 using hexane/ethyl acetate (1:1) as the eluent to afford 2.11 g (74% yield) of the title compound as a yellow solid.

R_f (EtOAc/hexanes 1:2): 0.33;

IR (neat): 3020, 2957, 1752, 1709, 1598, 1461, 1367, 1341, 1281, 1175, 768 cm^{-1};

^{1}H NMR ($CDCl_3$, 500 MHz): δ 8.28–8.24 (m, 2H), 7.47 (dt, J = 8.0, 1.5 Hz, 1H), 6.84 (t, J = 8.0 Hz, 1H), 2.98 (t, J = 7.0 Hz, 2H), 2.78 (t, J = 7.0 Hz, 2H), 2.66 (s, 3H), 2.11 (quint, J = 7.0 Hz, 2H);

^{13}C NMR ($CDCl_3$, 100 MHz): δ 199.9, 197.9, 170.0, 166.4, 146.3, 142.2, 133.7, 129.0, 126.4, 125.2, 120.0, 116.3, 42.2, 40.8, 26.6, 17.7;

MS (ESI) m/z calcd for $C_{16}H_{13}NO_4Na$ ($[M + Na]^+$) 306.0742, found 306.0751.

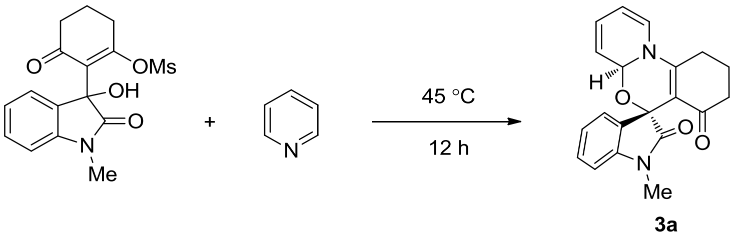

(4aS)-1'-Methyl-9,10-dihydro-4a*H*-spiro[benzo[d]pyrido[2,1-b][1,3]oxazine-6,3'-indoline]-2',7(8*H*)-dione (3a_Table 2, entry 1) According to the general procedure, compound **6b** (351 mg, 1.0 mmol) was dissolved in pyridine (1.0 mL) and heated at 45 °C for 12 h. The solvent was removed in vacuo and the residue was purified by chromatography on SiO_2 using CH_2Cl_2/MeOH (60:1) as the eluent to afford 302 mg (90% yield) of the title compound as a light yellow solid.

R_f (EtOAc/hexanes 6:1): 0.34;

IR (neat): 3011, 2953, 1716, 1642, 1560, 1403, 754 cm^{-1};

^1H NMR ($CDCl_3$, 500 MHz, 5:1): δ 7.28–7.24 (m, 2H), 7.03 (d, *J* = 7.0 Hz, 1H), 6.93 (t, *J* = 7.5 Hz, 1H), 6.82 (d, *J* = 7.5 Hz, 1H), 6.75–6.72 (m, 2H), 6.25–6.20 (m, 1H), 5.61–5.57 (m, 1H), 5.34 (t, *J* = 6.8 Hz, 1H), 3.12 (s, 3H), 2.98 (dt, *J* = 17.5, 5.0 Hz, 1H), 2.61–2.53 (m, 1H), 2.33–2.27 (m, 2H), 2.18–2.12 (m, 1H), 2.00–1.92 (m, 1H);

Diagnostic signals for the minor isomer: δ 7.33–7.29 (m, 0.2H), 6.98 (t, *J* = 7.5 Hz, 0.19H), 6.85 (d, *J* = 8.0 Hz, 0.19H), 6.66 (d, *J* = 8.0 Hz, 0.16H), 5.45–5.42 (m, 0.16H), 5.30 (t, *J* = 6.8 Hz, 0.18H), 2.92–2.85 (m, 0.19H);

^{13}C NMR ($CDCl_3$, 100 MHz): δ 193.4, 174.4, 155.2, 145.2, 129.6, 124.7, 123.1, 122.3, 122.1, 117.0, 115.0, 108.1, 101.2, 79.1, 35.7, 26.0, 25.0, 19.9;

Diagnostic signals for the minor isomer: δ 193.2, 174.7, 155.4, 143.6, 129.1, 123.5, 123.3, 121.8, 116.5, 113.1, 79.5, 26.4, 25.2, 20.8;

MS (ESI) *m/z* calcd for $C_{20}H_{19}N_2O_3$ ($[M + H]^+$) 335.1396, found 335.1395.

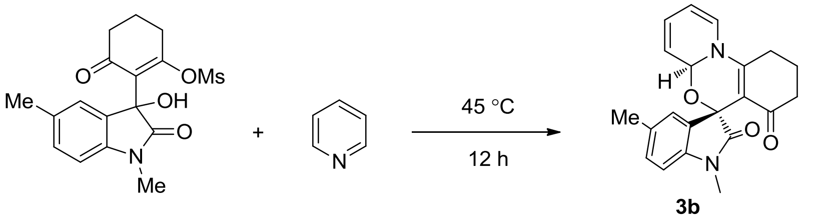

3b

(4aS)-1'-Methyl-9,10-dihydro-4a*H*-spiro[benzo[d]pyrido[2,1-b][1,3]oxazine-6,3'-indoline]-2',7(8*H*)-dione (3b_Table 2, entry 2): According to the general procedure, compound **6c** (365 mg, 1.0 mmol) was dissolved in pyridine (1.0 mL) and heated at 45 °C for 12 h. The solvent was removed in vacuo and the residue was purified by chromatography on SiO_2 using CH_2Cl_2/MeOH (60:1) as the eluent to afford 302 mg (83% yield) of the title compound as a colorless solid.

R_f (EtOAc/hexanes 6:1): 0.34;

IR (neat): 3011, 2945, 1711, 1641, 1558, 1400, 1348, 747 cm^{-1};

^{1}H NMR ($CDCl_3$, 400 MHz, 9:1): δ 7.05 (d, J = 8.0 Hz, 1H), 6.85 (s, 1H), 6.75–6.70 (m, 3H), 6.22 (dd, J = 10.0, 2.0 Hz, 1H), 5.60–5.56 (m, 1H), 5.33 (t, J = 6.8 Hz, 1H), 3.22 (s, 3H), 2.99 (dt, J = 17.2, 4.8 Hz, 1H), 2.61–2.53 (m, 1H), 2.34–2.28 (m, 2H), 2.23 (s, 3H), 2.18–2.12 (m, 1H), 2.01–1.94 (m, 1H);

Diagnostic signals for the minor isomer: δ 7.11 (d, J = 7.6 Hz, 0.12H), 6.66 (d, J = 7.6 Hz, 0.12H), 5.47–5.43 (m, 0.11H), 2.93–2.84 (m, 0.14H),

^{13}C NMR ($CDCl_3$, 125 MHz): δ 193.6, 174.5, 155.1, 143.0, 131.9, 130.0, 129.1, 124.7, 123.2, 123.1, 117.3, 115.3, 108.0, 101.4, 79.2, 35.9, 26.1, 25.2, 20.9, 20.1;

Diagnostic signals for the minor isomer: δ 193.4, 174.7, 155.3, 141.4, 131.2, 129.9, 124.9, 124.5, 123.6, 116.9, 113.4, 107.9, 101.3, 79.6, 36.0, 26.5, 25.4, 21.0;

MS (ESI) m/z calcd for $C_{21}H_{21}N_2O_3$ ($[M + H]^+$) 349.1552, found 349.1542.

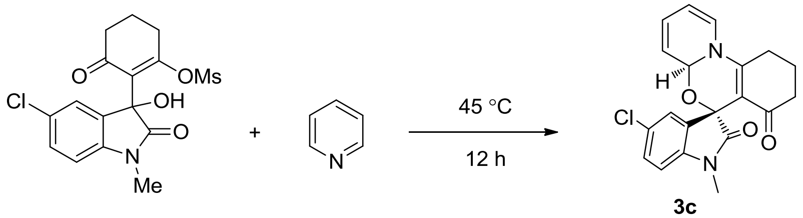

(3'R,4aS)-5'-Chloro-1'-methyl-9,10-dihydro-4a*H*-spiro[benzo[d]pyrido[2,1-b][1,3]oxazine-6,3'-indoline]-2',7(8*H*)-dione (3c_Table 2, entry 3): According to the general procedure, compound **6d** (386 mg, 1.0 mmol) was dissolved in pyridine (1.0 mL) and heated at 45 °C for 12 h. The solvent was removed in vacuo and the residue was purified by chromatography on SiO_2 using CH_2Cl_2/MeOH (60:1) as the eluent to afford 330 mg (90% yield) of the title compound as a light yellow solid.

R_f (EtOAc/hexanes 6:1): 0.41;

IR (neat): 3011, 2945, 1718, 1639, 1557, 1401, 1101, 747 cm^{-1};

^1H NMR ($CDCl_3$, 400 MHz, 7:1): δ 7.22 (dd, J = 6.2, 2.0 Hz, 1H), 7.00 (d, J = 2.0 Hz, 1H), 6.75–6.67 (m, 3H), 6.26–6.22 (m, 1H), 5.58 (dd, J = 10.0, 3.2 Hz, 1H), 5.35 (t, J = 6.8 Hz, 1H), 3.48 (s, 3H), 3.23 (s, 3H), 3.00 (dt, J = 17.2, 4.8 Hz, 1H), 2.62–2.53 (m, 1H), 2.34–2.28 (m, 2H), 2.19–2.13 (m, 1H), 2.03–1.97 (m, 1H);

Diagnostic signals for the minor isomer: δ 5.48–5.44 (m, 0.15H), 2.93–2.85 (m, 0.18H);

^{13}C NMR ($CDCl_3$, 100 MHz): δ 193.7, 174.2, 155.7, 144.0, 131.1, 129.5, 127.7, 125.0, 123.1, 122.8, 117.1, 114.7, 109.2, 101.7, 79.3, 35.8, 26.2, 25.2, 20.0;

Diagnostic signals for the minor isomer: δ 193.5, 174.5, 156.1, 142.4, 132.6, 129.4, 127.2, 125.2, 124.0, 123.5, 116.6, 112.7, 109.2, 101.6, 79.8, 35.9, 26.6, 25.4, 20.9;

MS (ESI) *m/z* calcd for $C_{20}H_{18}ClN_2O_3$ ($[M + H]^+$) 369.1006, found 369.1005.

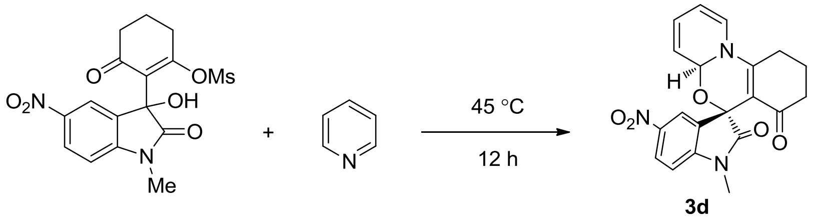

3d

(3'R,4aS)-1'-Methyl-5'-nitro-9,10-dihydro-4a*H*-spiro[benzo[d]pyrido[2,1-b][1,3]oxazine-6,3'-indoline]-2',7(8*H*)-dione (3d_Table 2, entry 4): According to the general procedure, compound **6e** (396 mg, 1.0 mmol) was dissolved in pyridine (1.0 mL) and heated at 45 °C for 12 h. The solvent was removed in vacuo and the residue was purified by chromatography on SiO_2 using CH_2Cl_2/MeOH (60:1) as the eluent to afford 343 mg (91% yield) of the title compound as a yellow solid.

R_f (EtOAc/hexanes 6:1): 0.44;

IR (neat): 3063, 2945, 1731, 1613, 1557, 1402, 1330, 1071, 911, 726 cm^{-1};

^1H NMR ($CDCl_3$, 500 MHz, 5:1): δ 8.25 (dd, *J* = 8.5, 2.5 Hz, 1H), 7.93 (d, *J* = 2.5 Hz, 1H), 6.90 (d, *J* = 8.5 Hz, 1H), 6.77 (d, *J* = 8.0 Hz, 1H), 6.65–6.64 (m, 1H), 6.29–6.25 (m, 1H), 5.60–5.56 (m, 1H), 5.42 (t, *J* = 6.8 Hz, 1H), 3.31 (s, 3H), 3.05 (dt, *J* = 17.0, 5.0 Hz, 1H), 2.65–2.58 (m, 1H), 2.36–2.30 (m, 2H), 2.22–2.15 (m, 1H), 2.06–1.98 (m, 1H);

Diagnostic signals for the minor isomer: δ 8.31 (dd, *J* = 8.5, 2.5 Hz, 0.13H), 8.17 (d, *J* = 2.0 Hz, 0.15H), 6.92 (d, *J* = 8.5 Hz, 0.15H), 6.70 (d, *J* = 7.5 Hz, 0.16H), 2.95–2.89 (m, 0.18H);

^{13}C NMR ($CDCl_3$, 100 MHz): δ 193.7, 174.5, 156.4, 151.1, 143.0, 130.5, 126.7, 125.1, 123.1, 118.2, 116.2, 114.0, 107.7, 101.8, 79.3, 35.5, 26.4, 25.0, 19.8;

Diagnostic signals for the minor isomer: δ 193.6, 175.0, 156.6, 149.3, 142.6, 131.4, 125.2, 123.3, 118.9, 116.3, 112.0, 107.6, 101.8, 79.9, 35.6, 26.8, 25.2, 20.6;

MS (ESI) *m*/*z* calcd for $C_{20}H_{18}N_3O_5$ ($[M + H]^+$) 380.1246, found 380.1253.

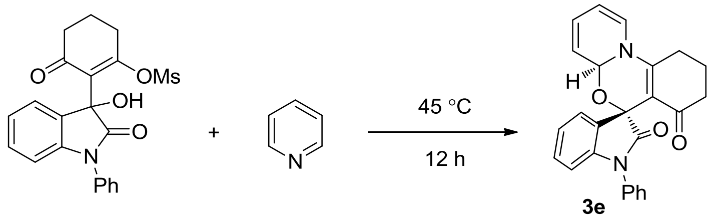

(3'R,4aS)-1'-Phenyl-9,10-dihydro-4a*H*-spiro[benzo[d]pyrido[2,1-b][1,3]oxazine-6,3'-indoline]-2',7(8*H*)-dione (3e_Table 2, entry 5): According to the general procedure, compound **6i** (413 mg, 1.0 mmol) was dissolved in pyridine (1.0 mL) and heated at 45 °C for 12 h. The solvent was removed in vacuo and the residue was purified by chromatography on SiO_2 using CH_2Cl_2/MeOH (60:1) as the eluent to afford 295 mg (74% yield) of the title compound as a colorless solid.

R_f (EtOAc/hexanes 6:1): 0.56;

IR (neat): 3061, 2951, 1722, 1610, 1558, 1401, 1303, 1199, 910, 729 cm^{-1};

^{1}H NMR ($CDCl_3$, 400 MHz, 8:1): δ 7.57–7.49 (m, 5H), 7.42–7.37 (m, 1H), 7.18 (dt, *J* = 7.6, 1.6 Hz, 1H), 7.12–7.09 (m, 1H), 6.98–6.94 (m, 1H), 6.78–6.73 (m, 3H), 5.67–5.63 (m, 1H), 5.39–5.35 (m, 1H), 3.02 (dt, *J* = 17.6, 4.8 Hz, 1H), 2.63–2.54 (m, 1H), 2.37–2.32 (m, 2H), 2.21–2.14 (m, 1H), 2.04–1.94 (m, 1H);

Diagnostic signals for the minor isomer: δ 7.34–7.32 (m, 0.15H), 6.69–6.67 (m, 0.12H), 5.53–5.49 (m, 0.13H), 5.33–5.30 (m, 0.13H), 2.93–2.85 (m, 0.19H);

^{13}C NMR ($CDCl_3$, 125 MHz): δ 193.8, 174.2, 155.3, 145.8, 134.7, 129.6, 129.5, 128.9, 127.9, 126.8, 124.8, 123.2, 122.9, 122.7, 117.4, 115.9, 109.6, 101.6, 79.5, 36.0, 25.2, 20.2;

Diagnostic signals for the minor isomer: δ 193.5, 174.3, 155.5, 144.3, 134.9, 131.0, 129.6, 127.0, 125.1, 123.9, 123.6, 123.3, 122.3, 116.9, 113.8, 109.5, 101.5, 79.7, 36.0, 25.5, 21.0;

MS (ESI) *m*/*z* calcd for $C_{25}H_{21}N_2O_3$ ($[M + H]^+$) 397.1552, found 397.1553.

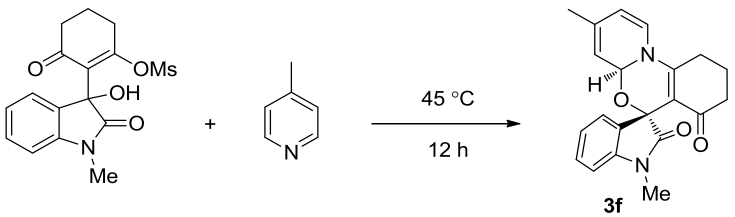

(3'R,4aS)-1',3-Dimethyl-9,10-dihydro-4a*H*-spiro[benzo[d]pyrido[2,1-b][1,3]oxazine-6,3'-indoline]-2',7(8*H*)-dione (3f_Table 2, entry 6): According to the general procedure, compound **6b** (351 mg, 1.0 mmol) was dissolved in 4-picoline (1.0 mL) and heated at 45 °C for 12 h. The solvent was removed in vacuo and the residue was purified by chromatography on SiO_2 using CH_2Cl_2/MeOH (60:1) as the eluent to afford 166 mg (48% yield) of the title compound as a colorless solid.

R_f (EtOAc/hexanes 6:1): 0.37;

IR (neat): 3001, 2941, 1715, 1632, 1564, 1402, 1306, 748 cm^{-1};

^{1}H NMR ($CDCl_3$, 500 MHz, 5:1): δ 7.26–7.22 (m, 1H), 7.02 (dd, *J* = 7.5, 1.0 Hz, 1H), 6.91 (dt, *J* = 7.5, 1.0 Hz, 1H), 6.81 (d, *J* = 7.5 Hz, 1H), 6.68 (d, *J* = 7.5 Hz, 1H), 6.64–6.62 (m, 1H), 5.34–5.32 (m, 1H), 5.20 (dd, *J* = 7.5, 2.0 Hz, 1H), 3.24 (s, 3H), 2.98 (dt, *J* = 17.5, 5.0 Hz, 1H), 2.59–2.52 (m, 1H), 2.33–2.26 (m, 2H), 2.17–2.12 (m, 1H), 1.98–1.94 (m, 1H), 1.84 (s, 3H);

Diagnostic signals for the minor isomer: δ 6.96 (t, *J* = 7.5 Hz, 0.2H), 6.84 (d, *J* = 8.0 Hz, 0.2H), 2.92–2.85 (m, 0.18H);

^{13}C NMR ($CDCl_3$, 100 MHz): δ 193.5, 174.5, 155.1, 145.2, 133.6, 129.5, 122.6, 122.3, 122.1, 115.0, 112.7, 108.1, 105.0, 79.4, 35.8, 26.0, 25.0, 20.8, 19.9;

Diagnostic signals for the minor isomer: δ 193.2, 174.7, 155.4, 143.6, 133.9, 131.1, 129.5, 123.3, 123.0, 121.8, 113.1, 112.2, 108.1, 104.8, 79.7, 35.8, 26.4, 25.3, 20.7;

MS (ESI) *m*/*z* calcd for $C_{21}H_{21}N_2O_3$ ($[M + H]^+$) 349.1552, found 349.1552.

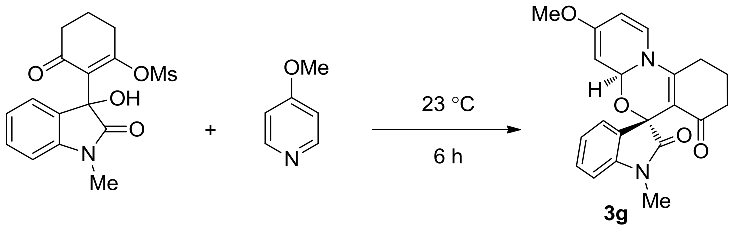

(4aS)-3-methoxy-1'-methyl-9,10-dihydro-4a*H*-spiro[benzo[d]pyrido[2,1-b][1,3]oxazine-6,3'-indoline]-2',7(8*H*)-dione (3g_Table 2, entry 7): According to the general procedure, compound **6b** (351 mg, 1.0 mmol) was dissolved in 4-methoxypyridine (1.0 mL) and stirred at 23 °C for 6 h. After the reaction was complete, 1.0 mL of cold MeOH was added to allow the product to precipitate out. Then the mixture was filtered and the solid was purified by chromatography on SiO_2 using CH_2Cl_2/MeOH (40:1) as the eluent to afford 284 mg (78% yield) of the title compound as a colorless solid.

R_f (EtOAc/hexanes 6:1): 0.31;

IR (neat): 2953, 1712, 1642, 1570, 1391, 1235, 1110, 911, 725 cm^{-1};

^{1}H NMR ($CDCl_3$, 500 MHz, 3:1): δ 7.31–7.25 (m, 1H), 7.13 (d, *J* = 7.5 Hz, 1H), 6.99–6.94 (m, 2H), 6.51 (d, *J* = 4.0 Hz, 1H), 5.22 (dd, *J* = 8.0, 2.5 Hz, 1H), 4.65 (t, *J* = 2.5 Hz, 1H), 3.52 (s, 3H), 3.19–3.12 (m, 1H), 3.11 (m, 3H), 2.61–2.53 (m, 1H), 2.26–1.98 (m, 3H), 1.85–1.80 (m, 1H); Diagnostic signals for the minor isomer: δ 7.46 (d, *J* = 7.0 Hz, 0.35H), 7.06 (d, *J* = 8.5 Hz, 0.39H), 6.34 (d, *J* = 3.0 Hz, 0.35H), 5.19 (dd, *J* = 8.0, 2.5 Hz, 0.34H), 4.52 (t, *J* = 2.5 Hz, 0.33H), 3.50 (s, 1H);

^{13}C NMR ($CDCl_3$, 100 MHz): δ 193.7, 174.7, 155.1, 154.7, 145.3, 129.7, 129.3, 124.6, 122.5, 122.3, 115.8, 108.3, 100.5, 86.7, 80.9, 54.5, 35.9, 26.1, 25.3, 20.1;

Diagnostic signals for the minor isomer: δ 193.5, 155.4, 154.9, 143.9, 131.1, 125.1, 121.9, 114.0, 100.3, 86.4, 81.3, 36.0, 26.5, 25.6, 21.0;

MS (ESI) *m/z* calcd for $C_{21}H_{21}N_2O_4$ ([M + H]$^+$) 365.1501, found 365.1505.

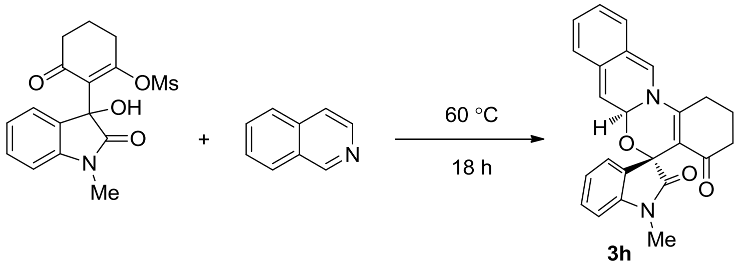

(3'R,6aS)-1'-Methyl-2,3-dihydro-1*H*-spiro[benzo[4,5][1,3]oxazino[3,2-b]isoquinoline-5,3'-indoline]-2',4(6a*H*)-dione (3h_Table 2, entry 8): According to the general procedure, compound **6b** (351 mg, 1.0 mmol) was dissolved in isoquinoline (1.0 mL) and stirred at 60 °C for 18 h. After the reaction was done, 1.0 mL of cold MeOH was added to allow the product to precipitate out. Then the mixture was filtered and the solid was purified by chromatography on SiO_2 using CH_2Cl_2/MeOH (60:1) as the eluent to afford 251 mg (65% yield) of the title compound as a colorless solid.

R_f (EtOAc/hexanes 6:1): 0.39;

IR (neat): 3008, 2952, 1726, 1634, 1562, 1402, 1308, 751 cm^{-1};

^1H NMR ($CDCl_3$, 400 MHz, 2:1): δ 7.41–7.32 (m, 2H), 7.28–7.21 (m, 1H), 7.20–6.98 (m, 4H), 6.93–6.71 (m, 3H), 5.83 (d, *J* = 8.0 Hz, 1H), 3.27 (s, 3H), 2.91 (dt, *J* = 16.4, 6.0 Hz, 1H), 2.72–2.56 (m, 1H), 2.34–2.28 (m, 2H), 2.19–2.11 (m, 2H);

Diagnostic signals for the minor isomer: δ 5.85 (d, *J* = 8.0 Hz, 0.50H), 3.31 (s, 1.6H), 3.03 (dt, *J* = 17.2, 4.8 Hz, 0.57H);

^{13}C NMR ($CDCl_3$, 100 MHz): δ 193.4, 175.2, 155.1, 145.4, 129.8, 129.4, 129.2, 127.8, 127.1, 127.0, 124.8, 122.6, 121.8, 114.5, 108.3, 105.1, 79.9, 35.8, 26.3, 25.4, 20.2;

Diagnostic signals for the minor isomer: δ 193.2, 174.6, 144.2, 129.9, 129.3, 127.0, 126.9, 124.8, 123.5, 122.1, 112.3, 108.4, 105.2, 80.2, 26.6, 25.5, 21.1;

MS (ESI) *m*/*z* calcd for $C_{24}H_{21}N_2O_3$ ($[M + H]^+$) 385.1552, found 385.1567.

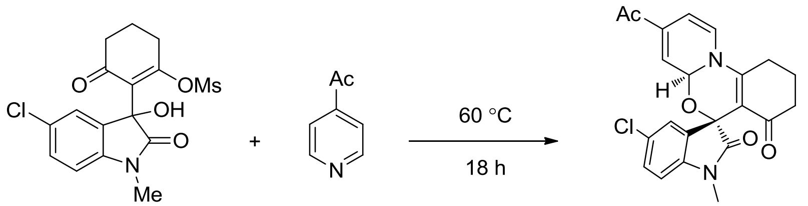

3i

(3'R,4aS)-3-Acetyl-5'-chloro-1'-methyl-9,10-dihydro-4a*H*-spiro[benzo[d]pyrido[2,1-b][1,3]oxazine-6,3'-indoline]-2',7(8*H*)-dione (3i_Table 2, entry 9): According to the general procedure, compound **6d** (386 mg, 1.0 mmol) was dissolved in 4-acetylpyridine (1.0 mL) and stirred at 60 °C for 18 h. After the reaction was complete, 1.0 mL of cold MeOH was added to allow the product to precipitate out. Then the mixture was filtered and the solid was purified by chromatography on SiO_2 using CH_2Cl_2/MeOH (60:1) as the eluent to afford 276 mg (67% yield) of the title compound as a colorless solid.

R_f (EtOAc/hexanes 6:1): 0.37;

IR (neat): 3083, 2953, 1711, 1638, 1563, 1364, 1107, 958, 753 cm^{-1};

^1H NMR ($CDCl_3$, 500 MHz, 8:1): δ 7.26 (dd, J = 8.0, 2.0 Hz, 1H), 7.01 (d, J = 2.5 Hz, 1H), 6.88 (d, J = 3.5 Hz, 1H), 6.83 (d, J = 8.0 Hz, 1H), 6.77 (d, J = 8.0 Hz, 1H), 6.34–6.32 (m, 1H), 5.93 (dd, J = 8.0, 1.5 Hz, 1H), 3.25 (s, 3H), 3.02 (dt, J = 17.5, 4.5 Hz, 1H), 2.64–2.57 (m, 1H), 2.35 (s, 3H), 2.35–2.31 (m, 2H), 2.22–2.16 (m, 1H), 2.05–1.98 (m, 1H);

Diagnostic signals for the minor isomer: δ 7.32 (dd, J = 8.0, 2.0 Hz, 0.15H), 6.80 (d, J = 8.5 Hz, 0.14H), 6.39–6.38 (m, 0.13H), 5.88 (dd, J = 8.0, 1.5 Hz, 0.10H), 2.95–2.88 (m, 0.13H);

^{13}C NMR ($CDCl_3$, 100 MHz): δ 195.6, 193.7, 174.1, 155.1, 144.1, 135.7, 130.5, 129.9, 128.1, 124.1, 123.0, 122.9, 115.1, 109.5, 98.2, 79.0, 35.9, 26.4, 25.3, 25.2, 20.1;

Diagnostic signals for the minor isomer: δ 195.5, 193.6, 155.3, 135.8, 127.5, 124.4, 124.1, 122.5, 113.1, 109.4, 98.3, 79.6, 35.9, 26.8, 25.4, 20.9;

MS (ESI) m/z calcd for $C_{22}H_{20}ClN_2O_4$ ($[M + H]^+$) 411.1112, found 411.1103.

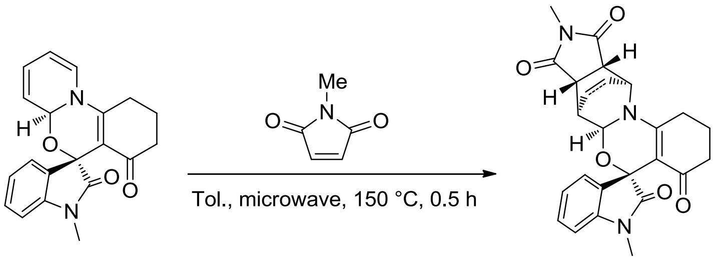

8a

Diels-Alder Reaction, Compound 8a: According to the general procedure, compound **3a** (50.1 mg, 0.15 mmol) and *N*-methylmaleimide (20.0 mg, 0.18 mmol) in 0.3 mL toluene were subjected to microwave reaction for 0.5 h at 150 °C. The solvent was then removed under reduced pressure and the residue was purified by chromatography on SiO_2 using CH_2Cl_2/MeOH (30:1) as the eluent to afford 45.1 mg (67% yield) of the title compound as a colorless solid.

R_f (EtOAc): 0.22;

IR (neat): 2950, 1696, 1558, 1432, 1340, 728 cm^{-1};

^{1}H NMR ($CDCl_3$, 500 MHz): δ 7.30–7.26 (m, 1H), 6.97–6.90 (m, 2H), 6.83 (d, *J* = 8.0 Hz, 1H), 6.43 (t, *J* = 7.0 Hz, 1H), 6.36 (t, *J* = 7.0 Hz, 1H), 5.50 (s, 1H), 5.04–5.00 (m, 1H), 3.53–3.50 (m, 1H), 3.48–3.44 (m, 1H), 3.38–3.22 (m 1H), 3.23 (s, 3H), 2.94–2.89 (m, 1H), 2.89 (s, 3H), 2.50–2.44 (m, 1H), 2.23–2.20 (m, 2H), 2.13–2.07 (m, 1H), 1.95–1.89 (m, 1H);

^{13}C NMR ($CDCl_3$, 100 MHz): δ 192.1, 177.3, 175.6, 175.3, 156.2, 144.9, 131.9, 131.1, 129.9, 129.7, 122.5, 121.9, 109.1, 108.4, 77.5, 48.0, 45.5, 37.2, 35.5, 35.4, 26.2, 25.6, 24.9, 20.1;

MS (ESI) *m*/*z* calcd for $C_{25}H_{24}N_3O_5$ ($[M + H]^+$) 446.1716, found 446.1727.

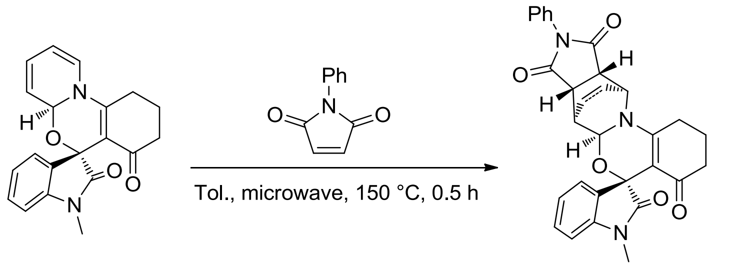

8b

Diels-Alder Reaction, Compound 8b: According to the general procedure, compound **3a** (50.1 mg, 0.15 mmol) and *N*-phenylmaleimide (31.2 mg, 0.18 mmol) in 0.3 mL toluene were subjected to microwave reaction for 0.5 h at 150 °C. The solvent was then removed under reduced pressure and the residue was purified by chromatography on SiO_2 using CH_2Cl_2/MeOH (30:1) as the eluent to afford 45.1 mg (70% yield) of the title compound as a colorless solid.

R_f (EtOAc): 0.25;

IR (neat): 2950, 1707, 1559, 1445, 1383, 1184, 726 cm^{-1};

^1H NMR ($CDCl_3$, 400 MHz): δ 7.47–7.36 (m, 3H), 7.32–7.27 (m, 1H), 7.16–7.12 (m, 2H), 6.99–6.94 (m, 2H), 6.85 (d, *J* = 8.0 Hz, 1H), 6.59–6.55 (m, 1H), 6.52–6.47 (m, 1H), 5.55 (d, *J* = 2.4 Hz, 1H), 5.11 (t, *J* = 4.4 Hz, 1H), 3.65–3.62 (m, 2H), 3.55 (dd, *J* = 8.0, 4.0 Hz, 1H), 3.24 (s, 3H), 2.96 (dt, *J* = 16.8, 4.8 Hz, 1H), 2.53–2.45 (m, 1H), 2.25–2.21 (m, 2H), 2.15–2.09 (m, 1H), 1.98–1.89 (m, 1H);

^{13}C NMR ($CDCl_3$, 100 MHz): δ 192.2, 176.4, 175.6, 174.4, 156.3, 144.9, 132.1, 131.4, 131.3, 130.0, 129.7, 129.2, 128.9, 126.2, 122.6, 122.0, 109.2, 108.4, 77.5, 48.2, 45.5, 37.6, 35.6, 35.4, 26.2, 25.6, 20.2;

MS (ESI) *m*/*z* calcd for $C_{30}H_{26}N_3O_5$ ($[M + H]^+$) 508.1872, found 508.1874.

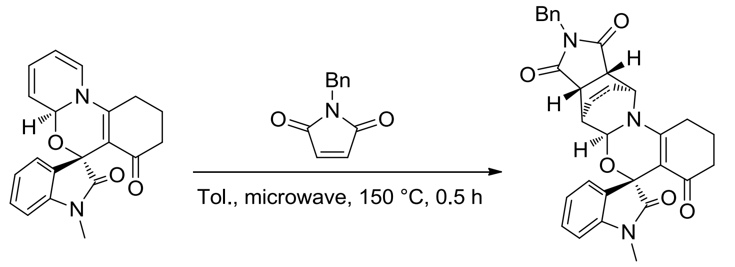

8c

Diels-Alder Reaction, Compound 8c: According to the general procedure, compound **3a** (50.1 mg, 0.15 mmol) and *N*-benzylmaleimide (33.7 mg, 0.18 mmol) in 0.3 mL toluene were subjected to microwave reaction for 0.5 h at 150 °C. The solvent was then removed under reduced pressure and the residue was purified by chromatography on SiO_2 using CH_2Cl_2/MeOH (30:1) as the eluent to afford 46.8 mg (60% yield) of the title compound as a colorless solid.

R_f (EtOAc): 0.28;

IR (neat): 2944, 1698, 1613, 1560, 1446, 1339, 728 cm^{-1};

^1H NMR (DMSO-d_6, 500 MHz): δ 7.32–7.23 (m, 4H), 7.16 (d, *J* = 7.0 Hz, 2H), 7.09 (d, *J* = 7.0 Hz, 1H), 6.96–6.90 (m, 2H), 6.47–6.43 (m, 1H), 6.31 (t, *J* = 8.0 Hz, 1H), 5.14 (d, *J* = 3.0 Hz, 1H), 5.10 (t, *J* = 4.5 Hz, 1H), 4.48 (s, 2H), 3.78 (dd, *J* = 8.0, 4.0 Hz, 1H), 3.36–3.32 (m, 1H), 3.27 (dd, *J* = 8.0, 3.0 Hz, 1H), 3.09 (s, 3H), 3.01 (dt, *J* = 17.0, 4.5 Hz, 1H), 2.50–2.45 (m, 1H), 2.10–2.03 (m, 1H), 1.98–1.89 (m, 2H), 1.83–1.74 (m, 1H);

^{13}C NMR ($CDCl_3$, 75 MHz): δ 192.1, 176.9, 175.5, 174.9, 156.2, 144.9, 135.3, 131.8, 131.0, 129.9, 129.6, 128.6, 128.5, 128.0, 122.5, 122.0, 109.0, 108.4, 77.6, 48.0, 45.4, 42.5, 37.3, 35.5, 35.3, 26.2, 25.6, 20.1;

MS (ESI) *m*/*z* calcd for $C_{31}H_{28}N_3O_5$ ($[M + H]^+$) 522.2029, found 522.2012.

X-ray Crystal Structure Data for Compounds 6b, 3d and 8a

1. X-ray crystallographic data for compound 6b

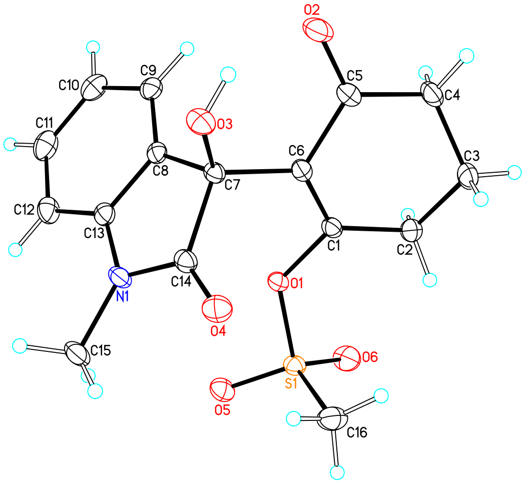

Crystals of compound **6b** suitable for X-ray analysis were obtained by slow evaporation from CH_2Cl_2/hexane. Crystallographic data have been deposited with the Cambridge Crystallographic Data Centre (CCDC# 742666). Copies of the data can be obtained free of charge on application to the CCDC, 12 Union Road, Cambridge CB21EZ, UK (fax: (+44)-1223-336-033; e-mail: deposit@ccdc.cam.ac.uk).

Table S1: Crystal data and structure refinement for compound **6b**.

Identification code	compound **6b**.	
Empirical formula	C16 H17 N O6 S	
Formula weight	351.37	
Temperature	173(2) K	
Wavelength	0.71073 Å	
Crystal system	Triclinic	
Space group	P-1	
Unit cell dimensions	a = 9.1725(4) Å	α= 98.295(2)°.
	b = 9.2077(4) Å	β= 101.709(2)°.
	c = 9.9640(4) Å	γ = 106.970(2)°.
Volume	769.31(6) Å^3	
Z	2	
Density (calculated)	1.517 Mg/m^3	
Absorption coefficient	0.245 mm^{-1}	
F(000)	368	

Crystal size	0.40 × 0.25 × 0.10 mm^3
Theta range for data collection	2.14 to 28.28°.
Index ranges	-12<=h<=12, -12<=k<=11, -13<=l<=13
Reflections collected	12447
Independent reflections	3788 [R(int) = 0.0297]
Completeness to theta = 28.28°	99.1 %
Absorption correction	Semi-empirical from equivalents
Max. and min. transmission	0.9759 and 0.9085
Refinement method	Full-matrix least-squares on F^2
Data / restraints / parameters	3788 / 0 / 285
Goodness-of-fit on F^2	1.078
Final R indices [I>2sigma(I)]	R1 = 0.0369, wR2 = 0.0951
R indices (all data)	R1 = 0.0455, wR2 = 0.1006
Largest diff. peak and hole	0.317 and −0.447 e.$Å^{-3}$

Table 2. Atomic coordinates (x 10^4) and equivalent isotropic displacement parameters ($Å^2 \times 10^3$) for compound **6b**. U(eq) is defined as one third of the trace of the orthogonalized U^{ij} tensor.

	x	y	z	U(eq)
S(1)	3350(1)	1408(1)	2797(1)	20(1)
O(1)	3807(1)	-162(1)	2753(1)	20(1)
O(2)	6703(1)	-3469(1)	2543(1)	33(1)
O(3)	4152(1)	-4069(1)	501(1)	23(1)
O(4)	3075(1)	-1597(1)	-403(1)	27(1)
O(5)	1684(1)	820(1)	2549(1)	26(1)
O(6)	4261(1)	2490(1)	4069(1)	31(1)
N(1)	1251(1)	-2814(1)	700(1)	20(1)
C(1)	5306(1)	-292(1)	2925(1)	18(1)
C(2)	6720(1)	1032(1)	3783(1)	25(1)
C(3)	8217(1)	759(2)	3552(1)	27(1)
C(4)	8203(1)	-858(1)	3702(1)	24(1)
C(5)	6731(1)	-2115(1)	2810(1)	21(1)
C(6)	5294(1)	-1710(1)	2350(1)	17(1)
C(7)	3797(1)	-2980(1)	1443(1)	17(1)
C(8)	2709(1)	-3769(1)	2269(1)	17(1)

C(9)	2963(1)	-4570(1)	3308(1)	21(1)
C(10)	1694(2)	-5273(1)	3832(1)	28(1)
C(11)	228(2)	-5145(2)	3324(1)	30(1)
C(12)	-40(1)	-4321(1)	2284(1)	26(1)
C(13)	1225(1)	-3649(1)	1772(1)	20(1)
C(14)	2706(1)	-2341(1)	460(1)	20(1)
C(15)	-79(1)	-2419(2)	-6(2)	28(1)
C(16)	3878(2)	2052(2)	1346(1)	30(1)

2. X-ray crystallographic data for compound 3d

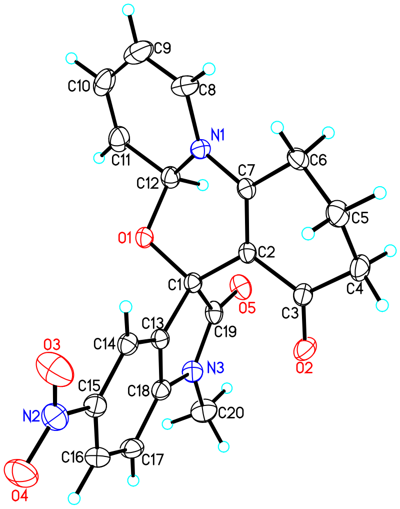

Crystals of compound **3d** suitable for X-ray analysis were obtained by slow evaporation from CH_2Cl_2/hexane. Crystallographic data have been deposited with the Cambridge Crystallographic Data Centre (CCDC# 742666). Copies of the data can be obtained free of charge on application to the CCDC, 12 Union Road, Cambridge CB21EZ, UK (fax: (+44)-1223-336-033; e-mail: deposit@ccdc.cam.ac.uk).

Table S2: Crystal data and structure refinement for compound **3d**.

Identification code	compound **3d**
Empirical formula	C21 H19 Cl2 N3 O5
Formula weight	464.29
Temperature	173(2) K
Wavelength	0.71073 Å
Crystal system	Monoclinic
Space group	P2(1)/n

Unit cell dimensions	a = 7.7598(2) Å	α= 90°.
	b = 20.4280(7) Å	β= 94.966(2)°.
	c = 13.1671(4) Å	γ = 90°.
Volume	2079.38(11) $Å^3$	
Z	4	
Density (calculated)	1.483 Mg/m^3	
Absorption coefficient	0.352 mm^{-1}	
F(000)	960	
Crystal size	0.60 × 0.40 × 0.35 mm^3	
Theta range for data collection	1.84 to 28.28°.	
Index ranges	-9<=h<=10, -27<=k<=27, -17<=l<=17	
Reflections collected	21399	
Independent reflections	5171 [R(int) = 0.0334]	
Completeness to theta = 28.28°	100.0 %	
Absorption correction	Semi-empirical from equivalents	
Max. and min. transmission	0.8867 and 0.8165	
Refinement method	Full-matrix least-squares on F^2	
Data / restraints / parameters	5171 / 0 / 356	
Goodness-of-fit on F^2	1.046	
Final R indices [I>2sigma(I)]	R1 = 0.0422, wR2 = 0.1100	
R indices (all data)	R1 = 0.0575, wR2 = 0.1199	
Largest diff. peak and hole	0.461 and −0.604 e.$Å^{-3}$	

Table 2. Atomic coordinates (x 10^4) and equivalent isotropic displacement parameters ($Å^2$ × 10^3) for compound **3d**. U(eq) is defined as one third of the trace of the orthogonalized U^{ij} tensor.

	x	y	z	U(eq)
O(1)	8612(1)	1653(1)	2823(1)	25(1)
O(2)	12395(1)	696(1)	1316(1)	35(1)
O(3)	8599(2)	-780(1)	4492(1)	52(1)
O(4)	7065(2)	-1406(1)	3455(1)	48(1)
O(5)	9783(1)	1959(1)	717(1)	32(1)
N(1)	10904(1)	2123(1)	3863(1)	24(1)
N(2)	7896(2)	-903(1)	3644(1)	35(1)
N(3)	8539(1)	935(1)	609(1)	26(1)

C(1)	9719(1)	1266(1)	2248(1)	21(1)
C(2)	11580(1)	1324(1)	2670(1)	21(1)
C(3)	12833(2)	971(1)	2128(1)	25(1)
C(4)	14677(2)	968(1)	2580(1)	32(1)
C(5)	14812(2)	1044(1)	3724(1)	31(1)
C(6)	13860(2)	1647(1)	4041(1)	27(1)
C(7)	12069(2)	1686(1)	3515(1)	22(1)
C(8)	11104(2)	2423(1)	4811(1)	35(1)
C(9)	9892(2)	2816(1)	5132(1)	43(1)
C(10)	8310(2)	2921(1)	4508(1)	40(1)
C(11)	8076(2)	2665(1)	3579(1)	35(1)
C(12)	9415(2)	2261(1)	3135(1)	26(1)
C(13)	9008(1)	580(1)	2254(1)	22(1)
C(14)	8909(2)	156(1)	3048(1)	24(1)
C(15)	8056(2)	-434(1)	2829(1)	27(1)
C(16)	7357(2)	-598(1)	1857(1)	31(1)
C(17)	7491(2)	-171(1)	1051(1)	29(1)
C(18)	8309(2)	421(1)	1272(1)	24(1)
C(19)	9407(2)	1445(1)	1102(1)	25(1)
C(20)	8052(2)	920(1)	-481(1)	37(1)
Cl(1)	6916(1)	724(1)	6387(1)	82(1)
Cl(2)	8484(1)	1907(1)	7253(1)	54(1)
C(1S)	7380(3)	1544(1)	6178(1)	50(1)

3. X-ray crystallographic data for compound 8a

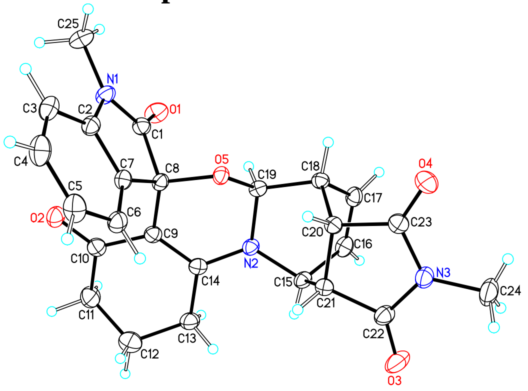

Crystals of compound **8a** suitable for X-ray analysis were obtained by slow evaporation from CH_2Cl_2/hexane. Crystallographic data have been deposited with the Cambridge Crystallographic

Data Centre (CCDC# 742666). Copies of the data can be obtained free of charge on application to the CCDC, 12 Union Road, Cambridge CB21EZ, UK (fax: (+44)-1223-336-033; e-mail: deposit@ccdc.cam.ac.uk).

Table S3: Crystal data and structure refinement for compound **8a.**

Identification code	compound **8a**	
Empirical formula	C26 H25 Cl2 N3 O5	
Formula weight	530.39	
Temperature	173(2) K	
Wavelength	0.71073 Å	
Crystal system	Monoclinic	
Space group	P2(1)/n	
Unit cell dimensions	a = 11.8113(5) Å	α= 90°.
	b = 11.9616(6) Å	β= 91.888(2)°.
	c = 20.0732(10) Å	γ = 90°.
Volume	2834.4(2) $Å^3$	
Z	4	
Density (calculated)	1.243 Mg/m^3	
Absorption coefficient	0.267 mm^{-1}	
F(000)	1104	
Crystal size	0.40 x 0.30 x 0.25 mm^3	
Theta range for data collection	1.97 to 28.28°.	
Index ranges	-15<=h<=15, -15<=k<=15, -26<=l<=26	
Reflections collected	33800	
Independent reflections	6998 [R(int) = 0.0390]	
Completeness to theta = 28.28°	99.4 %	
Absorption correction	Semi-empirical from equivalents	
Max. and min. transmission	0.9363 and 0.9007	
Refinement method	Full-matrix least-squares on F^2	
Data / restraints / parameters	6998 / 0 / 393	
Goodness-of-fit on F^2	1.081	
Final R indices [I>2sigma(I)]	R1 = 0.0506, wR2 = 0.1323	
R indices (all data)	R1 = 0.0674, wR2 = 0.1391	
Largest diff. peak and hole	0.777 and −0.440 e.$Å^{-3}$	

Table 2. Atomic coordinates (x 10^4) and equivalent isotropic displacement parameters ($Å^2$x 10^3) for compound **8a**. U(eq) is defined as one third of the trace of the orthogonalized U^{ij} tensor.

	x	y	z	U(eq)
O(1)	1027(1)	2942(1)	2496(1)	33(1)
O(2)	2837(1)	1804(1)	1288(1)	29(1)
O(3)	4287(1)	-1729(1)	5000(1)	44(1)
O(4)	448(1)	-1623(1)	4903(1)	39(1)
O(5)	1315(1)	512(1)	2983(1)	21(1)
N(1)	183(1)	1785(1)	1715(1)	26(1)
N(2)	3077(1)	849(1)	3543(1)	21(1)
N(3)	2358(1)	-1807(1)	5076(1)	27(1)
C(1)	950(1)	2045(1)	2212(1)	24(1)
C(2)	375(1)	706(1)	1464(1)	24(1)
C(3)	-185(1)	176(1)	938(1)	31(1)
C(4)	166(2)	-901(1)	789(1)	36(1)
C(5)	1033(1)	-1420(1)	1146(1)	34(1)
C(6)	1593(1)	-872(1)	1677(1)	26(1)
C(7)	1252(1)	198(1)	1825(1)	22(1)
C(8)	1678(1)	982(1)	2364(1)	20(1)
C(9)	2937(1)	1185(1)	2388(1)	22(1)
C(10)	3426(1)	1589(1)	1788(1)	26(1)
C(11)	4694(1)	1773(2)	1804(1)	38(1)
C(12)	5298(1)	935(2)	2248(1)	45(1)
C(13)	4859(1)	966(1)	2951(1)	29(1)
C(14)	3580(1)	984(1)	2955(1)	21(1)
C(15)	3562(1)	304(1)	4146(1)	23(1)
C(16)	3143(1)	923(1)	4743(1)	27(1)
C(17)	2029(1)	1002(1)	4761(1)	26(1)
C(18)	1410(1)	468(1)	4176(1)	21(1)
C(19)	1850(1)	1026(1)	3549(1)	21(1)
C(20)	1752(1)	-779(1)	4134(1)	21(1)
C(21)	3050(1)	-886(1)	4144(1)	22(1)
C(22)	3346(1)	-1516(1)	4776(1)	28(1)
C(23)	1396(1)	-1438(1)	4732(1)	26(1)

C(24)	2335(2)	-2462(1)	5686(1)	41(1)
C(25)	-679(1)	2543(1)	1455(1)	36(1)
Cl(1)	3244(1)	9187(1)	-478(1)	66(1)
Cl(2)	4225(1)	8971(1)	860(1)	60(1)
C(1S)	3451(2)	9826(2)	304(1)	52(1)

References:

[1] Perrin, D. D.; Armarego, W. L. F. *Purification of Laboratory Chemicals*, 3rd ed.; Pergamon Press: Oxford, 1988.

[2] Ferandin, Y.; Bettayeb, K.; Kritsanida, M.; Lozach, O.; Polychronopoulos, P.; Magiatis, P.; Skaltsounis, A.; Meijer, L. *J. Med. Chem.* **2006,** *49*, 4638. doi:10.1021/jm060314i

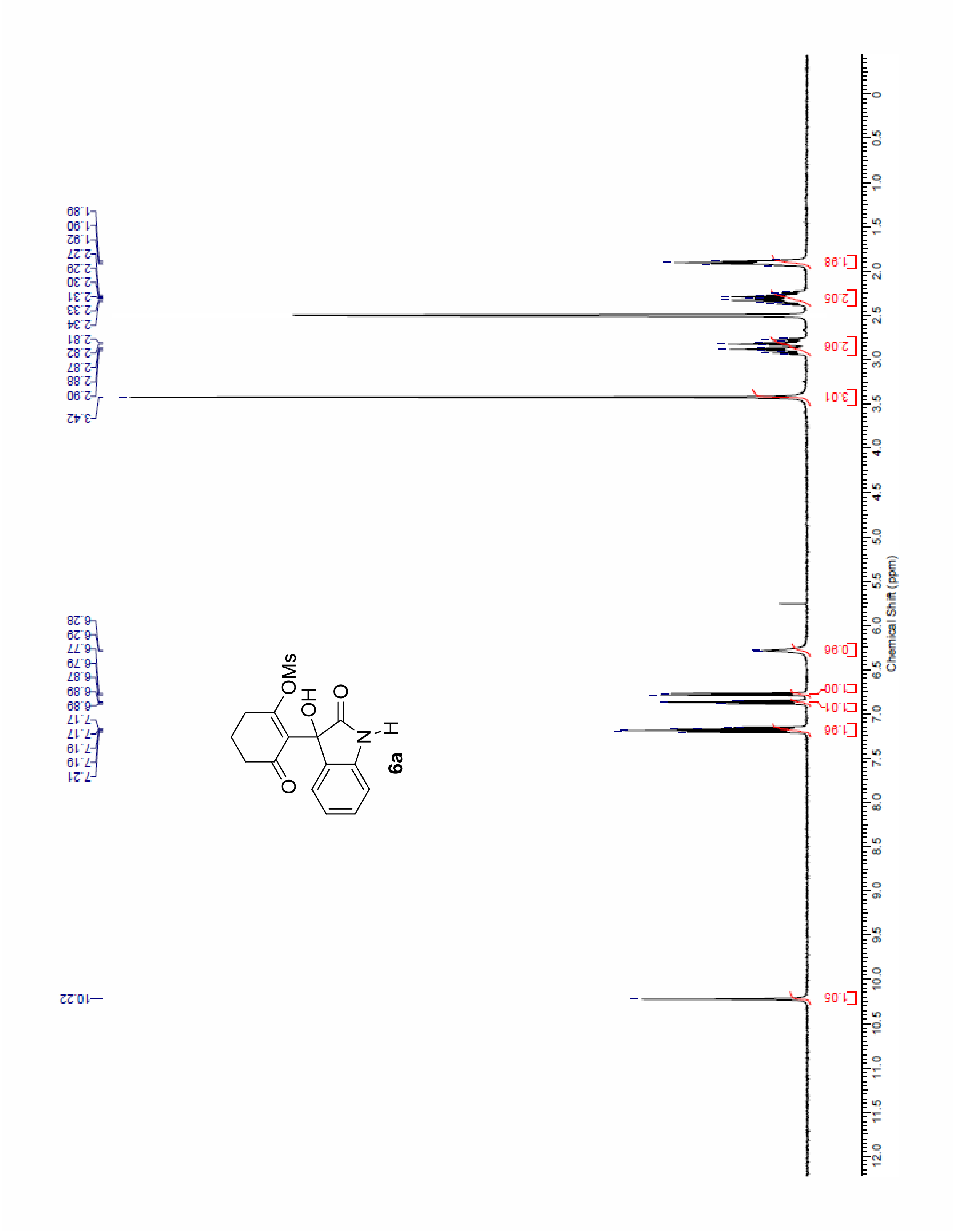

OMs
OH
O
N
H
6a
Chemical Shift (ppm)

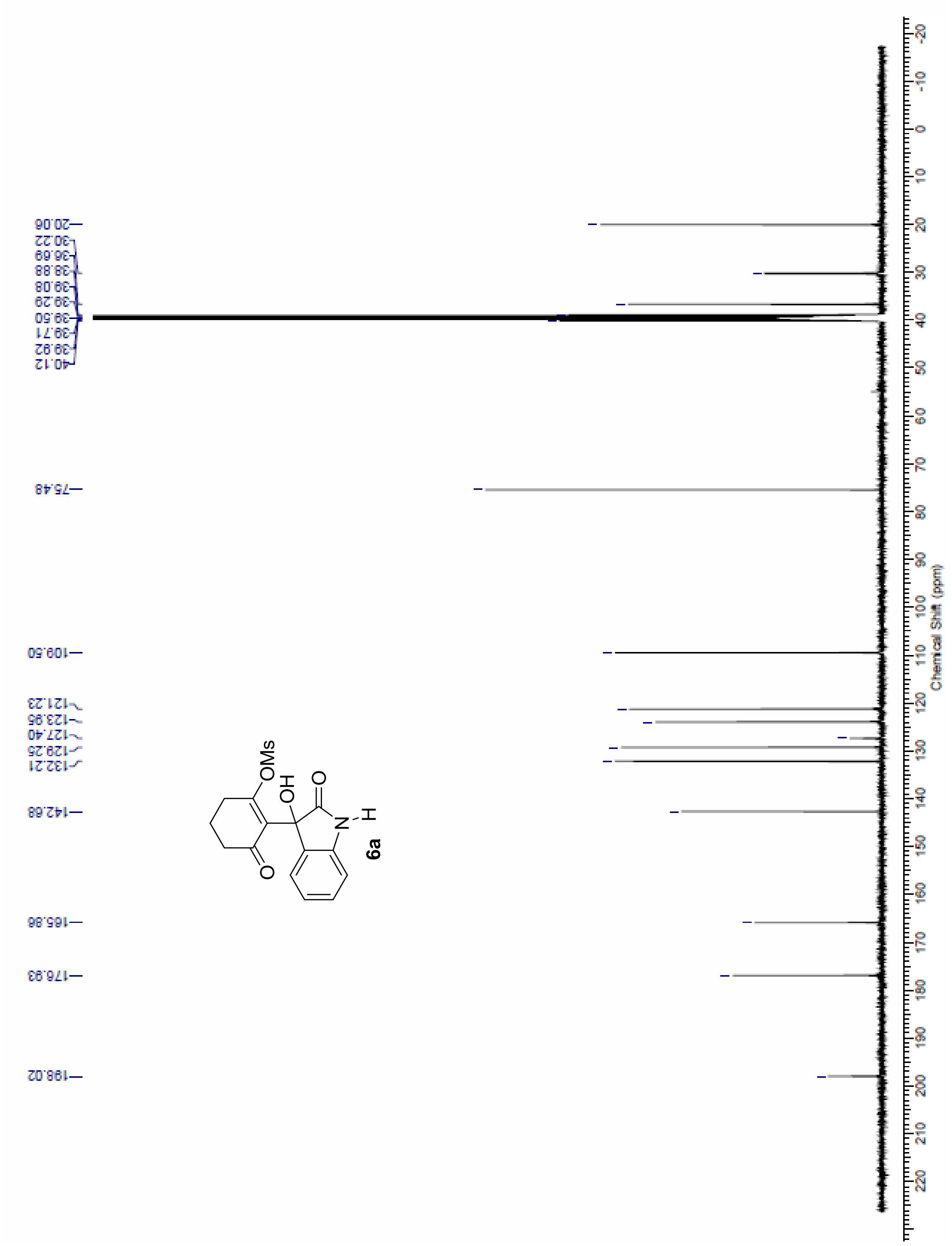

20.06
30.22
36.69
38.88
39.08
39.29
39.50
39.71
39.92
40.12
75.48
109.50
121.23
123.95
127.40
129.25
132.21
142.68
165.86
176.93
198.02
OMs
OH
O
N
H
6a
Chemical Shift (ppm)

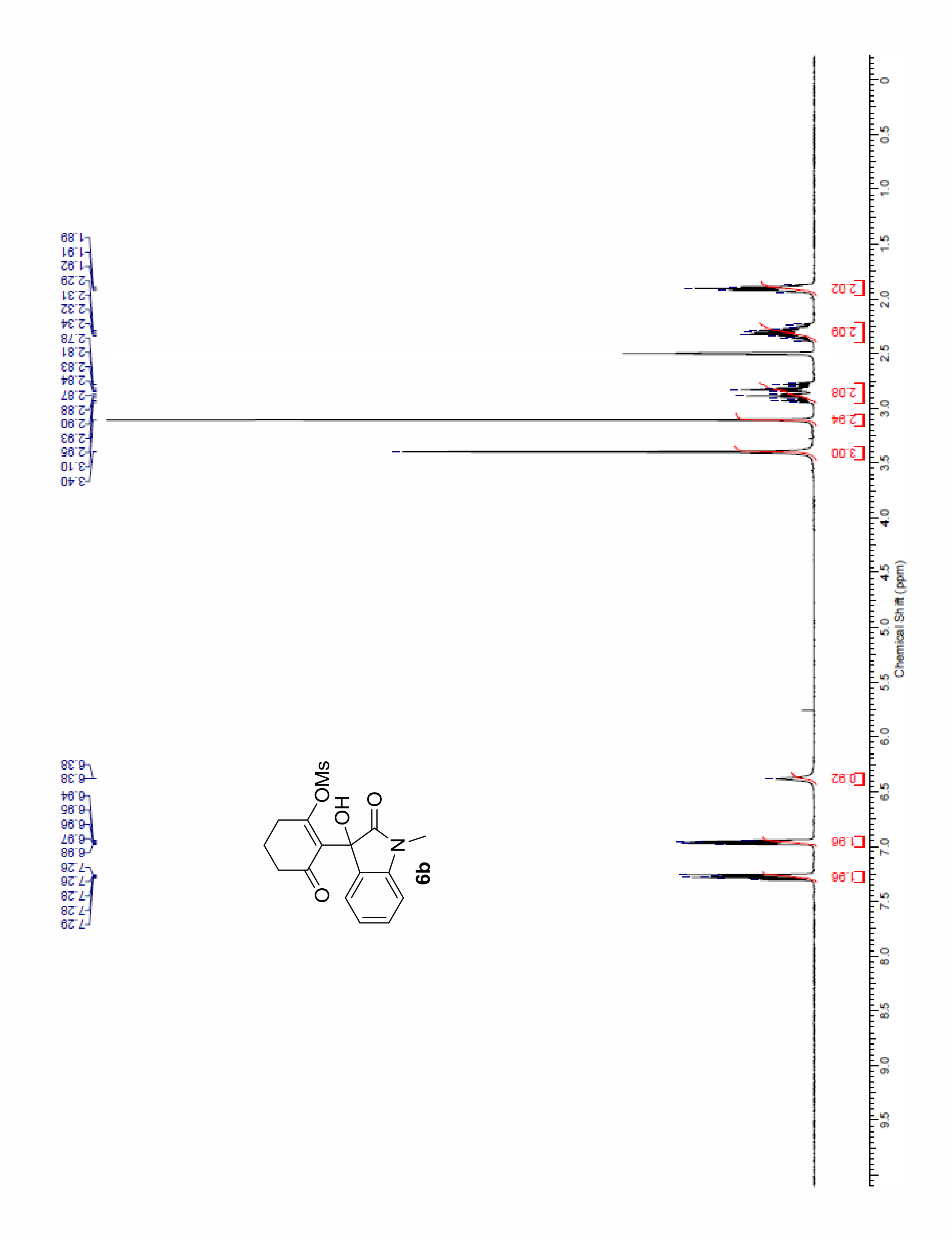
1.89
1.91
1.92
2.29
2.31
2.32
2.34
2.78
2.81
2.83
2.84
2.87
2.88
2.90
2.93
2.96
3.10
3.40
6.38
6.38
6.94
6.96
6.96
6.97
6.98
7.26
7.26
7.28
7.28
7.29
OMs
OH
O
N
O
6b
2.02
2.09
2.08
2.94
3.00
0.92
1.96
1.96
Chemical Shift (ppm)

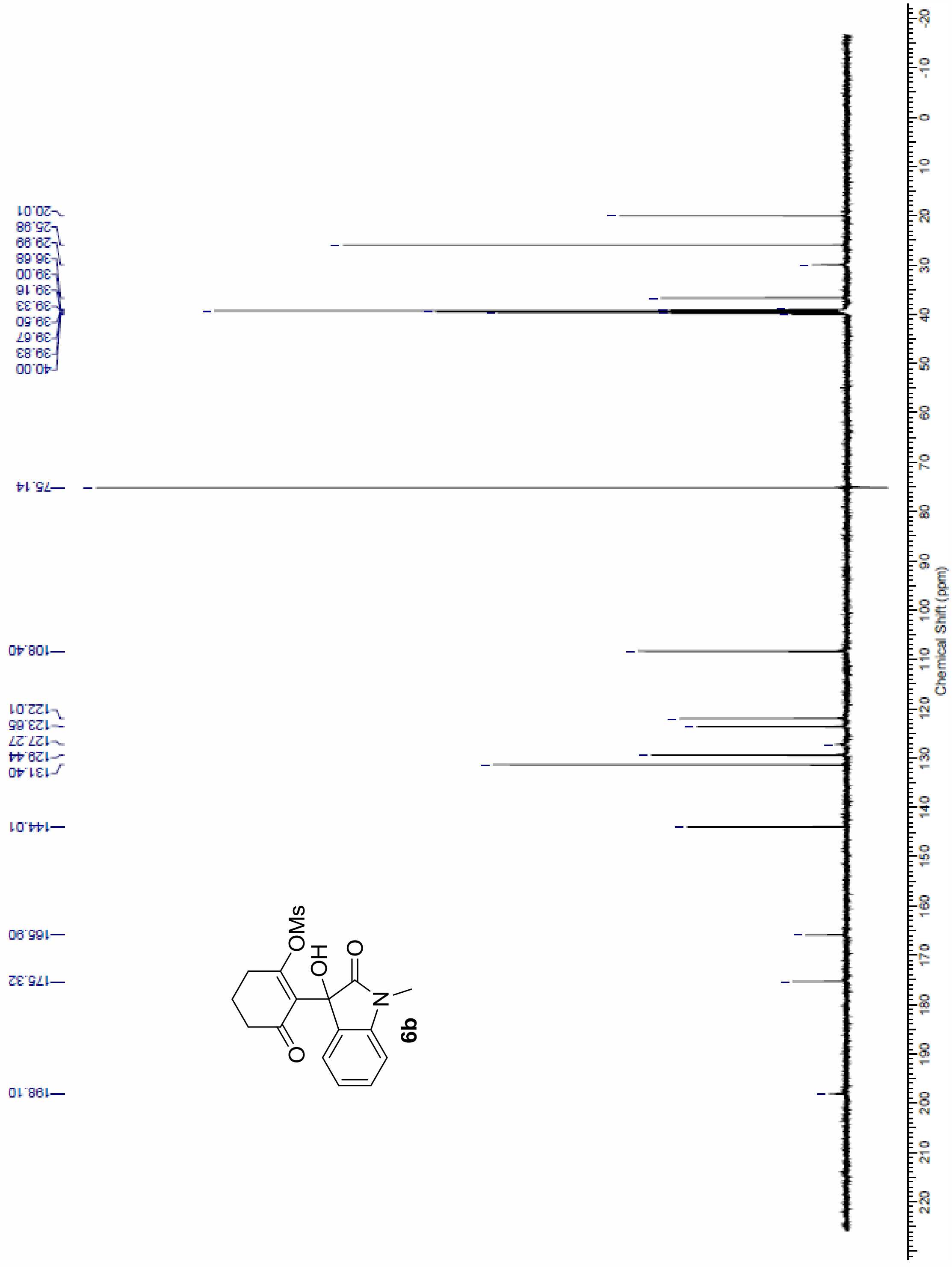
20.01
25.98
29.99
36.68
39.00
39.16
39.33
39.50
39.67
39.83
40.00
75.14
108.40
122.01
123.65
127.27
129.44
131.40
144.01
165.90
175.32
198.10
OMs
OH
O
N
6b
Chemical Shift (ppm)

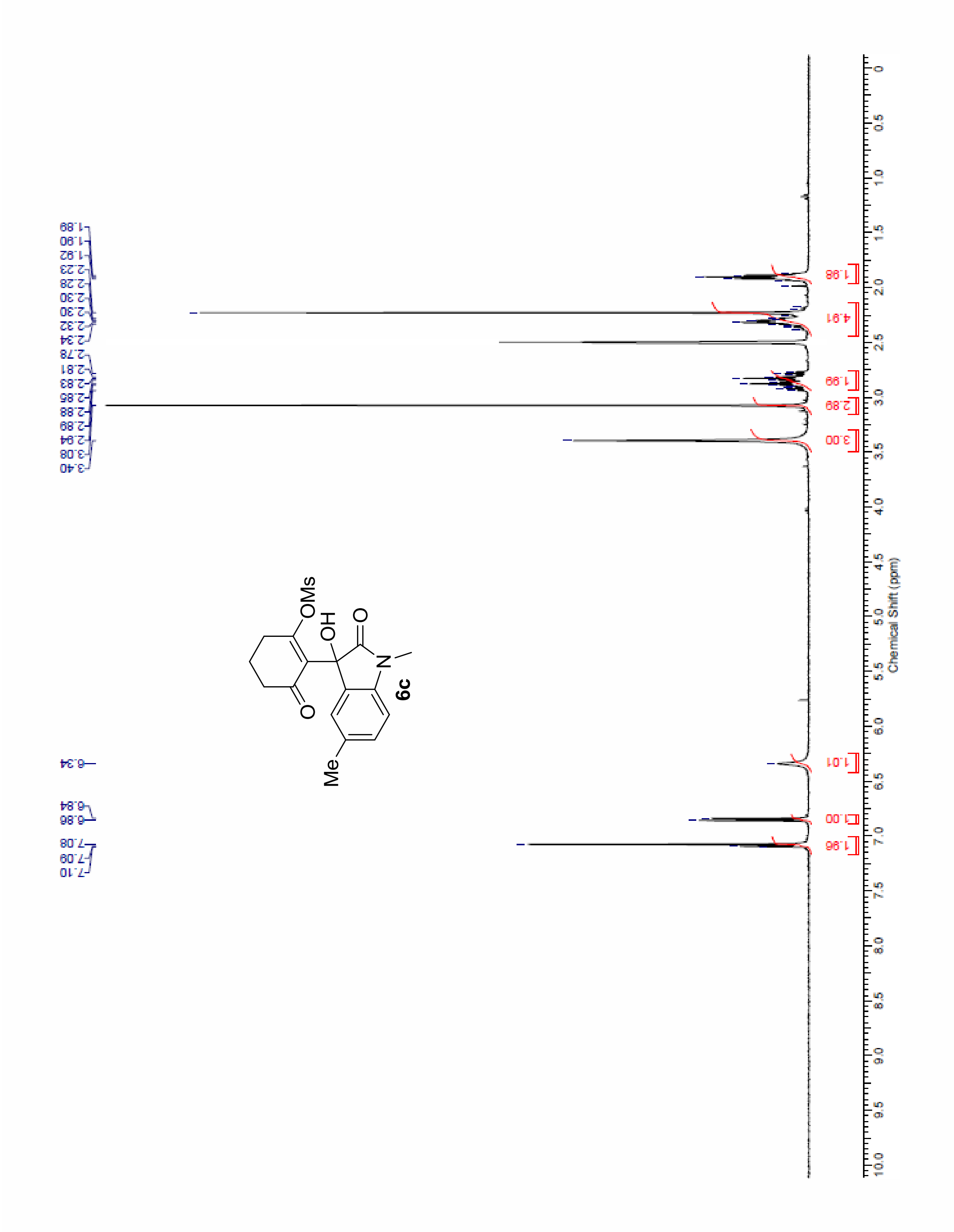
1.89
1.90
1.92
2.23
2.28
2.30
2.30
2.32
2.34
2.78
2.81
2.83
2.86
2.88
2.89
2.94
3.08
3.40
6.34
6.84
6.86
7.08
7.09
7.10
OMs
OH
O
O
N
Me
6c
1.98
4.91
1.99
2.89
3.00
1.01
1.00
1.96
0
0.5
1.0
1.5
2.0
2.5
3.0
3.5
4.0
4.5
5.0
5.5
6.0
6.5
7.0
7.5
8.0
8.5
9.0
9.5
10.0
Chemical Shift (ppm)

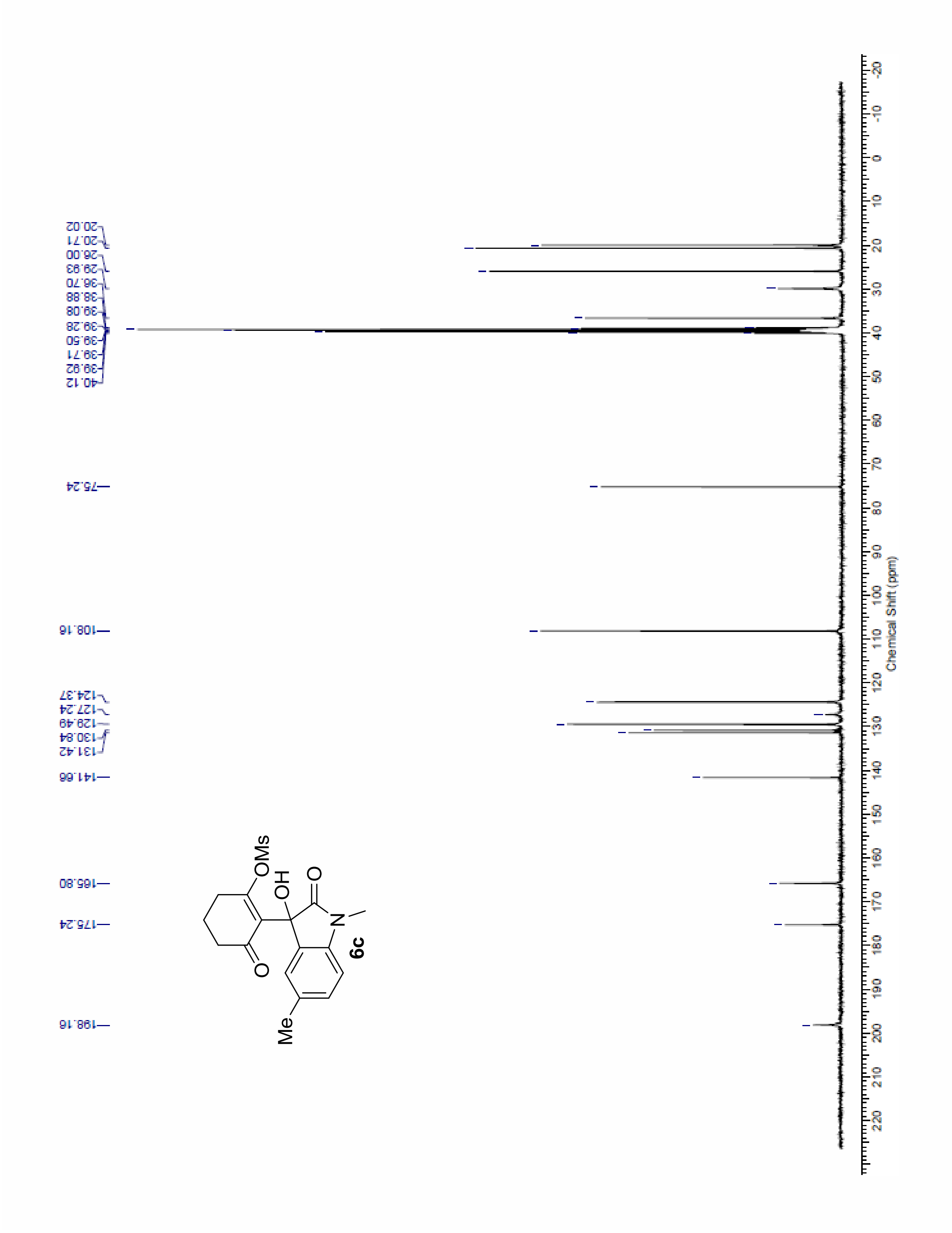
20.02
20.71
26.00
29.93
36.70
38.88
39.08
39.28
39.50
39.71
39.92
40.12
75.24
108.16
124.37
127.24
129.49
130.84
131.42
141.66
165.80
175.24
198.16
OMs
OH
O
N
O
Me
6c
Chemical Shift (ppm)

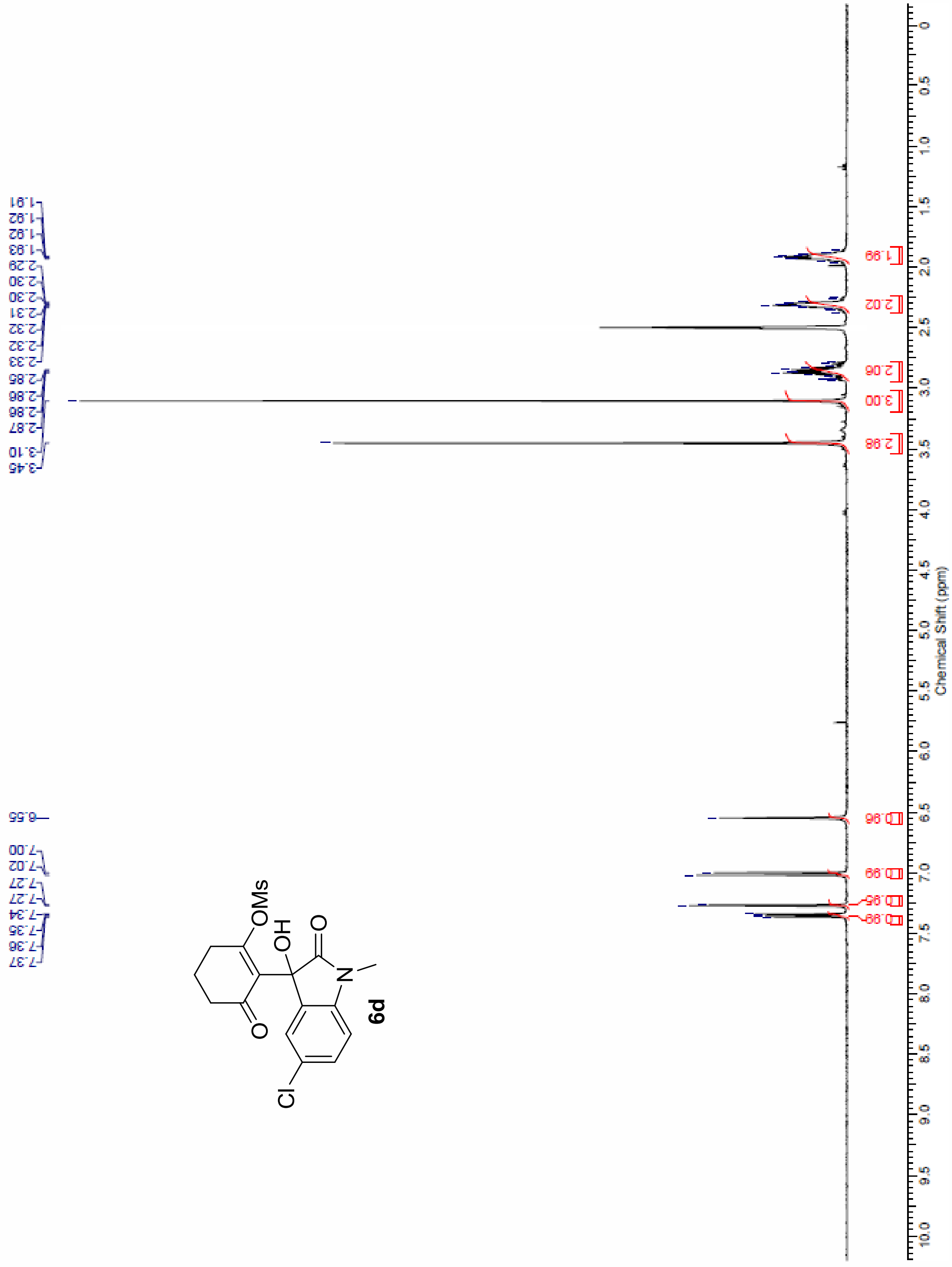

1.91
1.92
1.92
1.93
2.29
2.30
2.30
2.31
2.32
2.32
2.33
2.85
2.86
2.86
2.87
3.10
3.45
6.55
7.00
7.02
7.27
7.27
7.34
7.36
7.36
7.37
OMs
OH
O
N
Cl
6d
1.99
2.02
2.06
3.00
2.98
0.96
0.99
0.95
0.99
Chemical Shift (ppm)

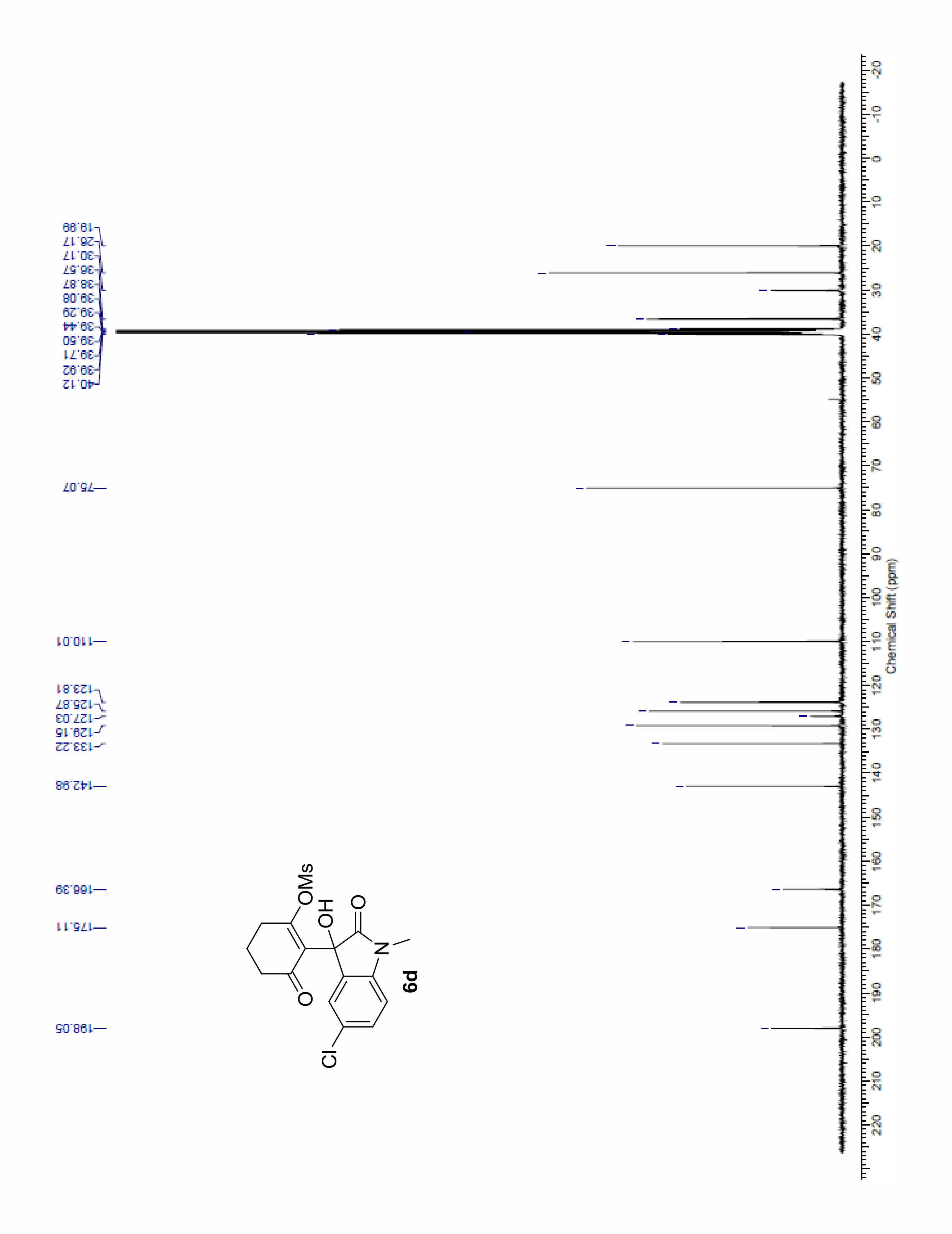
19.99
26.17
30.17
36.57
38.87
39.08
39.29
39.44
39.60
39.71
39.92
40.12
75.07
110.01
123.81
126.87
127.03
129.15
133.22
142.98
166.39
175.11
198.05
OMs
OH
O
N
O
Cl
6d
Chemical Shift (ppm)

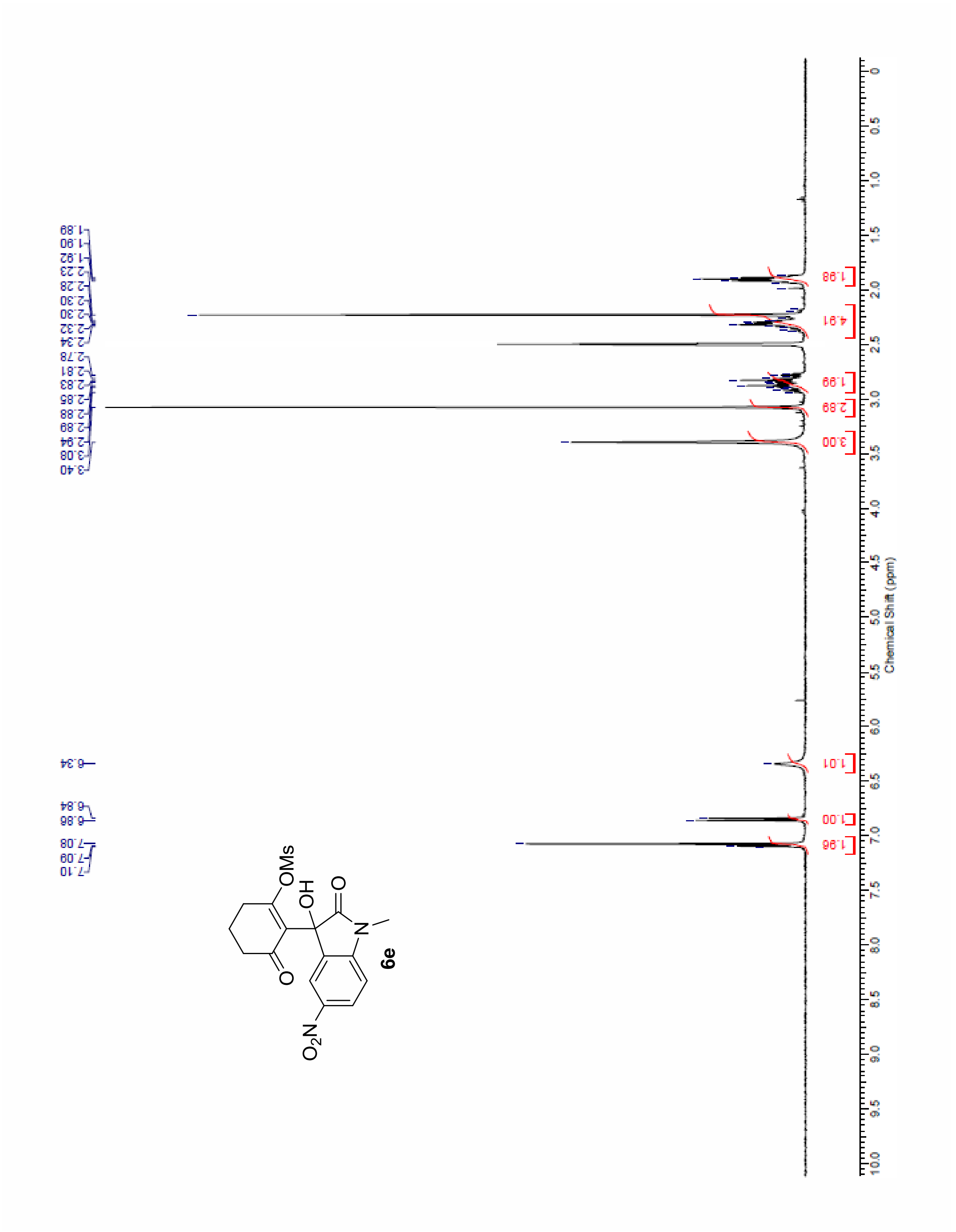
6e
Chemical Shift (ppm)

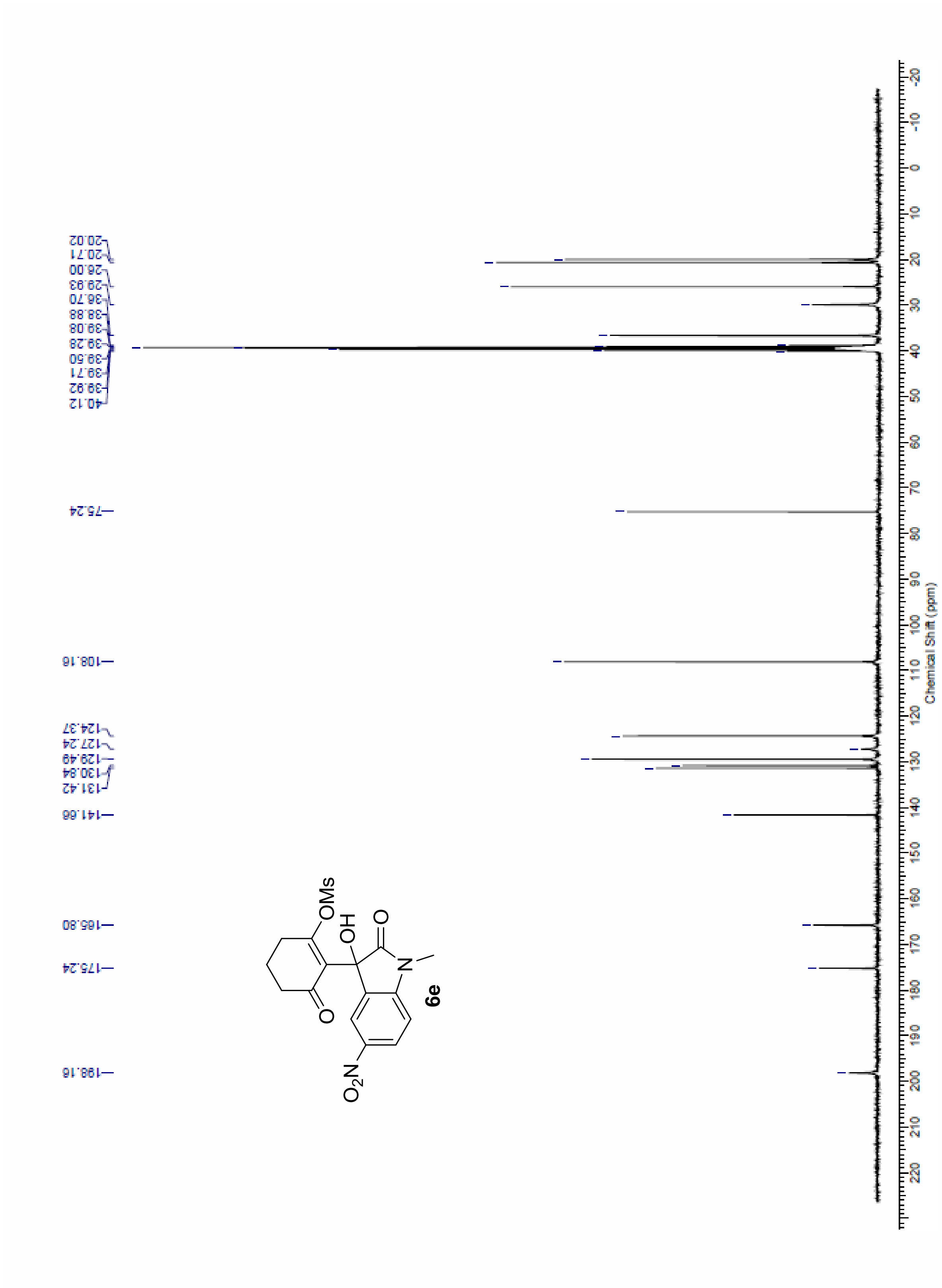

20.02
20.71
26.00
29.93
36.70
38.88
39.08
39.28
39.50
39.71
39.92
40.12
75.24
108.16
124.37
127.24
129.49
130.84
131.42
141.66
165.80
175.24
198.16
OMs
OH
O
N
O
O_2N
6e
Chemical Shift (ppm)

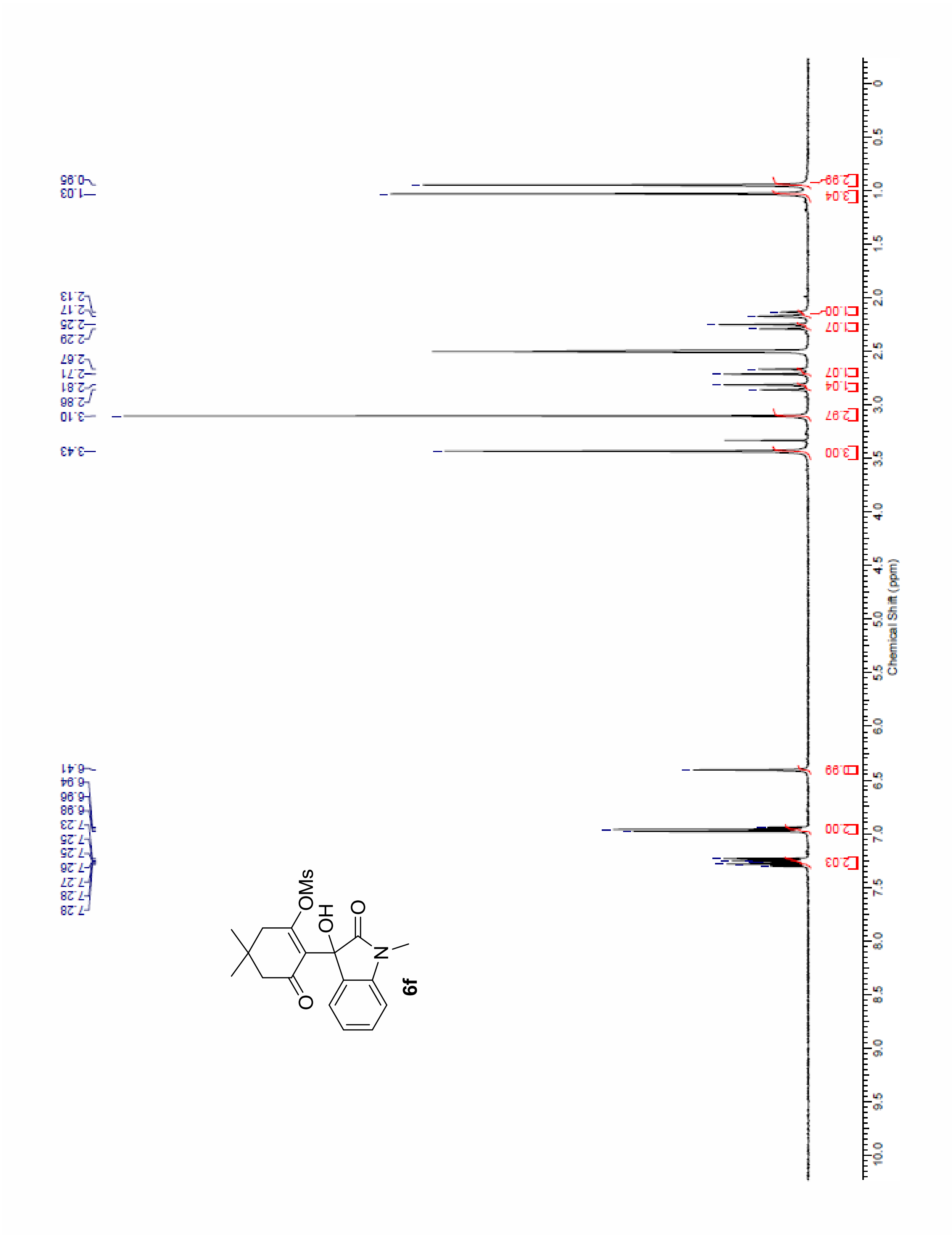

0.95
1.03
2.13
2.17
2.25
2.29
2.67
2.71
2.81
2.86
3.10
3.43
6.41
6.94
6.96
6.98
7.23
7.25
7.25
7.26
7.27
7.28
7.28
2.99
3.04
1.00
1.07
1.07
1.04
2.97
3.00
0.99
2.00
2.03
Chemical Shift (ppm)
OMs
OH
O
N
6f

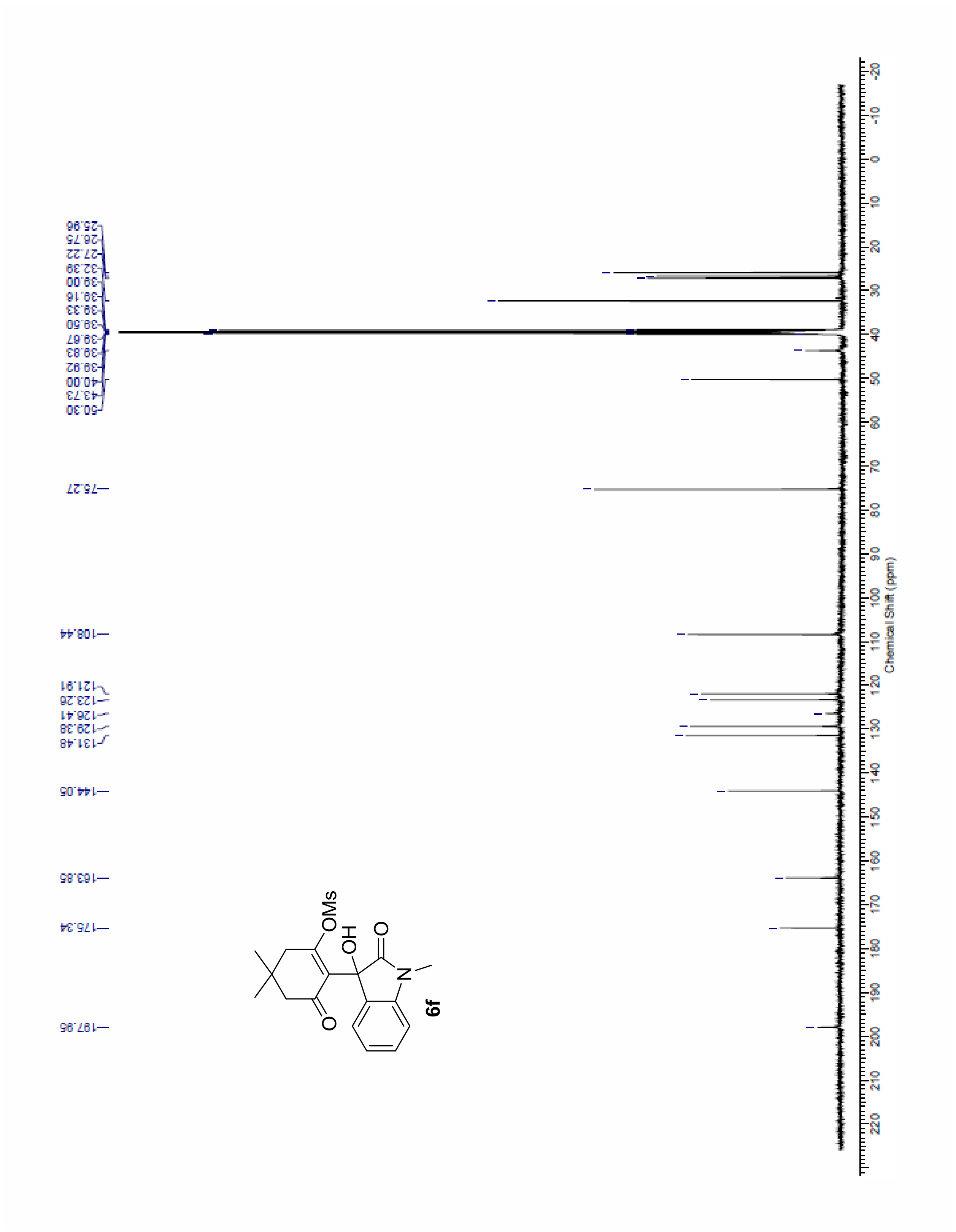
25.96
26.75
27.22
32.39
39.00
39.16
39.33
39.50
39.67
39.83
39.92
40.00
43.73
50.30
75.27
108.44
121.91
123.26
126.41
129.38
131.48
144.05
163.85
175.34
197.95
OMs
OH
O
N
6f
Chemical Shift (ppm)

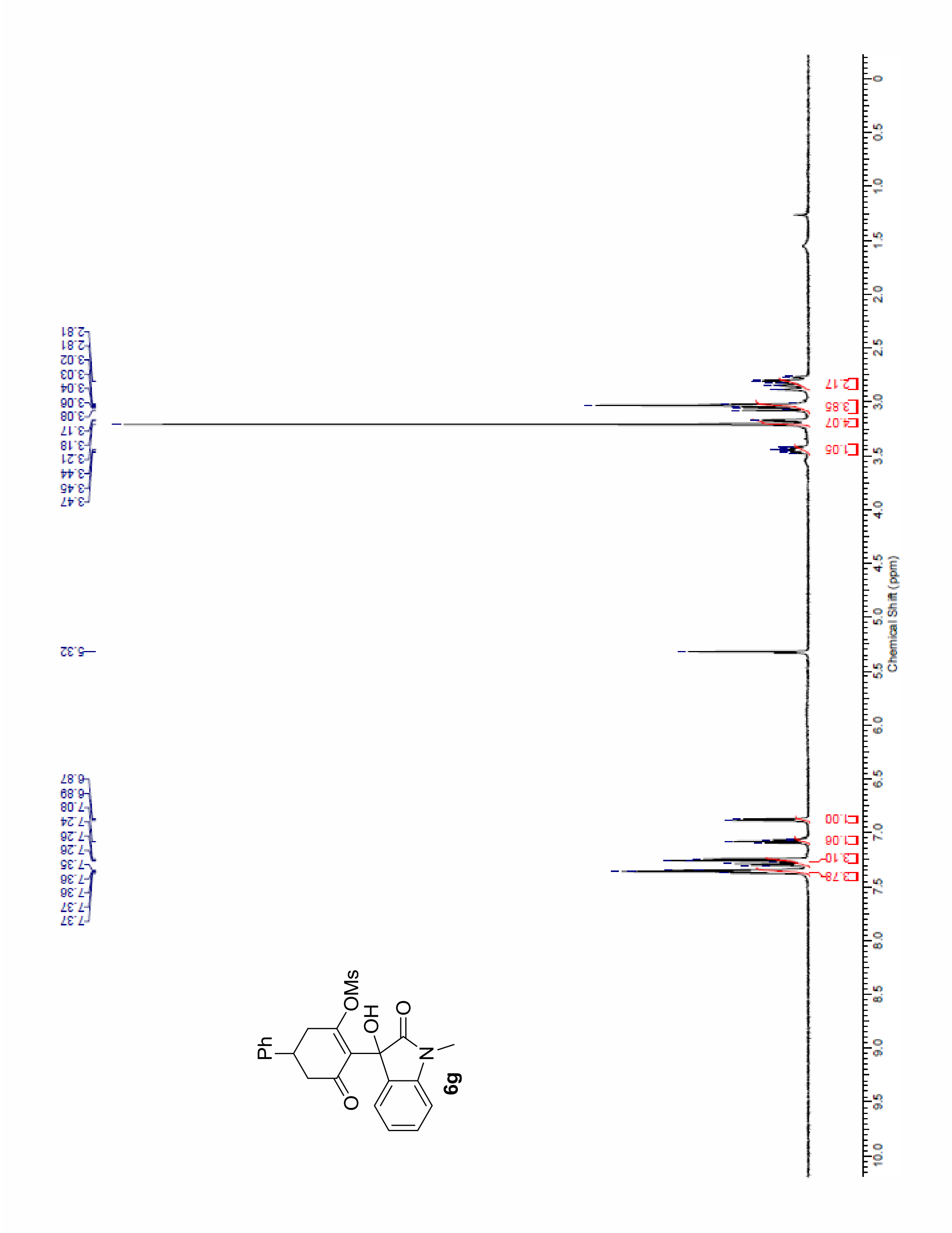
2.81
2.81
3.02
3.03
3.04
3.06
3.08
3.17
3.18
3.21
3.44
3.45
3.47
5.32
6.87
6.89
7.08
7.24
7.26
7.26
7.35
7.36
7.36
7.37
7.37
2.17
3.85
4.07
1.06
1.00
1.06
3.10
3.78
Ph
OMs
OH
O
O
N
6g
Chemical Shift (ppm)

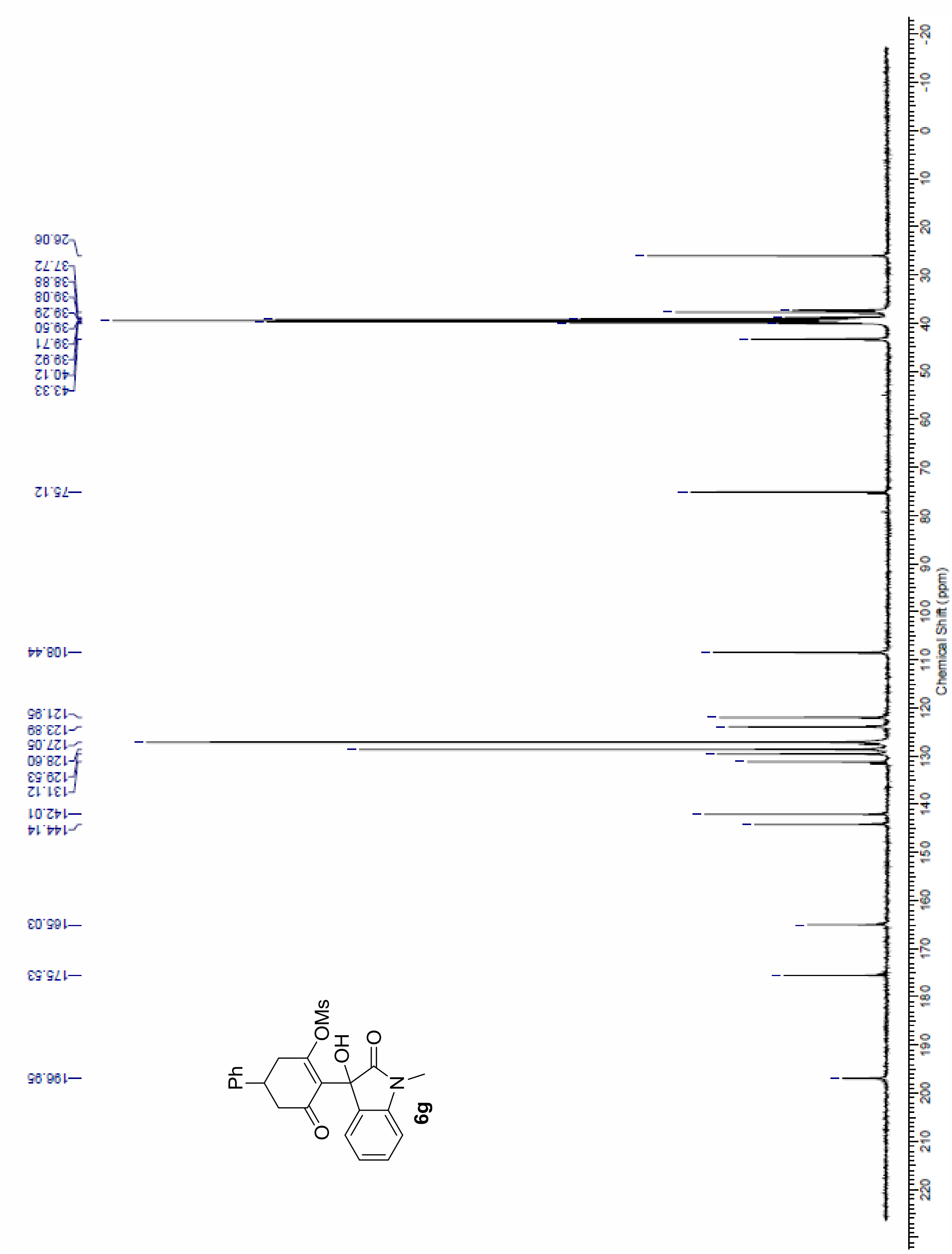
196.95
175.53
165.03
144.14
142.01
131.12
129.53
128.60
127.05
123.89
121.95
108.44
75.12
43.33
40.12
39.92
39.71
39.50
39.29
39.08
38.88
37.72
26.06
Chemical Shift (ppm)
Ph
OMs
OH
O
N
6g

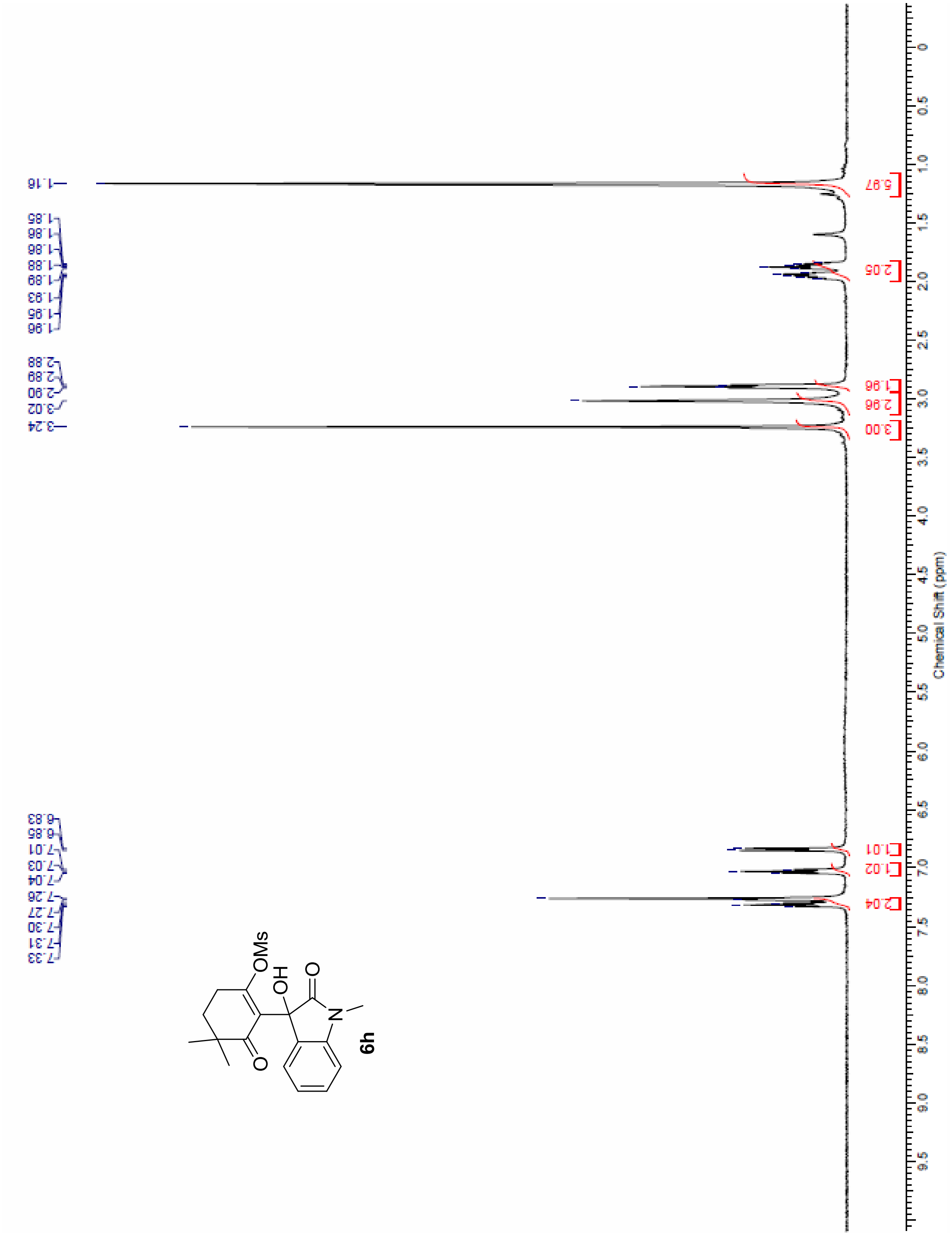
1.16
1.85
1.86
1.86
1.88
1.89
1.93
1.95
1.96
2.88
2.89
2.90
3.02
3.24
6.83
6.85
7.01
7.03
7.04
7.26
7.27
7.30
7.31
7.33
5.97
2.05
1.96
2.96
3.00
1.01
1.02
2.04
OMs
OH
O
N
6h
Chemical Shift (ppm)

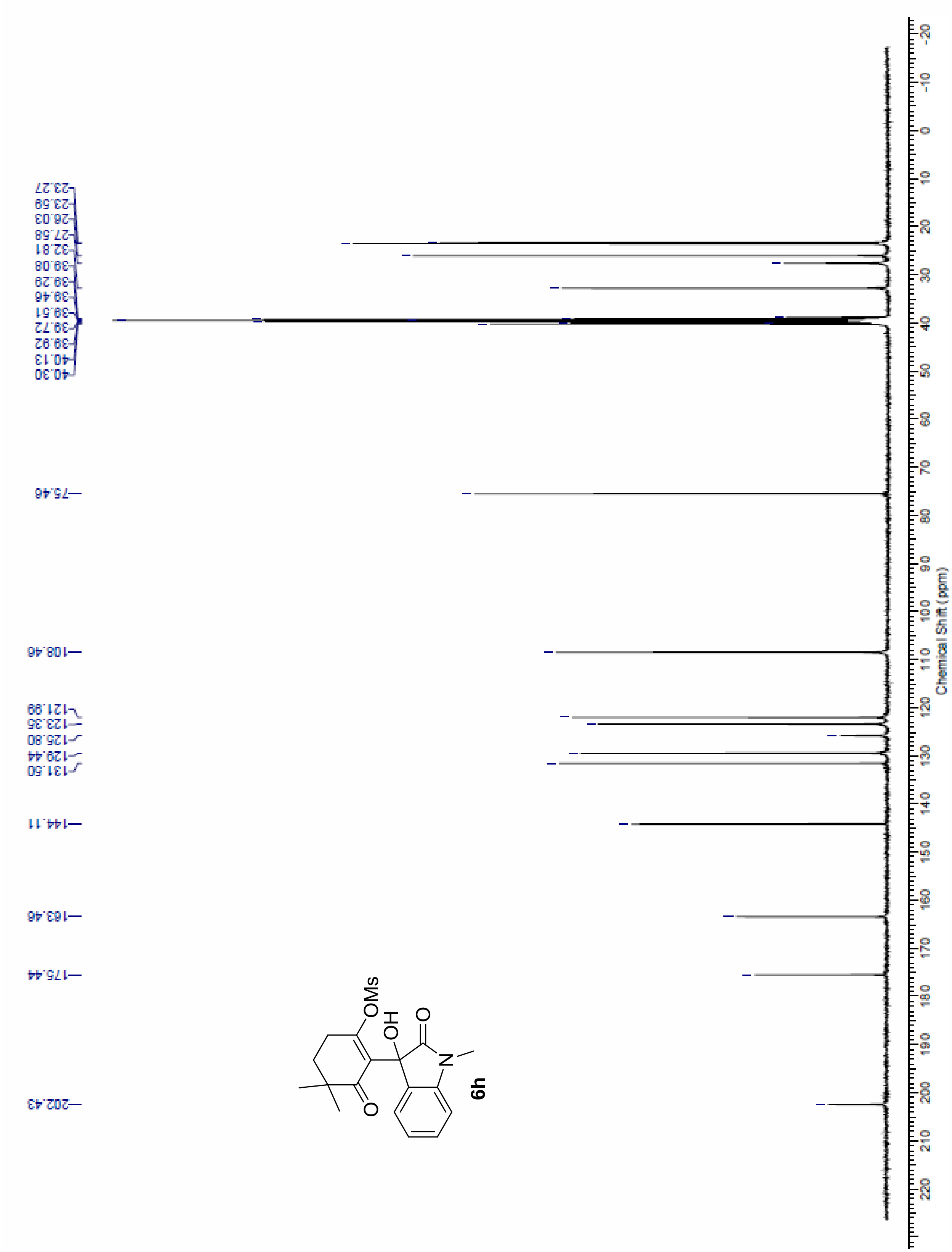

23.27
23.59
26.03
27.58
32.81
39.08
39.29
39.46
39.51
39.72
39.92
40.13
40.30
75.46
108.46
121.99
123.35
125.80
129.44
131.50
144.11
163.46
175.44
202.43
OMs
OH
O
N
6h
Chemical Shift (ppm)

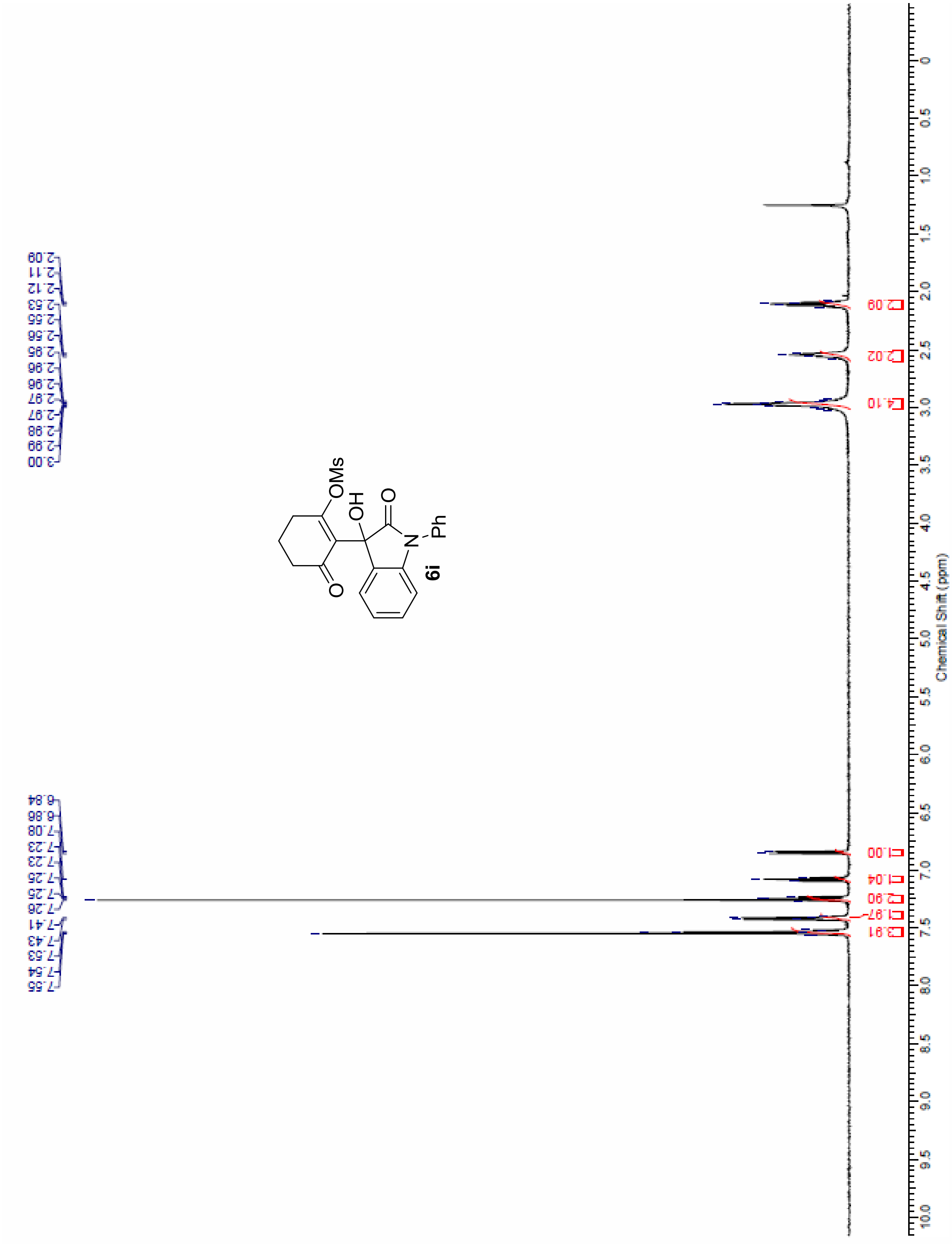
OMs
OH
O
N
Ph
6i
Chemical Shift (ppm)

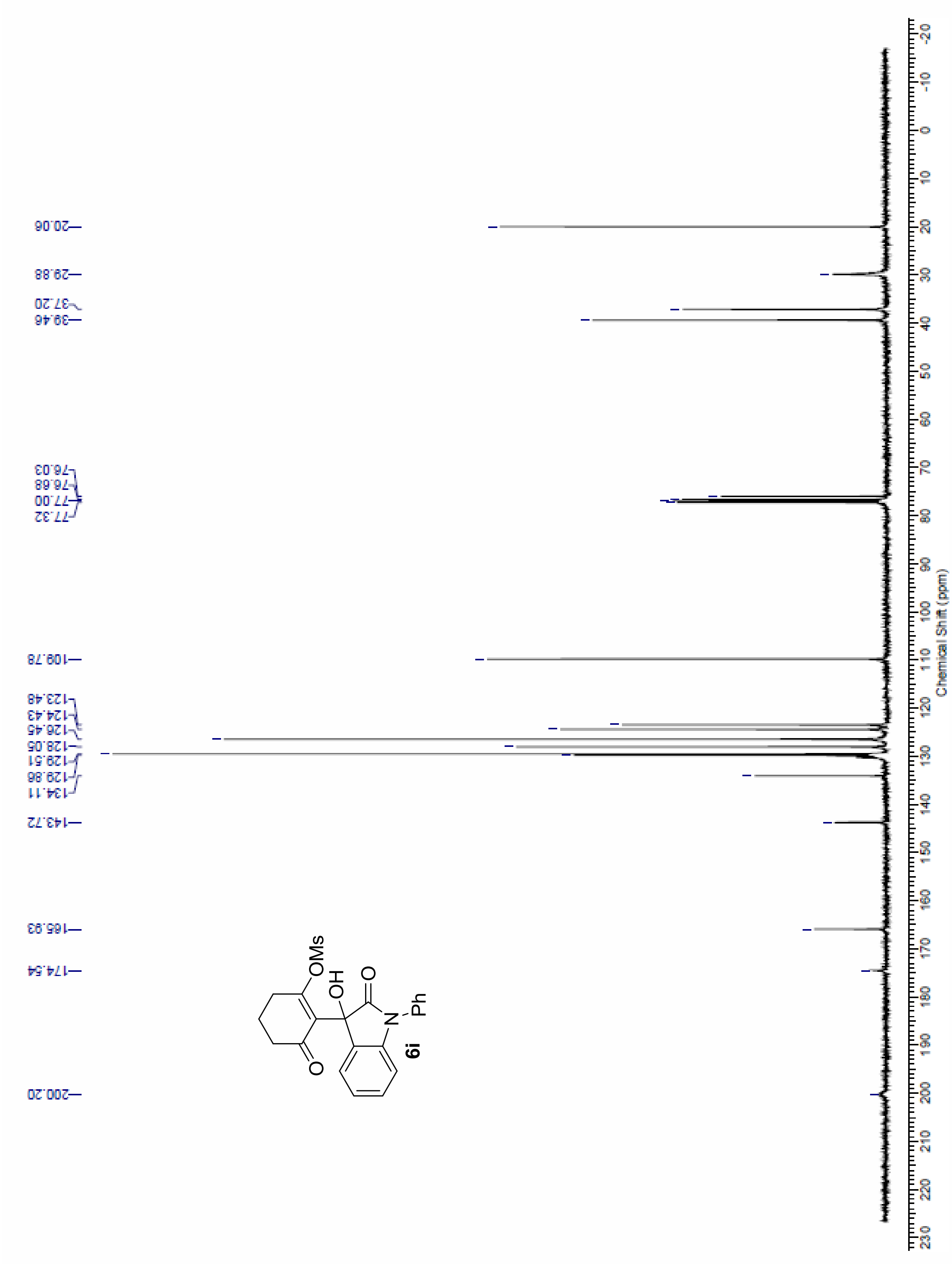

20.06
29.88
37.20
39.46
76.03
76.68
77.00
77.32
109.78
123.48
124.43
126.45
128.05
129.51
129.86
134.11
143.72
165.93
174.54
200.20
OMs
OH
O
N
Ph
6i
Chemical Shift (ppm)

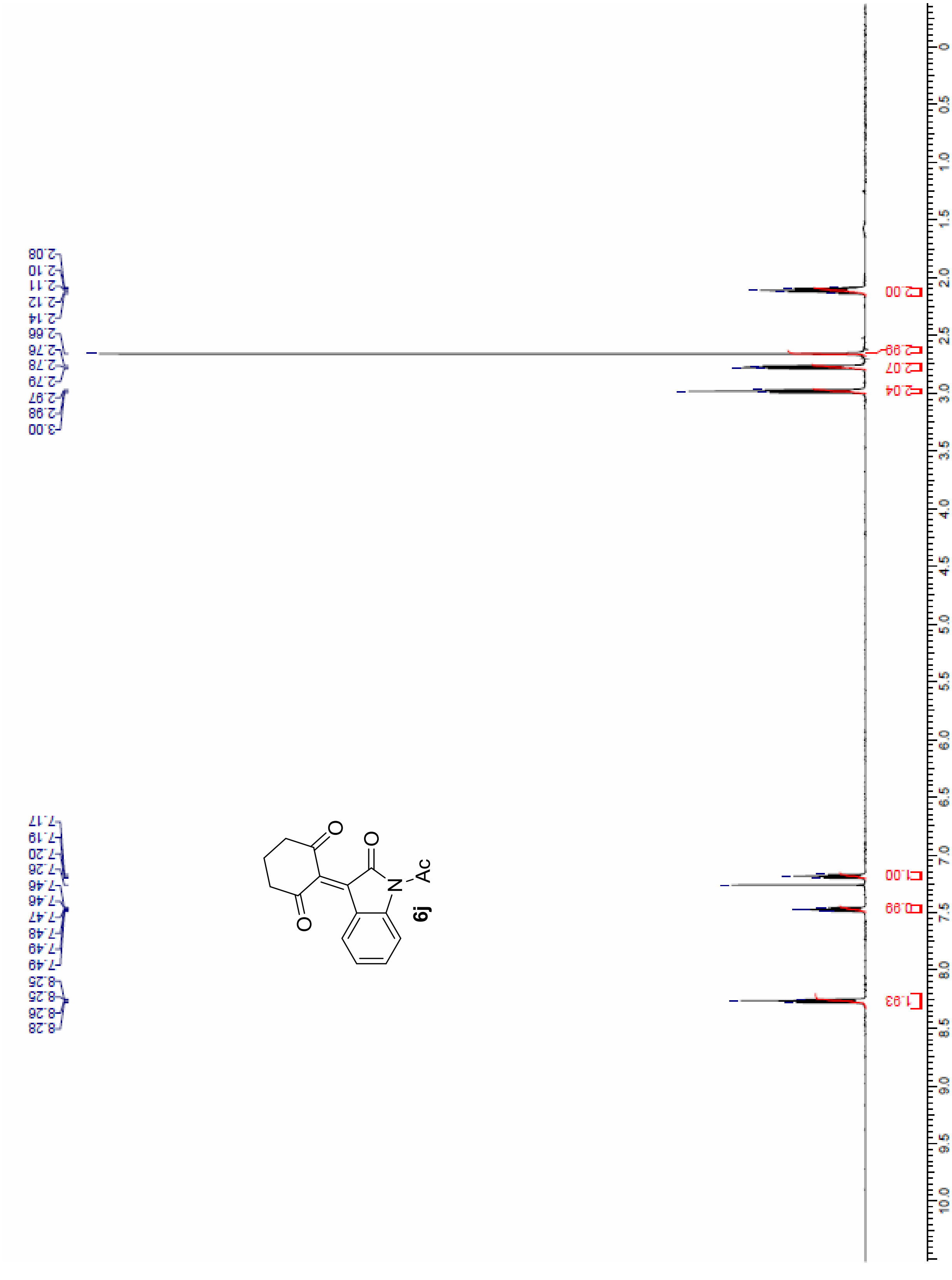
2.08
2.10
2.11
2.12
2.14
2.66
2.76
2.78
2.79
2.97
2.98
3.00
7.17
7.19
7.20
7.26
7.46
7.46
7.47
7.48
7.49
7.49
8.25
8.25
8.26
8.28
O
O
O
N
Ac
6j
2.00
2.99
2.07
2.04
1.00
0.99
1.83
Chemical Shift (ppm)

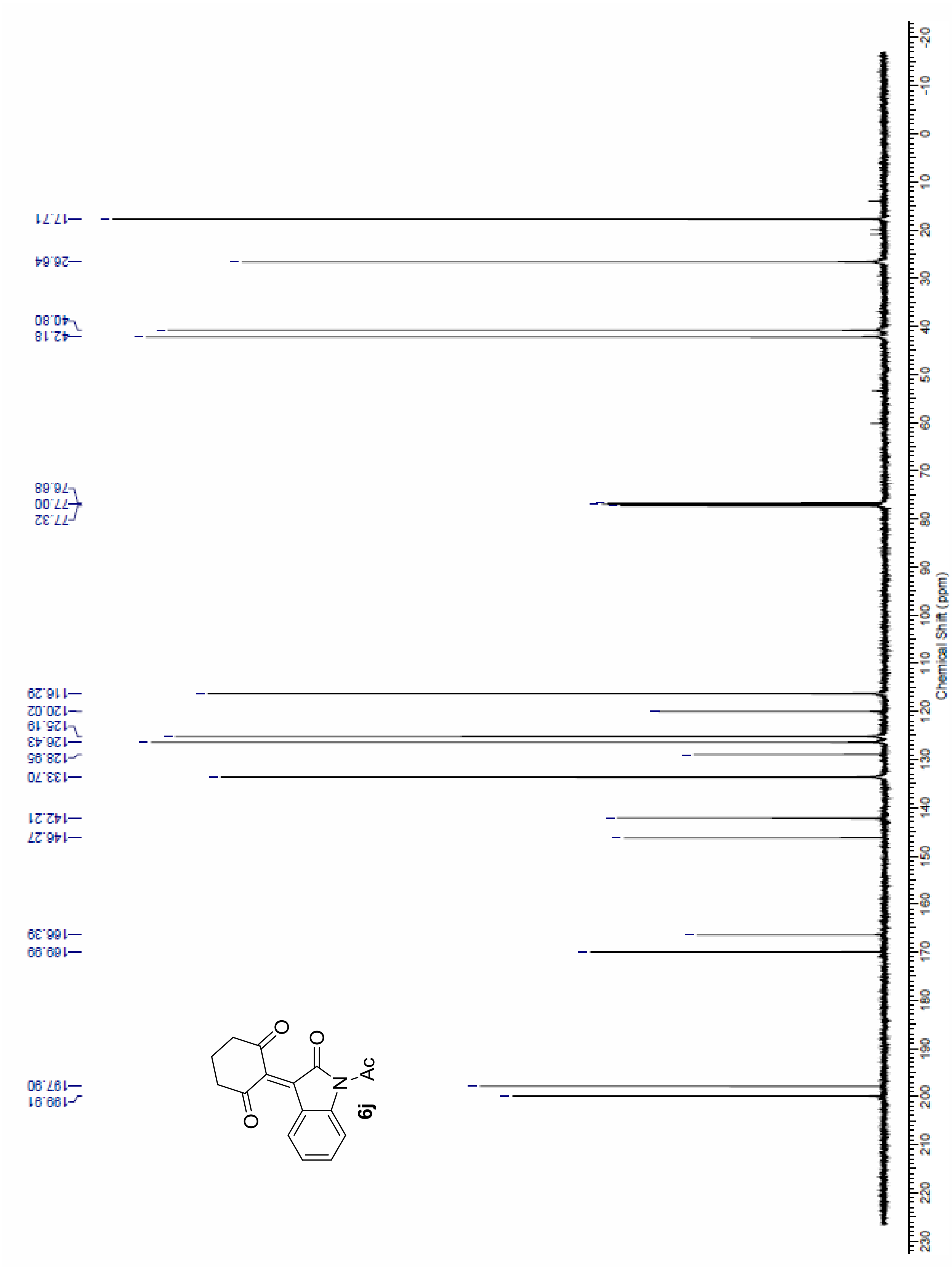

17.71
26.64
40.80
42.18
76.68
77.00
77.32
116.29
120.02
125.19
126.43
128.95
133.70
142.21
146.27
166.39
169.99
197.90
199.91
6j
Ac
N
O
Chemical Shift (ppm)

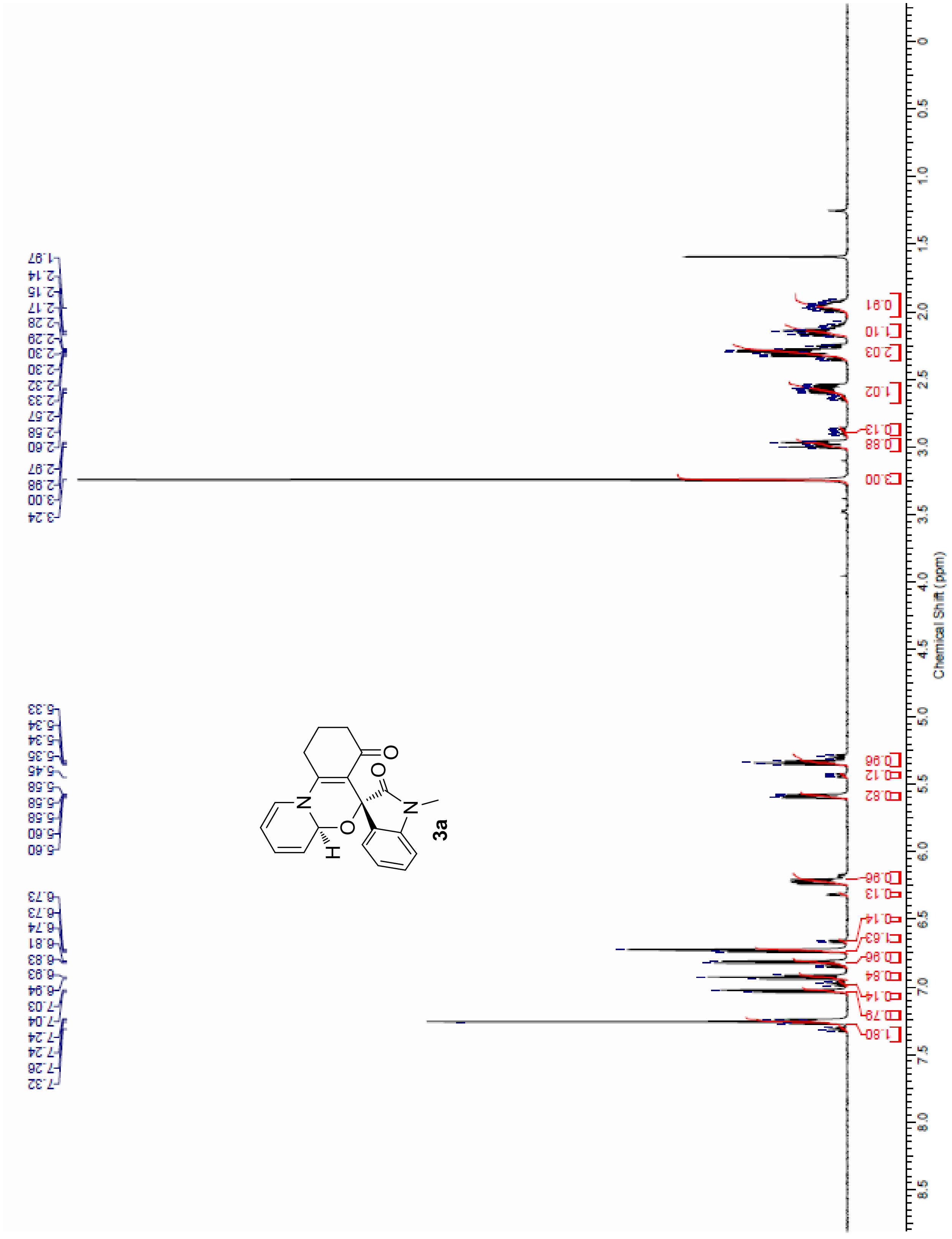
Chemical Shift (ppm)
3a

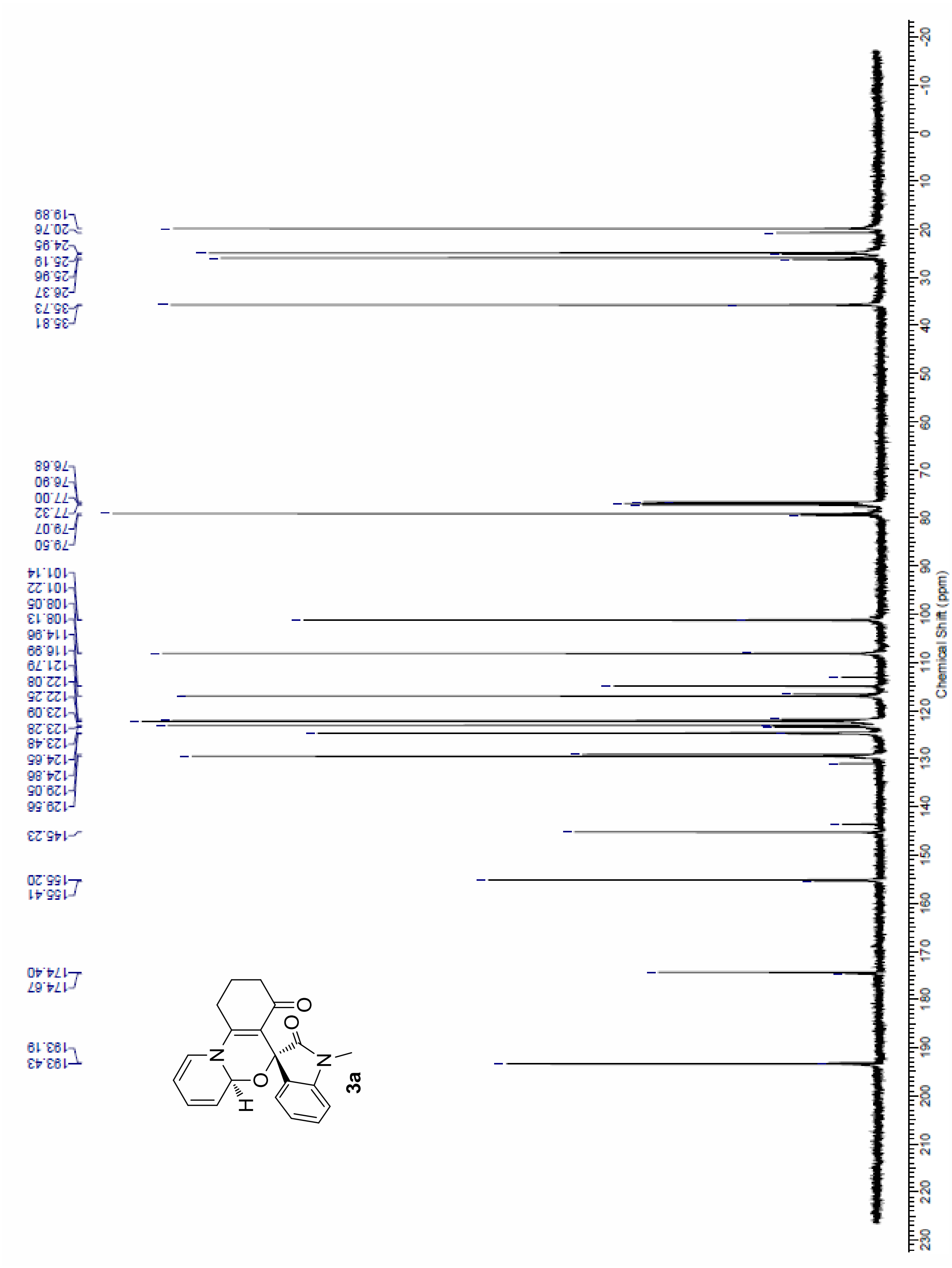

193.43
193.19
174.67
174.40
155.41
155.20
145.23
129.56
129.05
124.86
124.65
123.48
123.28
123.09
122.25
122.08
121.79
116.99
114.96
108.13
108.05
101.22
101.14
79.50
79.07
77.32
77.00
76.90
76.68
35.81
35.73
26.37
25.96
25.19
24.95
20.76
19.89
Chemical Shift (ppm)
3a

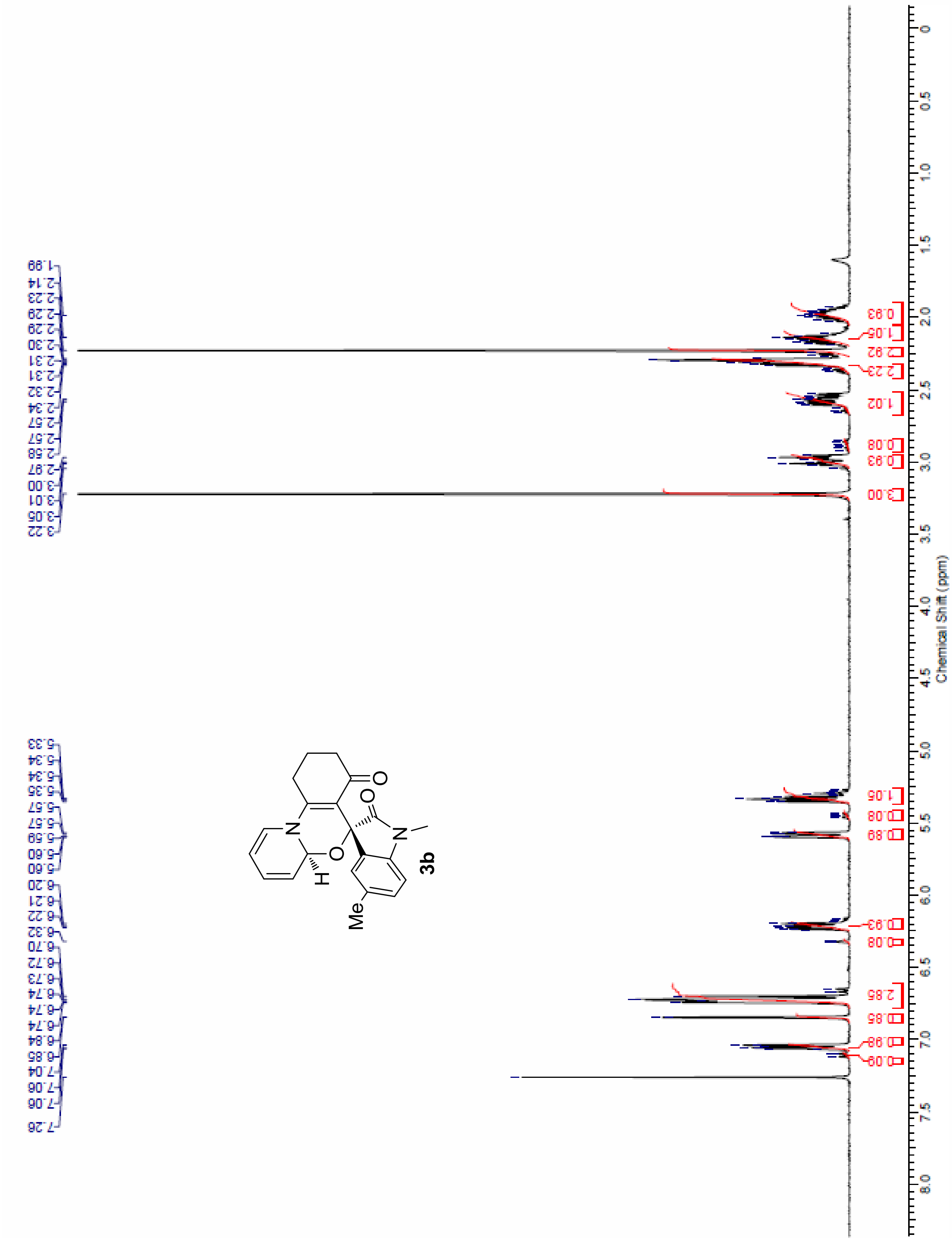
1.99
2.14
2.23
2.29
2.29
2.30
2.31
2.31
2.32
2.34
2.57
2.57
2.58
2.97
3.00
3.01
3.05
3.22
5.33
5.34
5.34
5.36
5.57
5.57
5.59
5.60
5.60
6.20
6.21
6.22
6.32
6.70
6.72
6.73
6.74
6.74
6.74
6.84
6.85
7.04
7.06
7.06
7.26
3b
Chemical Shift (ppm)

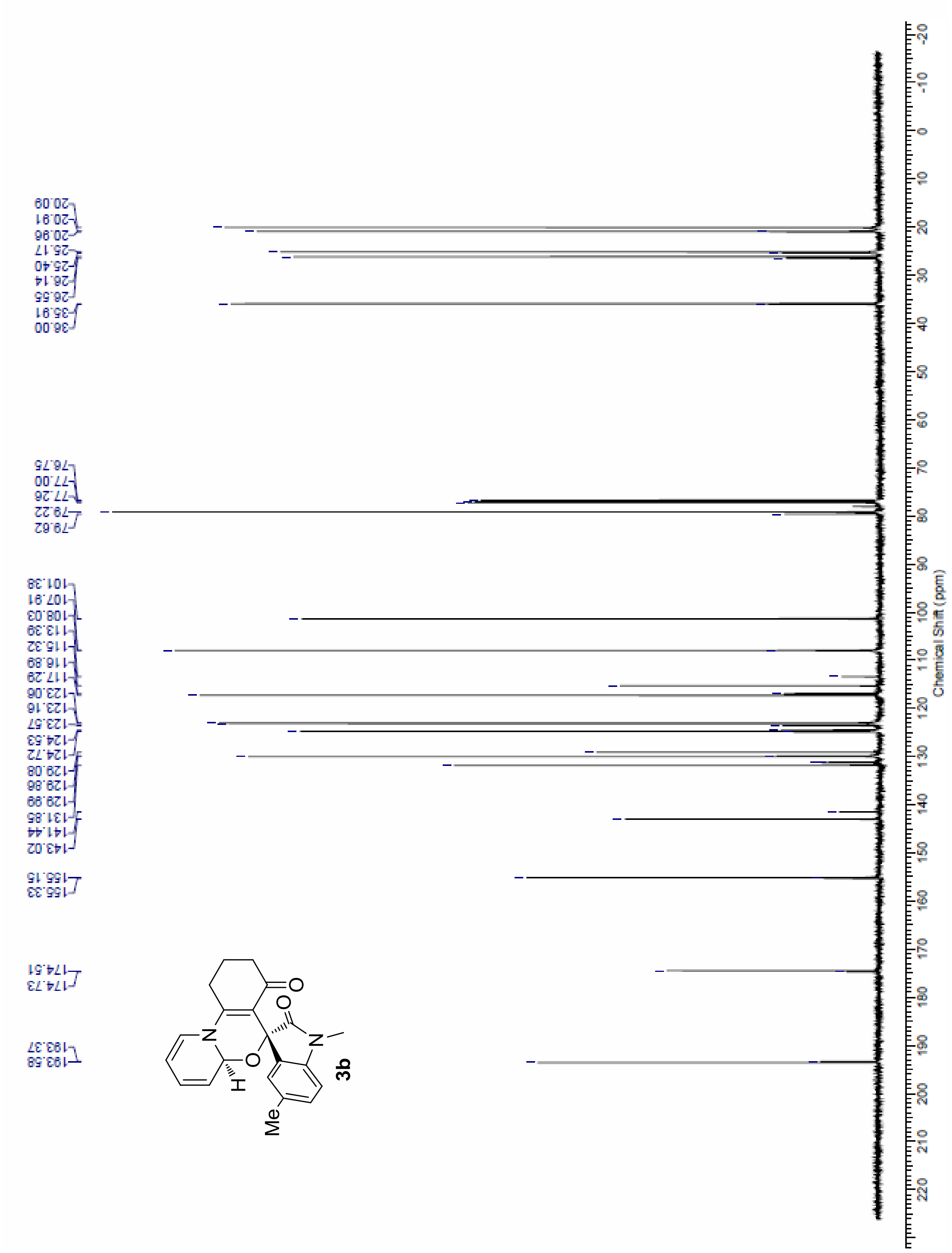
193.58
193.37
174.73
174.51
155.33
155.15
143.02
141.44
131.85
129.99
129.86
129.08
124.72
124.53
123.57
123.16
123.06
117.29
116.89
115.32
113.39
108.03
107.91
101.38
79.62
79.22
77.26
77.00
76.75
36.00
35.91
26.55
26.14
25.40
25.17
20.96
20.91
20.09
Me
3b
Chemical Shift (ppm)

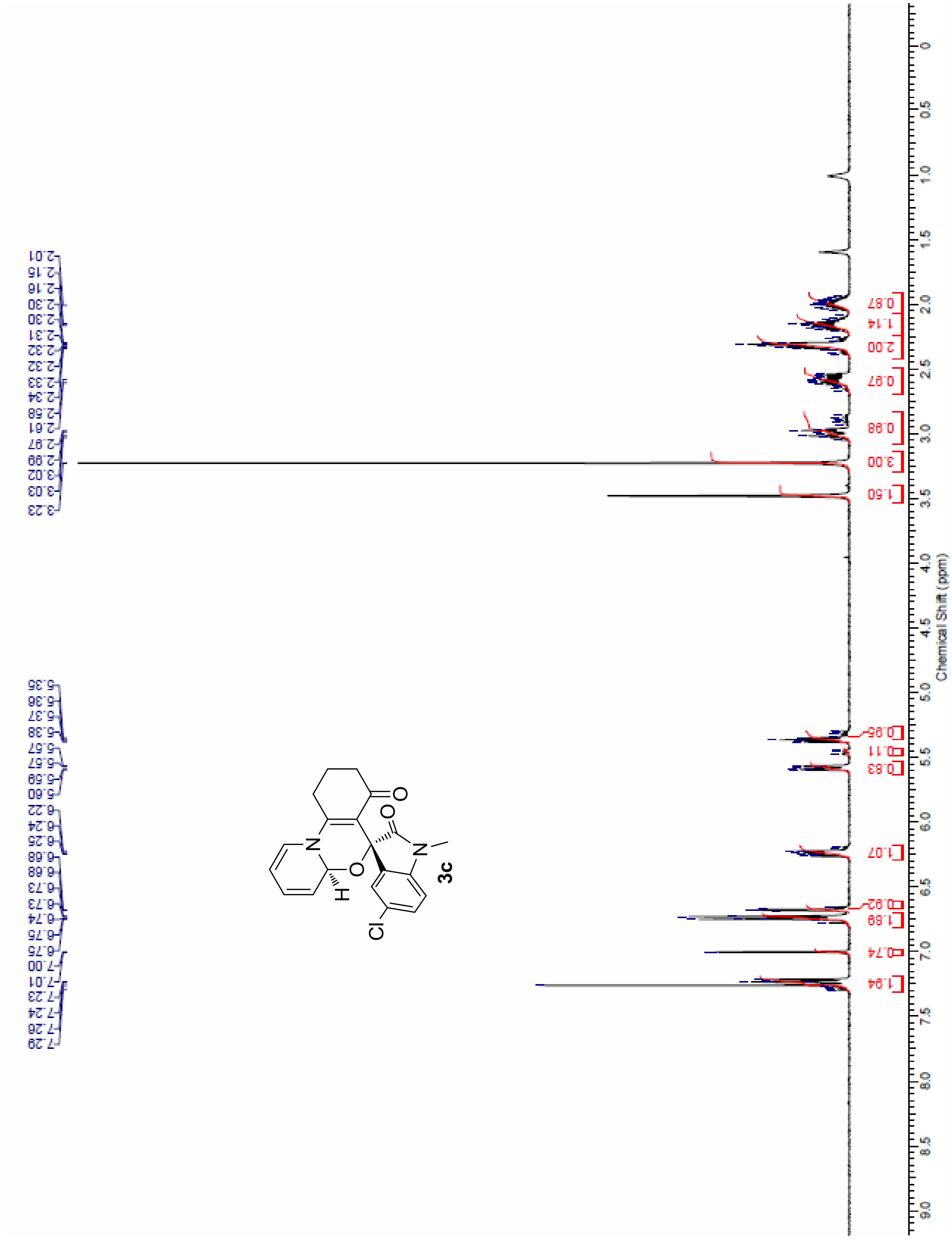
Chemical Shift (ppm)
3c

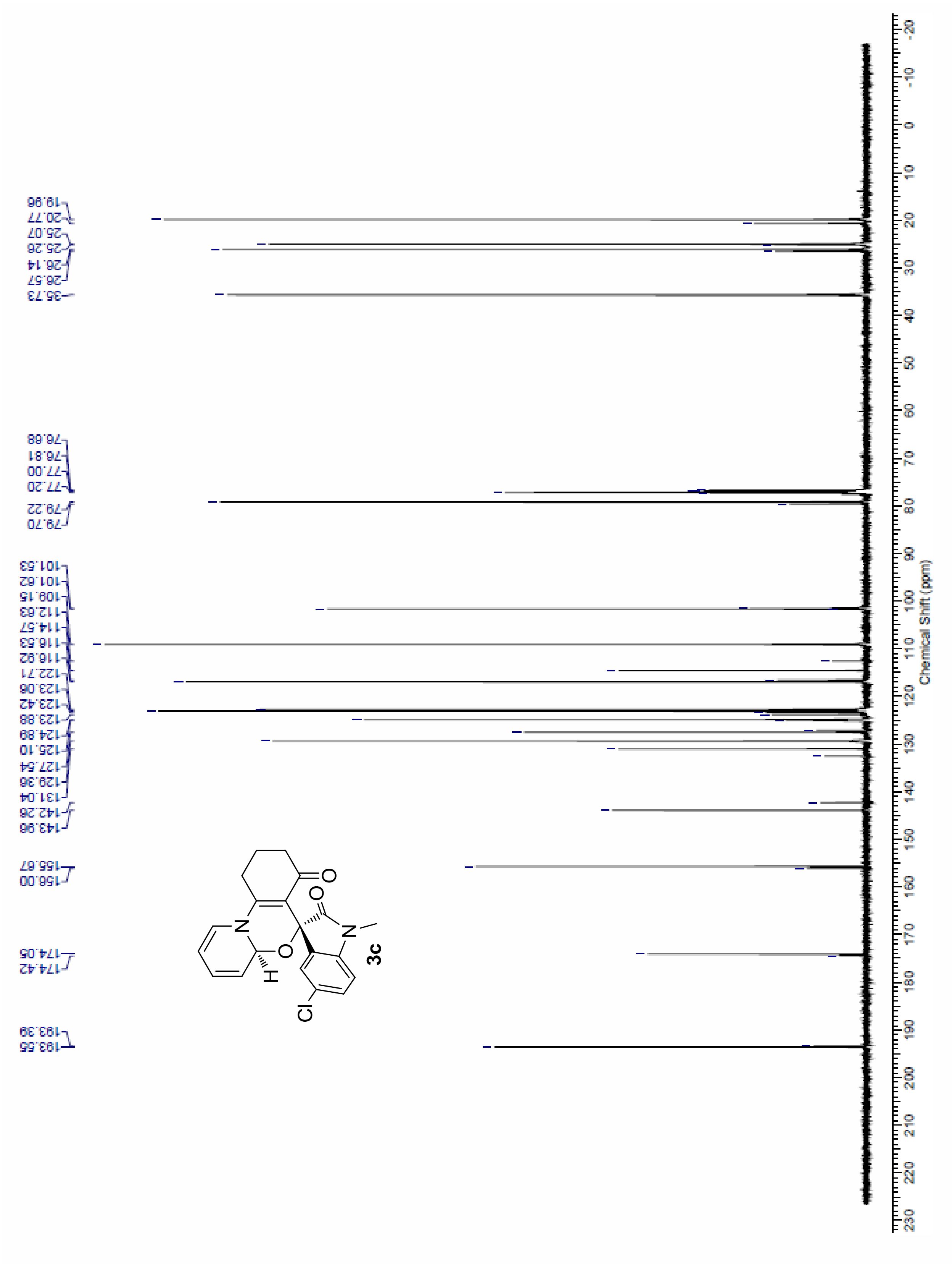
19.96
20.77
25.07
25.28
26.14
26.57
35.73
76.68
76.81
77.00
77.20
79.22
79.70
101.53
101.62
109.15
112.63
114.57
116.53
116.92
122.71
123.06
123.42
123.88
124.89
125.10
127.54
129.36
131.04
142.26
143.96
155.67
156.00
174.05
174.42
193.39
193.55
3c
Cl
-20 -10 0 10 20 30 40 50 60 70 80 90 100 110 120 130 140 150 160 170 180 190 200 210 220 230
Chemical Shift (ppm)

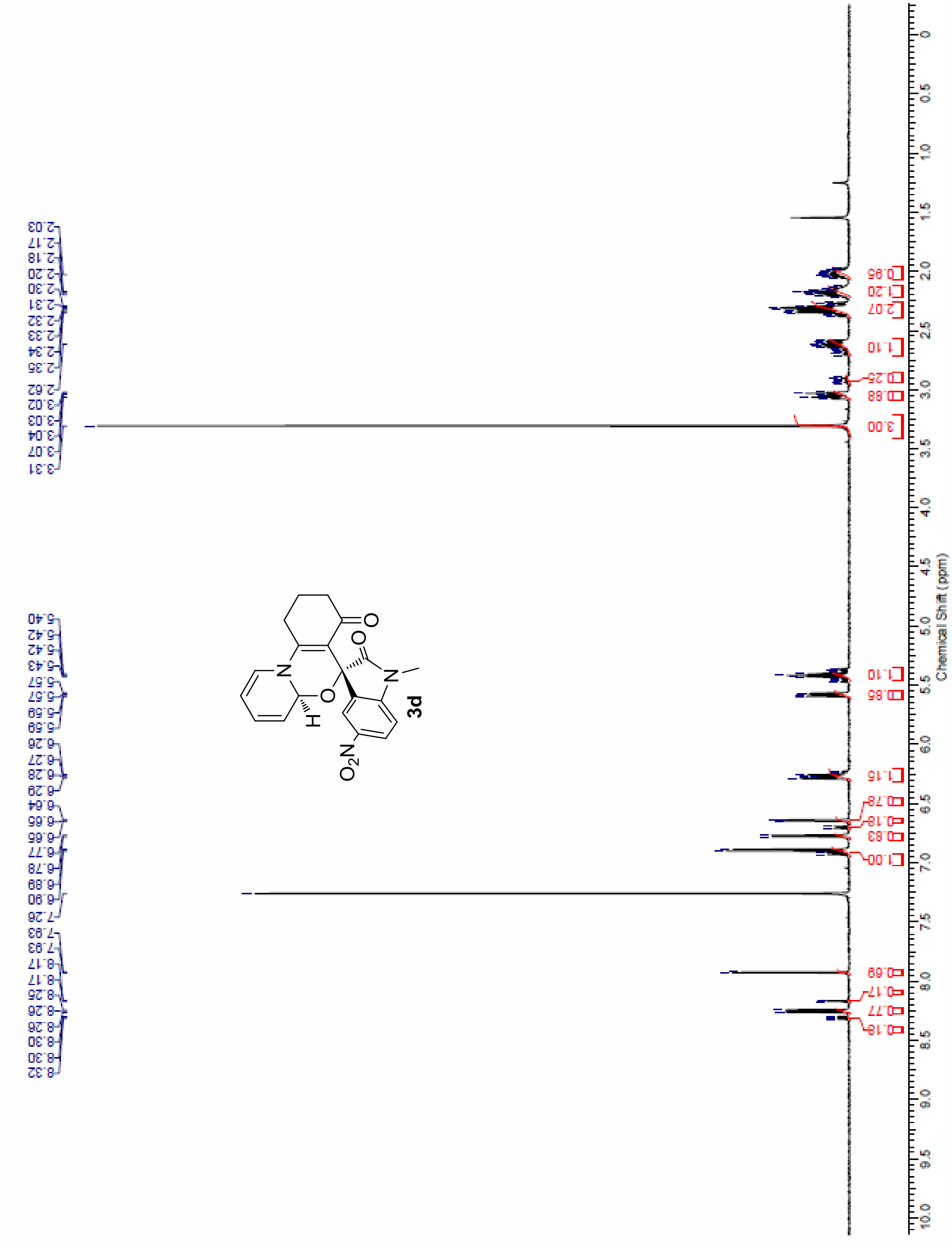

2.03
2.17
2.18
2.20
2.30
2.31
2.32
2.33
2.34
2.35
2.62
3.02
3.03
3.04
3.07
3.31
5.40
5.42
5.42
5.43
5.57
5.57
5.59
5.59
6.26
6.27
6.28
6.29
6.64
6.65
6.65
6.77
6.78
6.89
6.90
7.26
7.93
7.93
8.17
8.17
8.25
8.26
8.26
8.30
8.30
8.32
3d
0.95
1.20
2.07
1.10
0.25
0.88
3.00
1.10
0.85
1.15
0.78
0.18
0.83
1.00
0.69
0.17
0.77
0.18
Chemical Shift (ppm)

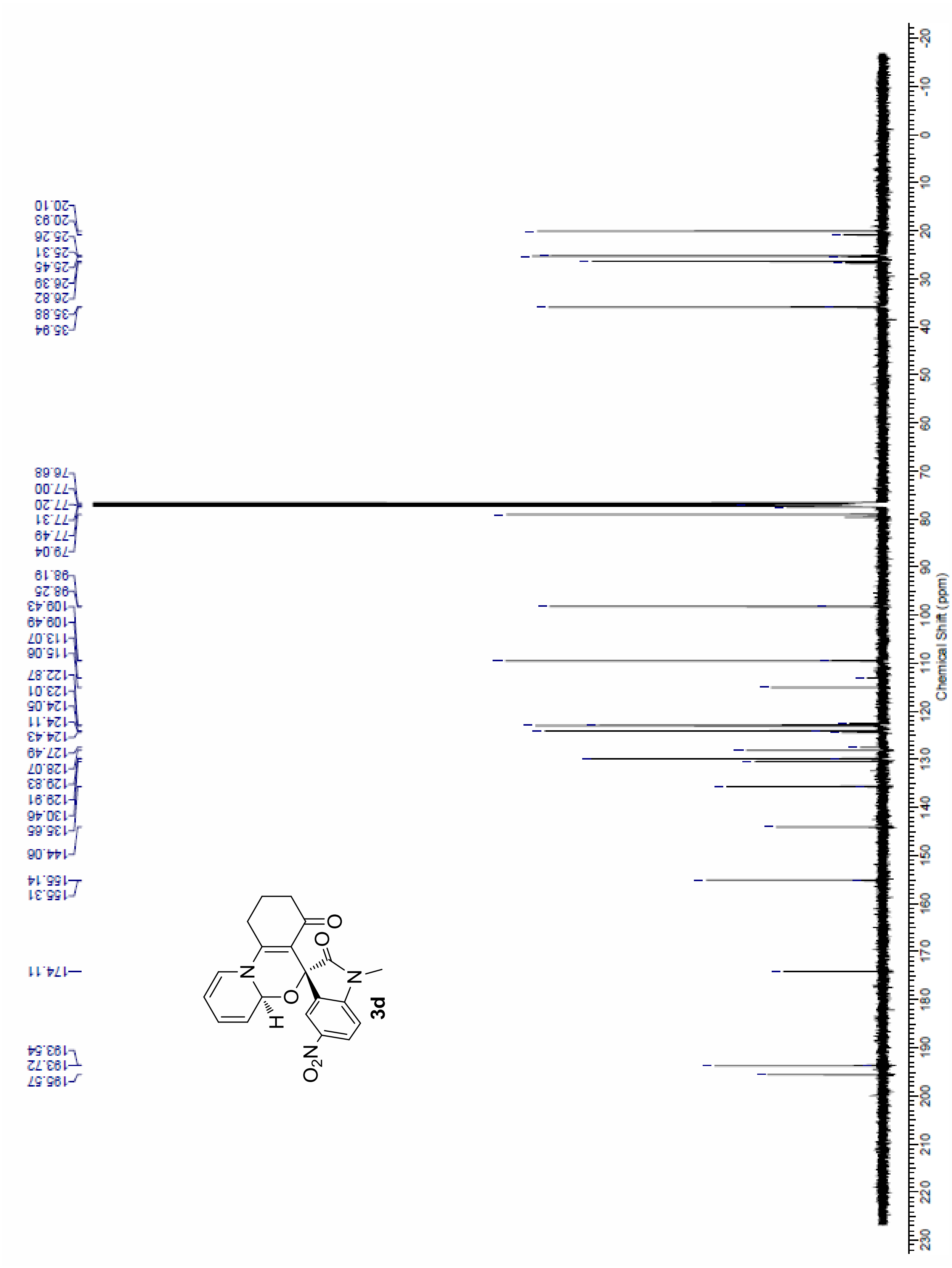
20.10
20.93
25.26
25.31
25.45
26.39
26.82
35.88
35.94
76.68
77.00
77.20
77.31
77.49
79.04
98.19
98.25
109.43
109.49
113.07
115.06
122.87
123.01
124.05
124.11
124.43
127.49
128.07
129.83
129.91
130.46
135.65
144.06
155.14
155.31
174.11
193.54
193.72
195.57
3d
Chemical Shift (ppm)

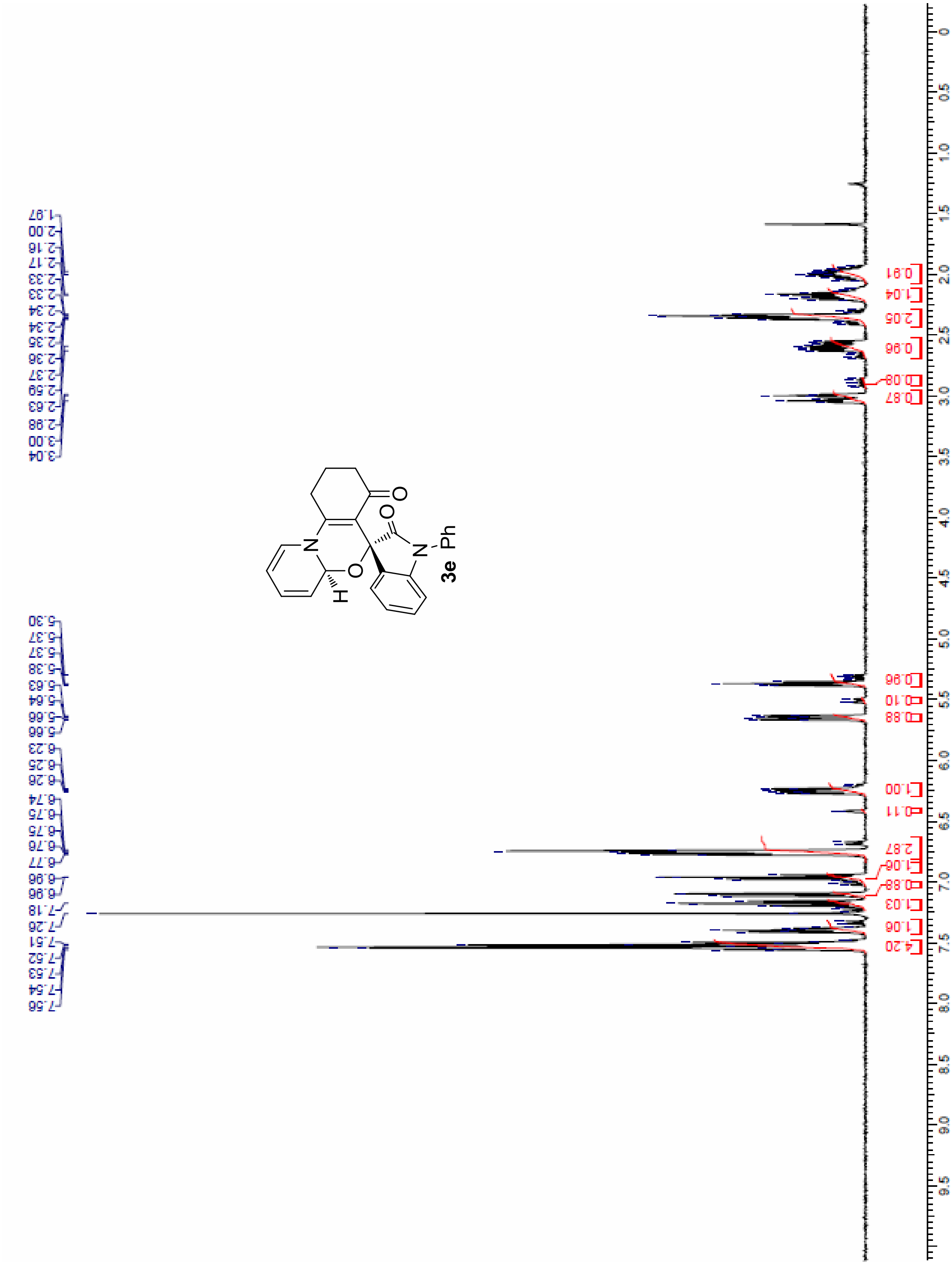

3e
Chemical Shift (ppm)

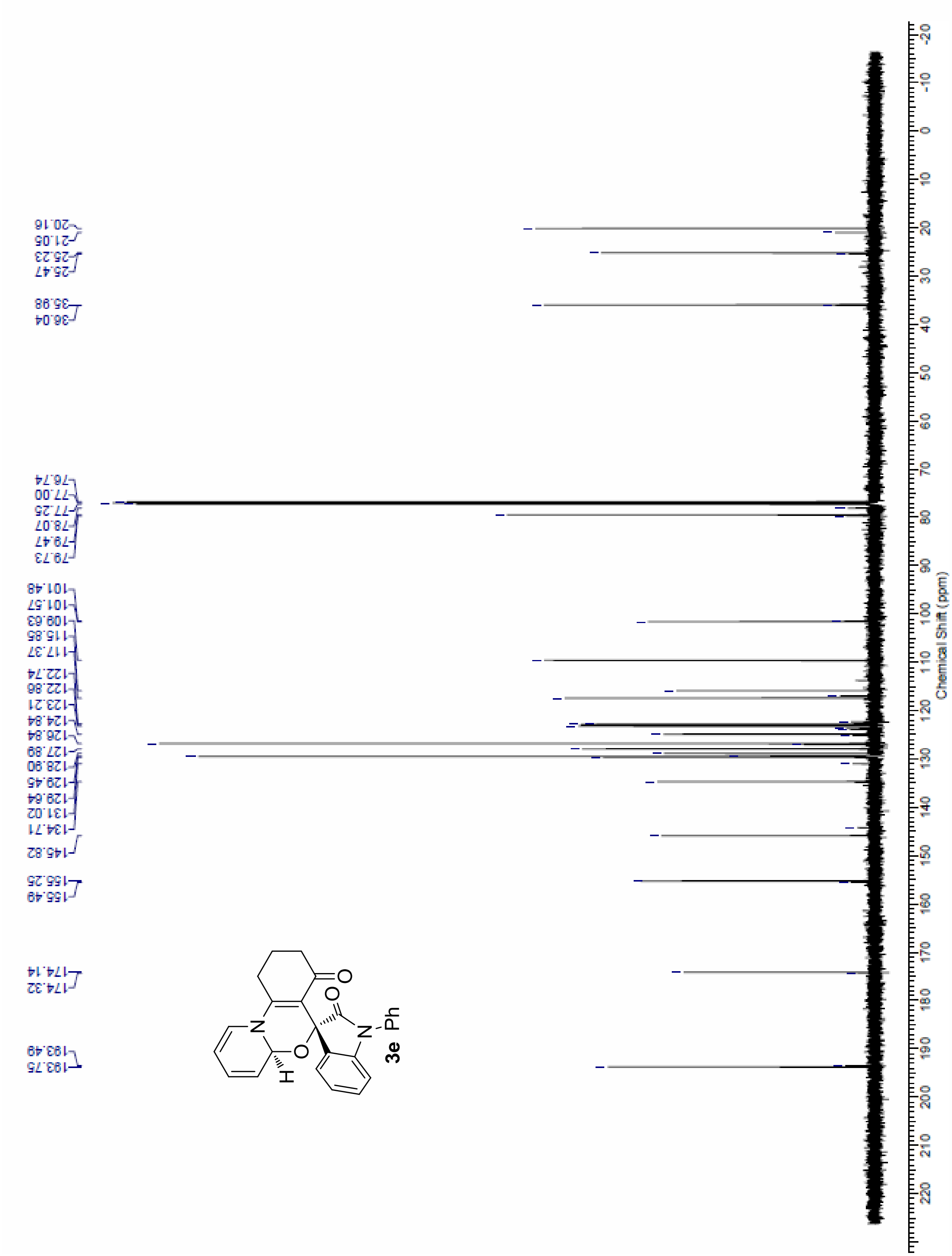
20.16
21.05
25.23
25.47
35.98
36.04
76.74
77.00
77.25
78.07
79.47
79.73
101.48
101.57
109.63
115.85
117.37
122.74
122.86
123.21
124.84
126.84
127.89
128.90
129.45
129.64
131.02
134.71
145.82
155.25
155.49
174.14
174.32
193.49
193.75
3e
Ph
Chemical Shift (ppm)

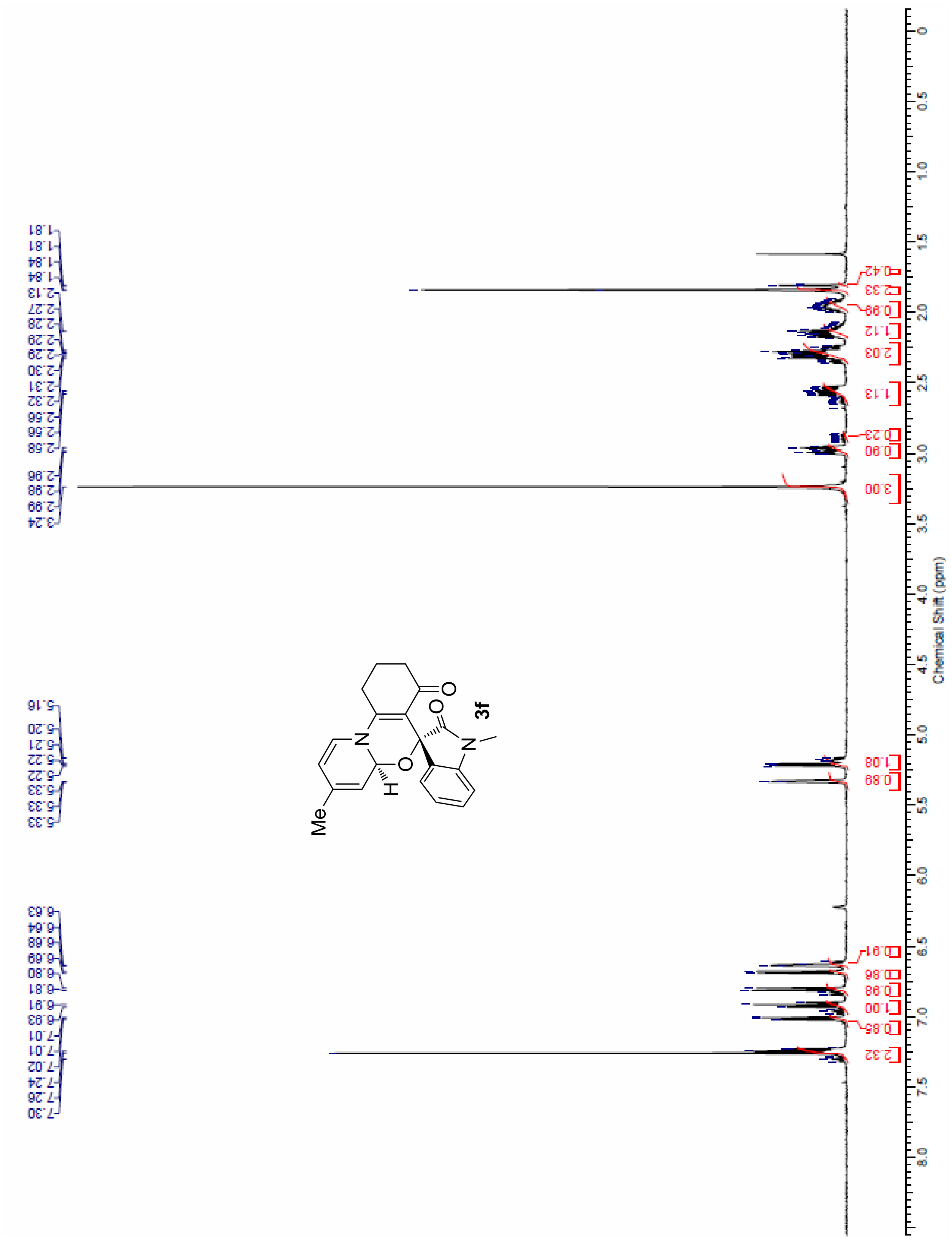
1.81
1.81
1.84
1.84
2.13
2.27
2.28
2.29
2.29
2.30
2.31
2.32
2.56
2.56
2.58
2.96
2.98
2.99
3.24
5.16
5.20
5.21
5.22
5.22
5.33
5.33
5.33
6.63
6.64
6.68
6.69
6.80
6.81
6.91
6.93
7.01
7.01
7.02
7.24
7.26
7.30
Me
N
O
H
O
N
O
3f
0.42
2.33
0.99
1.12
2.03
1.13
0.23
0.90
3.00
1.08
0.89
0.91
0.86
0.98
1.00
0.85
2.32
Chemical Shift (ppm)

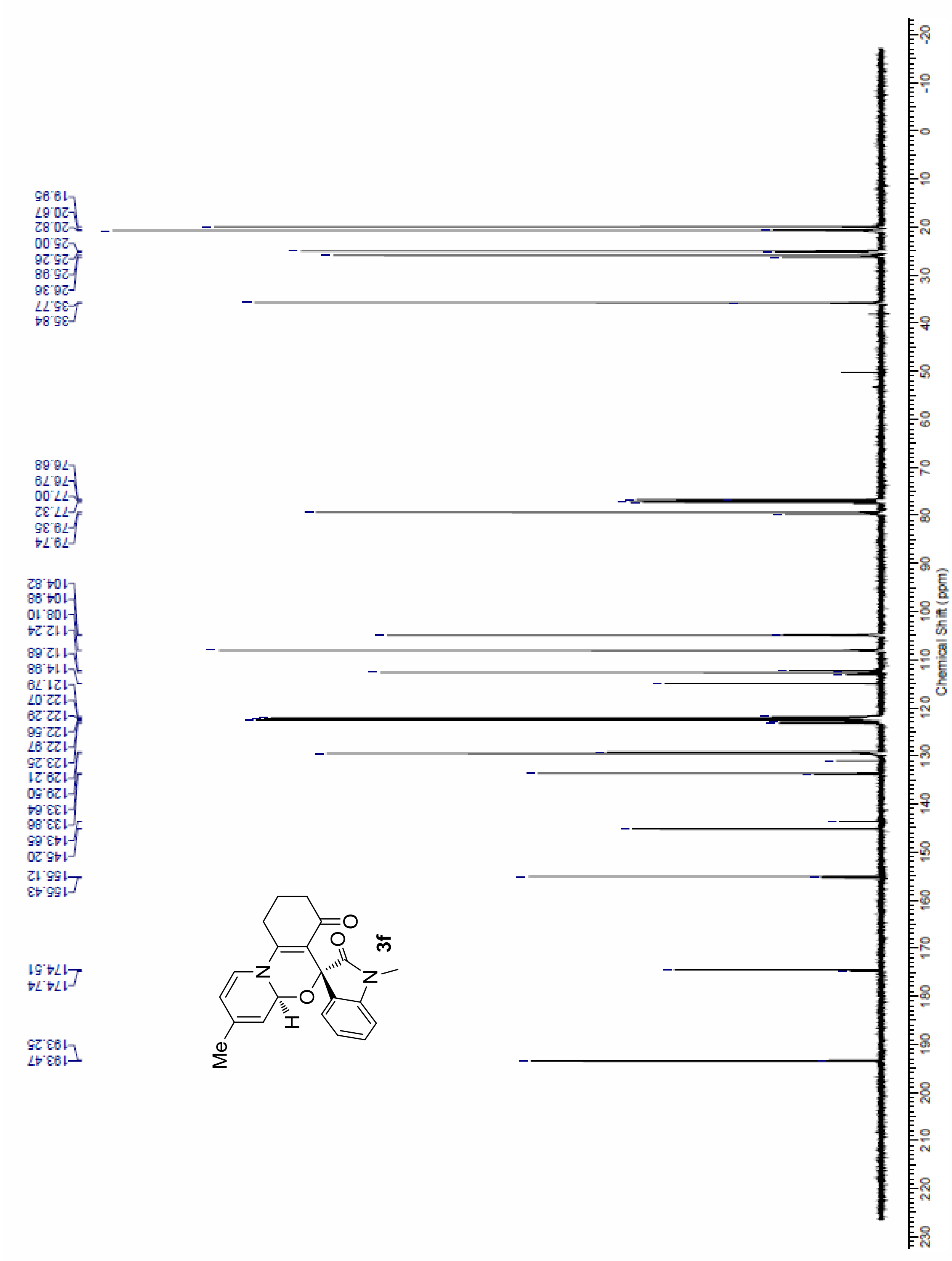
19.95
20.67
20.82
25.00
25.26
25.98
26.36
35.77
35.84
76.68
76.79
77.00
77.32
79.35
79.74
104.82
104.98
108.10
112.24
112.68
114.98
121.79
122.07
122.29
122.56
122.97
123.25
129.21
129.50
133.64
133.86
143.65
145.20
155.12
155.43
174.51
174.74
193.25
193.47
Me
N
O
H
N
3f
Chemical Shift (ppm)

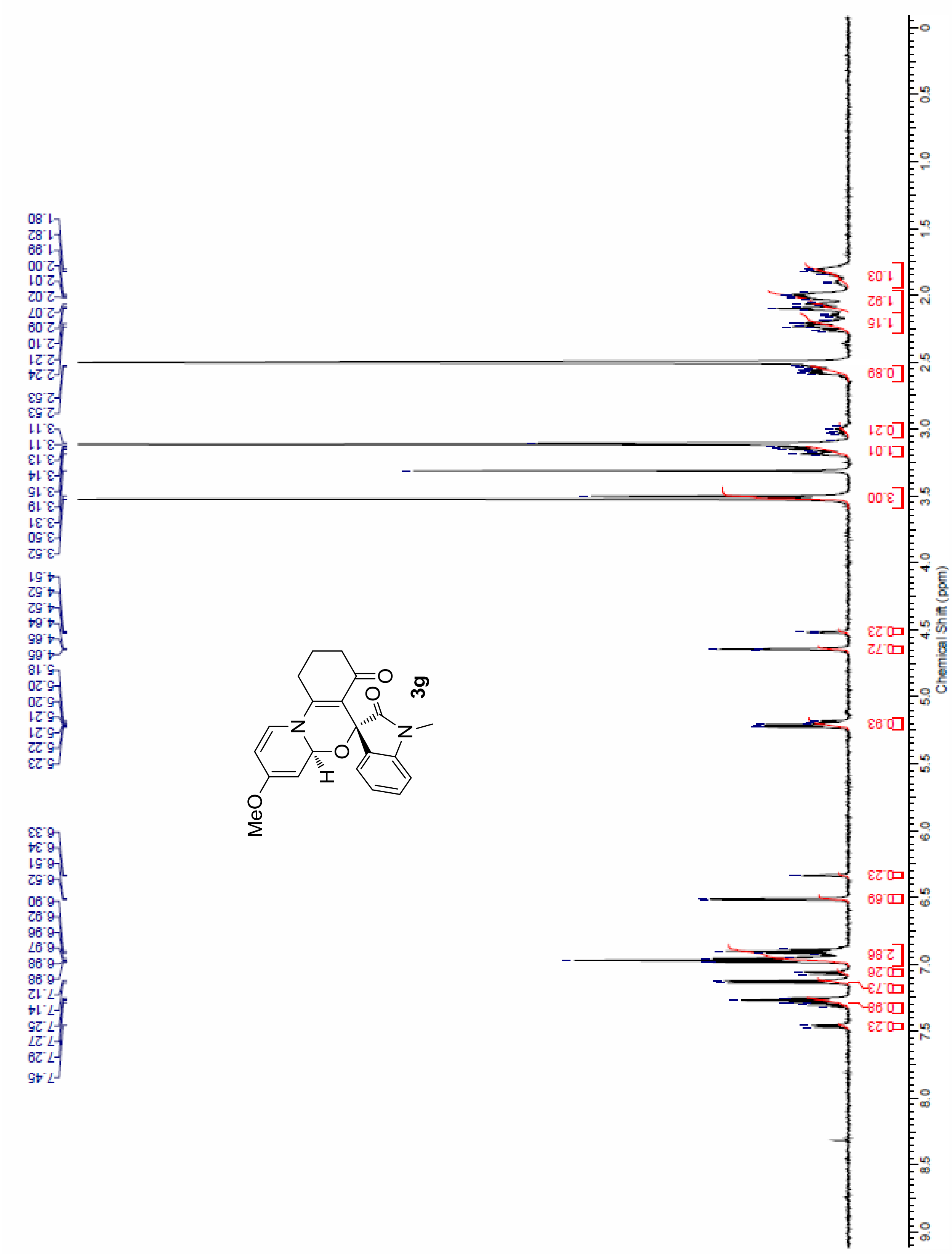
7.45
7.29
7.27
7.25
7.14
7.12
6.98
6.98
6.97
6.96
6.92
6.90
6.52
6.51
6.34
6.33
5.23
5.22
5.21
5.21
5.20
5.18
4.66
4.66
4.64
4.62
4.52
4.51
3.52
3.50
3.31
3.19
3.16
3.14
3.13
3.11
3.11
2.53
2.53
2.24
2.21
2.10
2.09
2.07
2.02
2.01
2.00
1.99
1.82
1.80
MeO
N
O
H
O
O
N
3g
1.03
1.92
1.15
0.89
0.21
1.01
3.00
0.23
0.72
0.93
0.23
0.69
2.86
0.26
0.73
0.98
0.23
Chemical Shift (ppm)

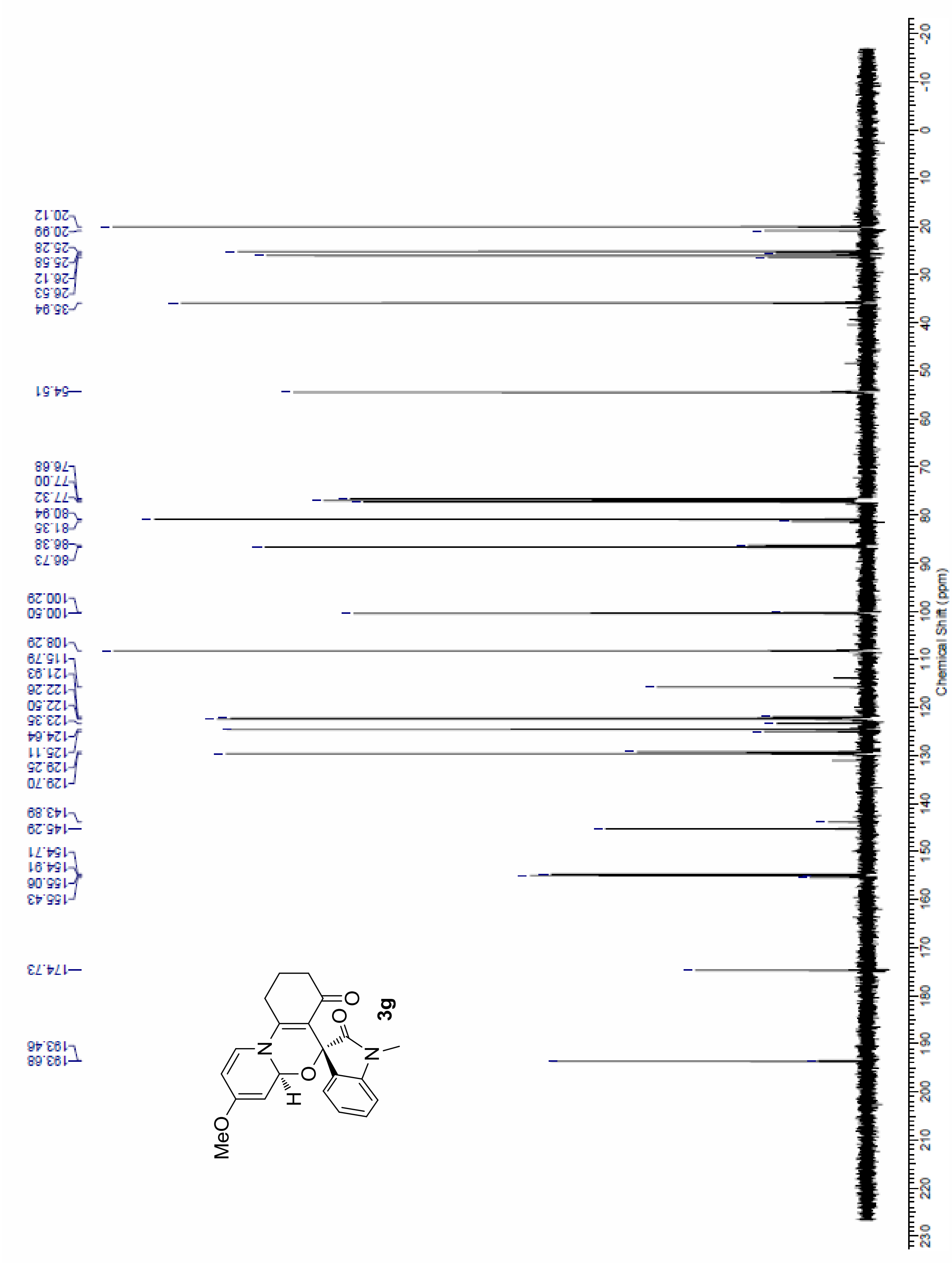
20.12
20.99
25.28
25.68
26.12
26.53
35.94
54.51
76.68
77.00
77.32
80.94
81.35
86.38
86.73
100.29
100.50
108.29
115.79
121.93
122.26
122.50
123.35
124.64
125.11
129.25
129.70
143.89
145.29
154.71
154.91
155.06
155.43
174.73
193.46
193.68
MeO
3g
Chemical Shift (ppm)

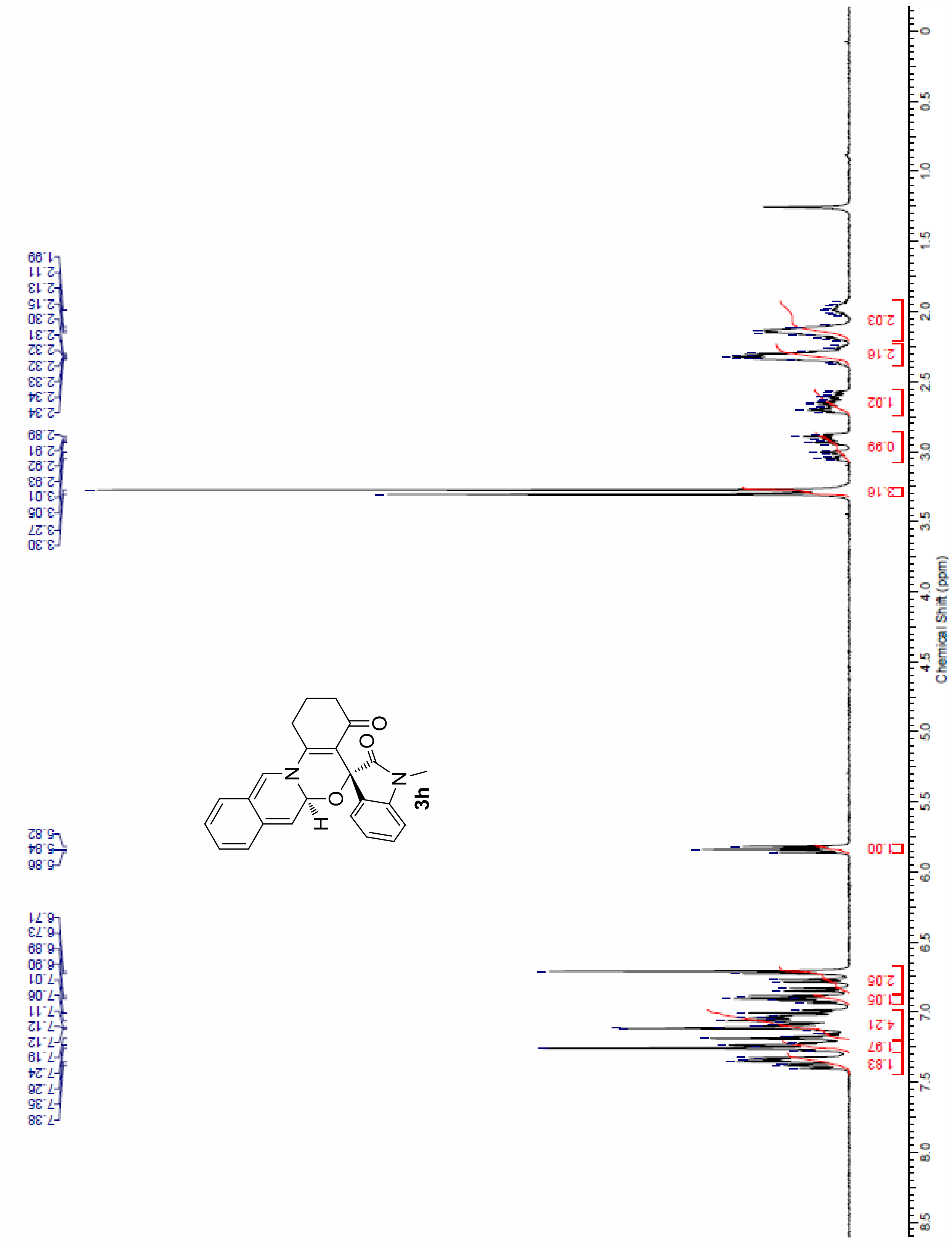
3h
Chemical Shift (ppm)

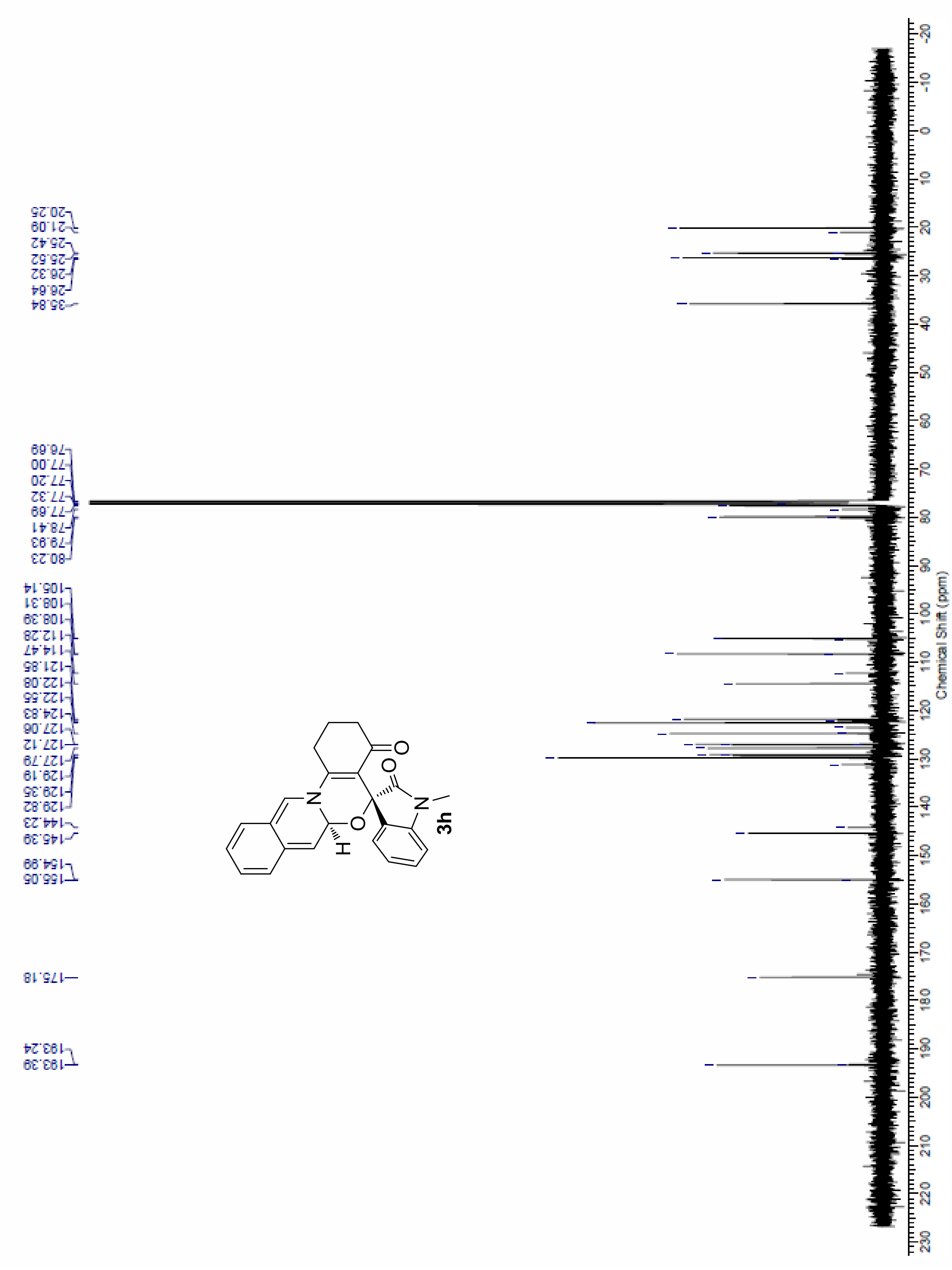
20.25
21.09
25.42
25.62
26.32
26.64
35.84
76.69
77.00
77.20
77.32
77.69
78.41
79.93
80.23
105.14
108.31
108.39
112.28
114.47
121.85
122.08
122.55
124.83
127.06
127.12
127.79
129.19
129.35
129.82
144.23
145.39
154.99
155.06
175.18
193.24
193.39
3h
Chemical Shift (ppm)

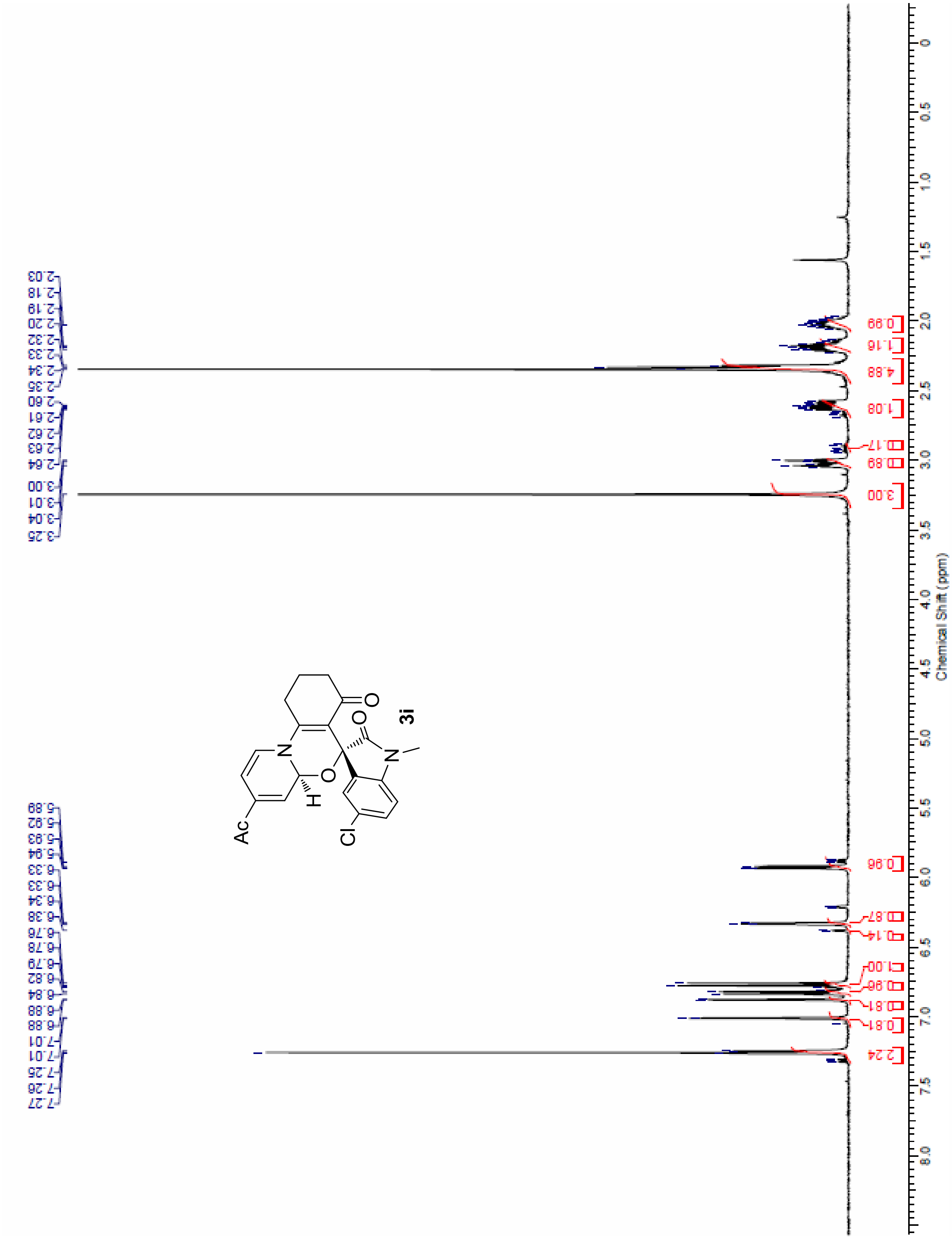

2.03
2.18
2.19
2.20
2.32
2.33
2.34
2.35
2.60
2.61
2.62
2.63
2.64
3.00
3.01
3.04
3.25
5.89
5.92
5.93
5.94
6.33
6.33
6.34
6.38
6.76
6.78
6.79
6.82
6.84
6.88
6.88
7.01
7.01
7.25
7.26
7.27
Ac
N
O
H
O
N
Cl
O
3i
0.99
1.16
4.88
1.08
0.17
0.89
3.00
0.96
0.87
0.14
1.00
0.96
0.81
0.81
2.24
Chemical Shift (ppm)

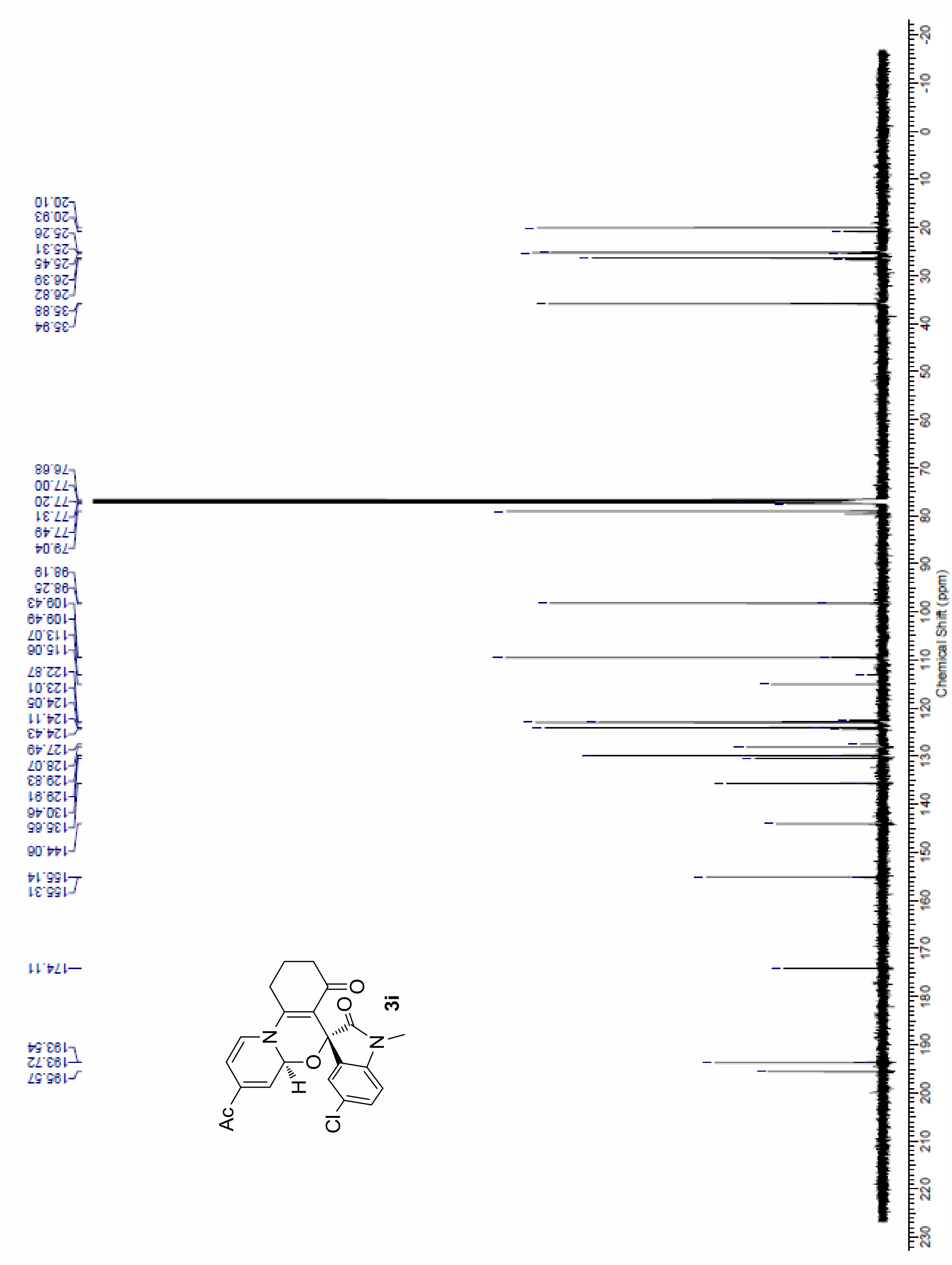
20.10
20.93
25.26
25.31
25.45
26.39
26.82
35.88
35.94
76.68
77.00
77.20
77.31
77.49
79.04
98.19
98.25
109.43
109.49
113.07
115.06
122.87
123.01
124.05
124.11
124.43
127.49
128.07
129.83
129.91
130.46
135.65
144.06
155.14
155.31
174.11
193.54
193.72
195.57
3i
Ac
Cl
Chemical Shift (ppm)

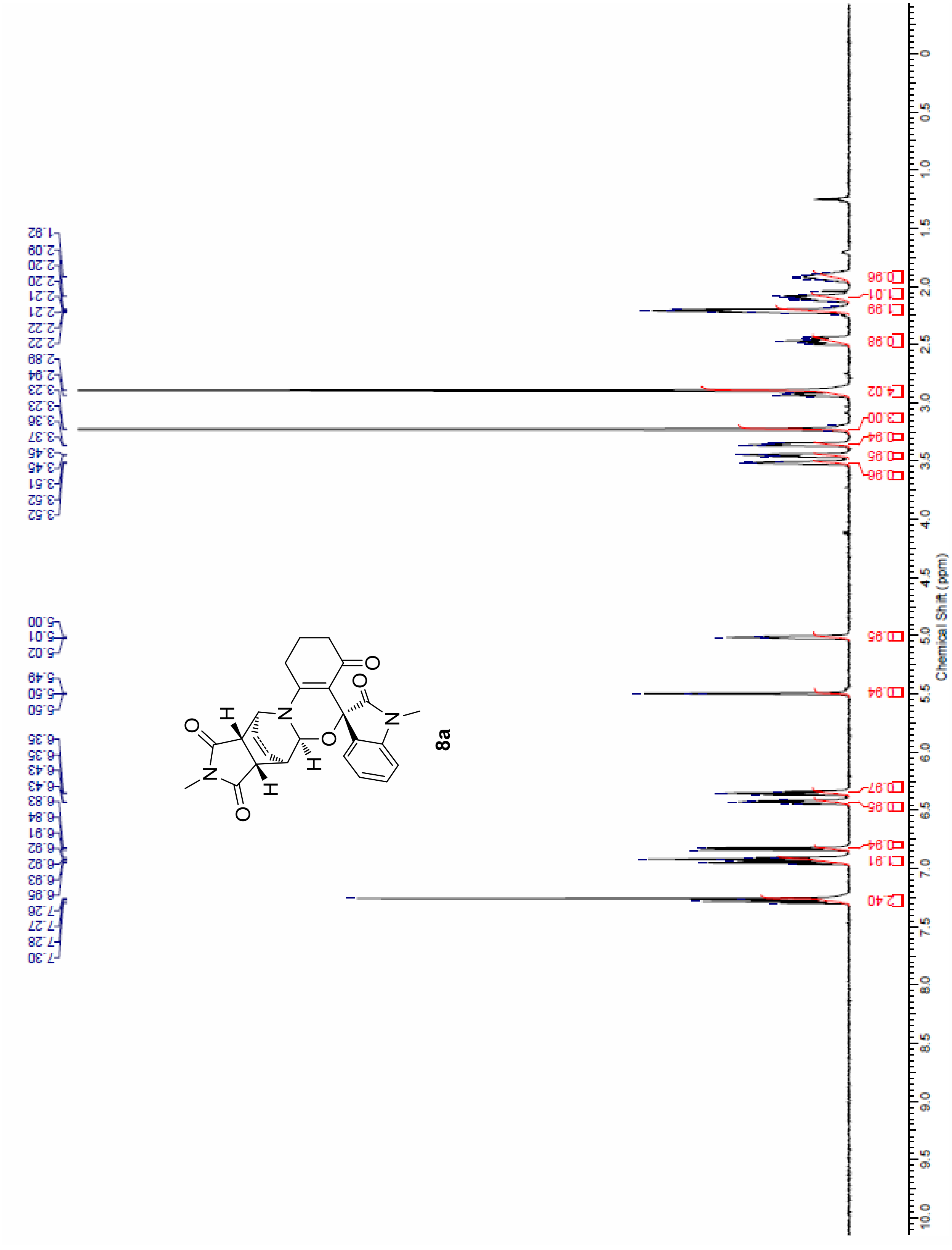
8a
Chemical Shift (ppm)

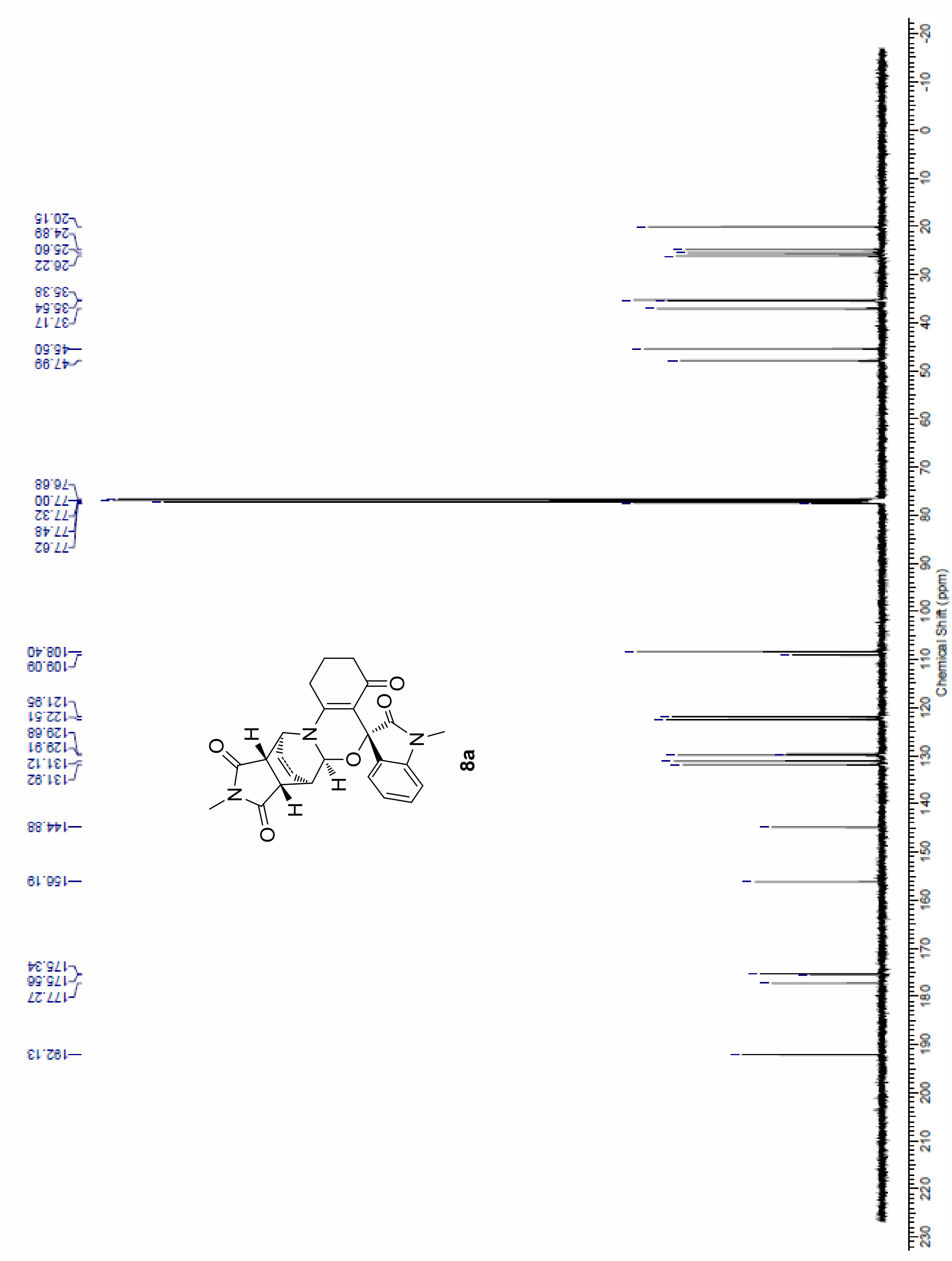
20.15
24.89
25.60
26.22
35.38
35.64
37.17
45.60
47.99
76.68
77.00
77.32
77.48
77.62
108.40
109.09
121.95
122.51
129.69
129.91
131.12
131.92
144.88
156.19
175.34
175.56
177.27
192.13
8a
Chemical Shift (ppm)

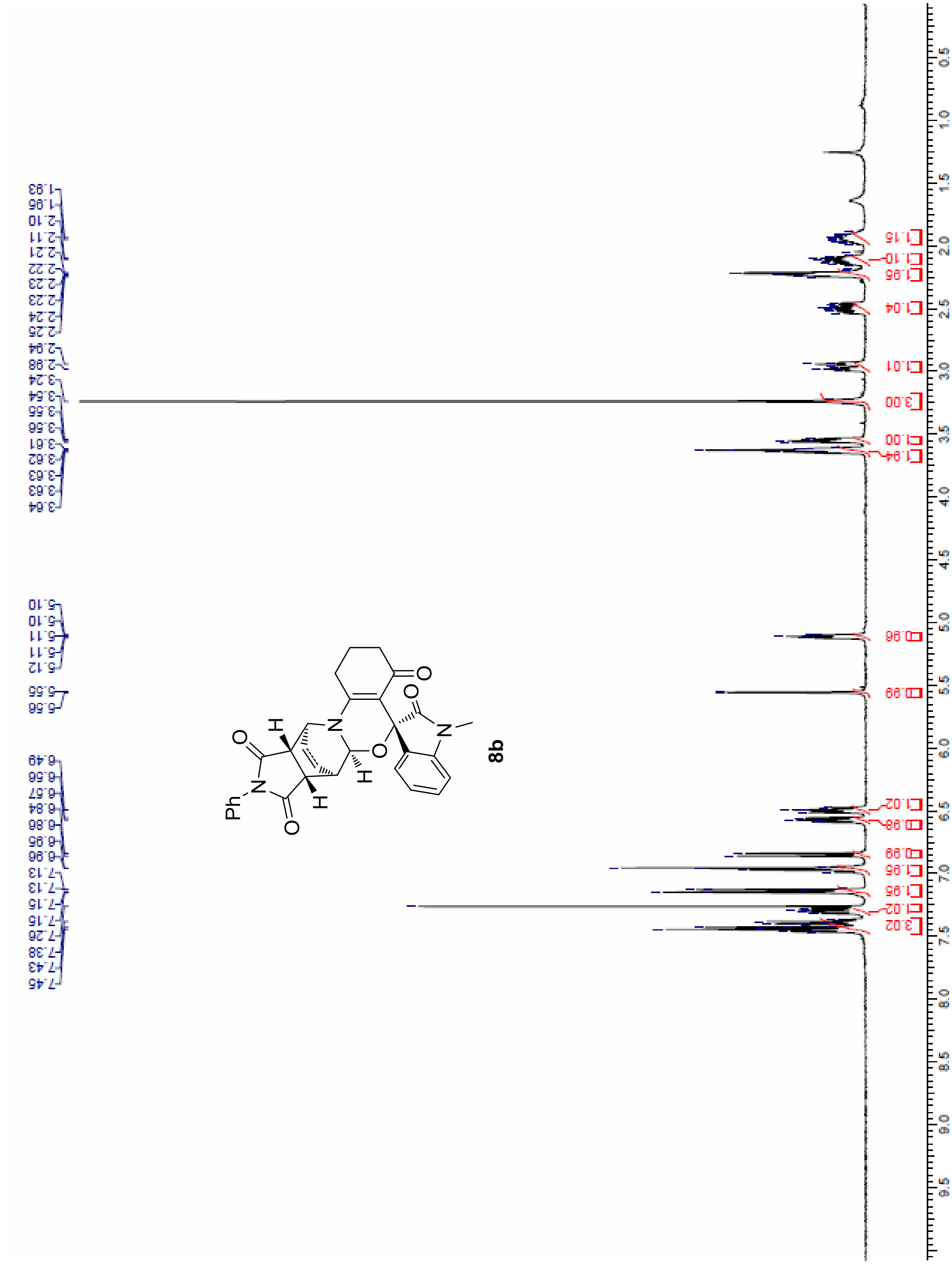
8b
Chemical Shift (ppm)

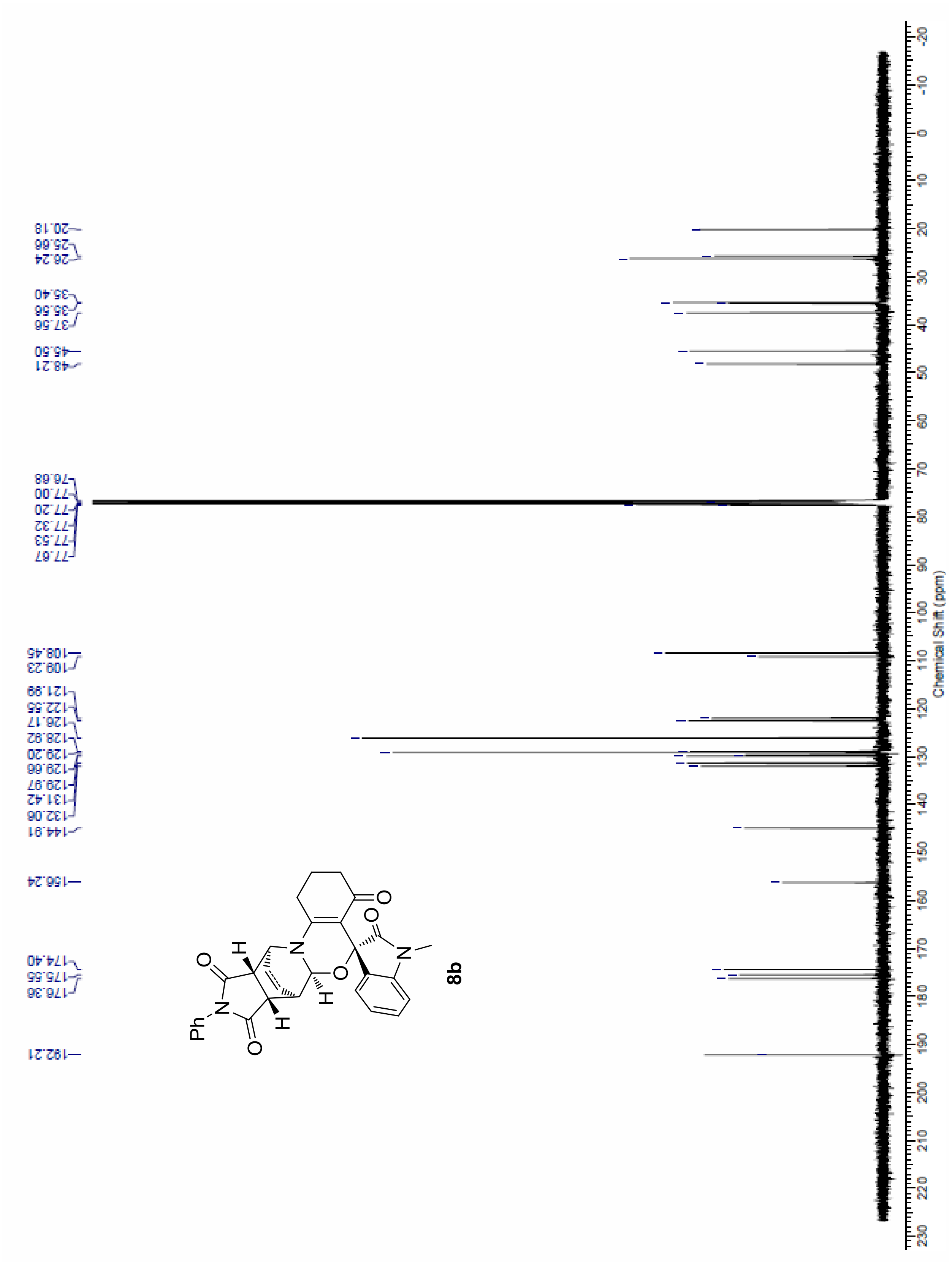
20.18
25.66
26.24
35.40
35.66
37.56
45.60
48.21
76.68
77.00
77.20
77.32
77.53
77.67
108.45
109.23
121.99
122.55
126.17
128.92
129.20
129.66
129.97
131.42
132.06
144.91
156.24
174.40
175.55
176.36
192.21
Ph
8b
Chemical Shift (ppm)

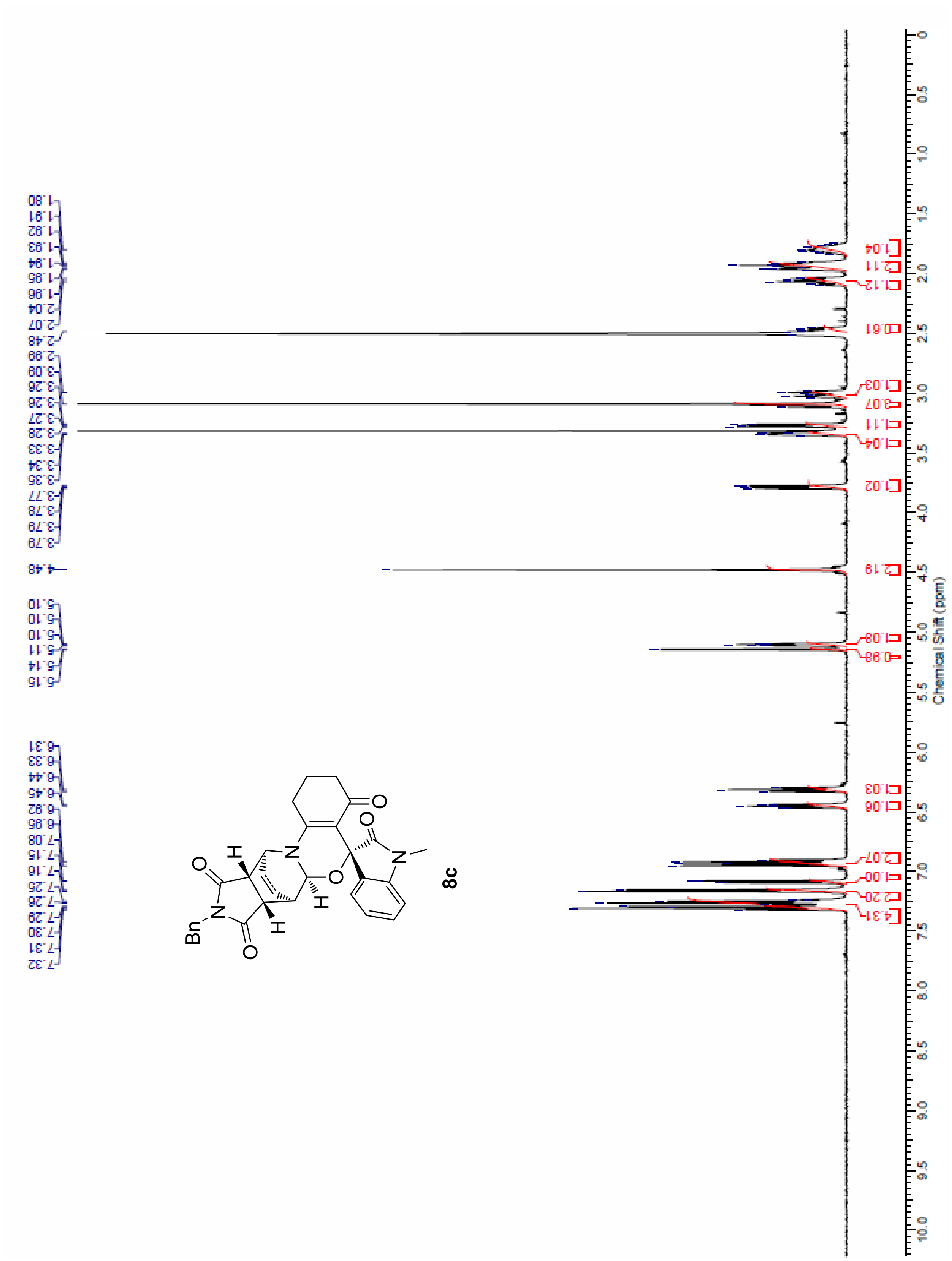
Chemical Shift (ppm)
8c

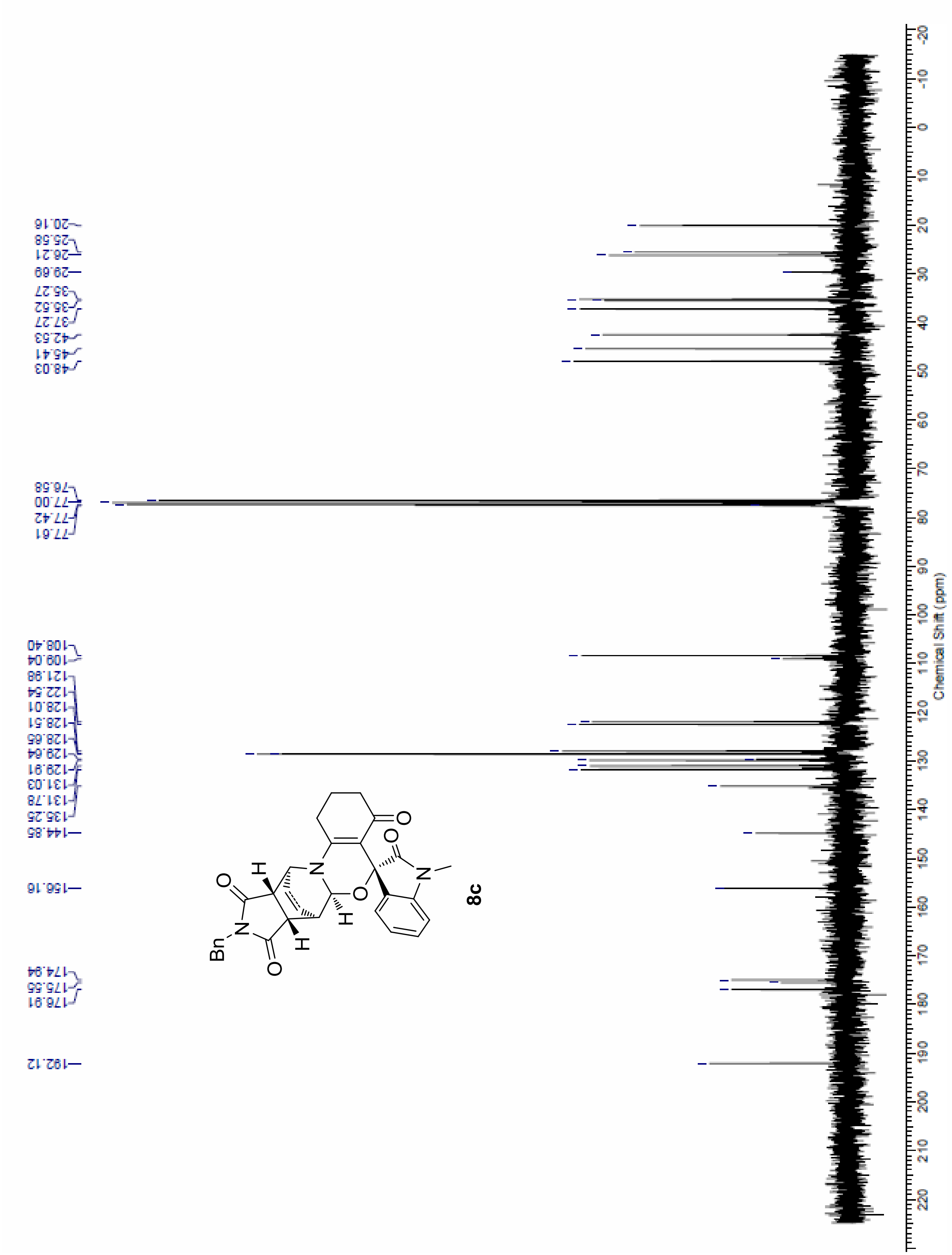
20.16
25.58
26.21
29.69
35.27
35.52
37.27
42.53
45.41
48.03
76.58
77.00
77.42
77.61
108.40
109.04
121.98
122.54
128.01
128.51
128.65
129.64
129.91
131.03
131.78
135.25
144.85
156.16
174.94
175.55
176.91
192.12
Chemical Shift (ppm)

8c

Determination of the conformation of compound 6h by gHMBC (500 MHz, $CDCl_3$)

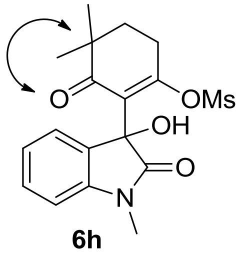

6h

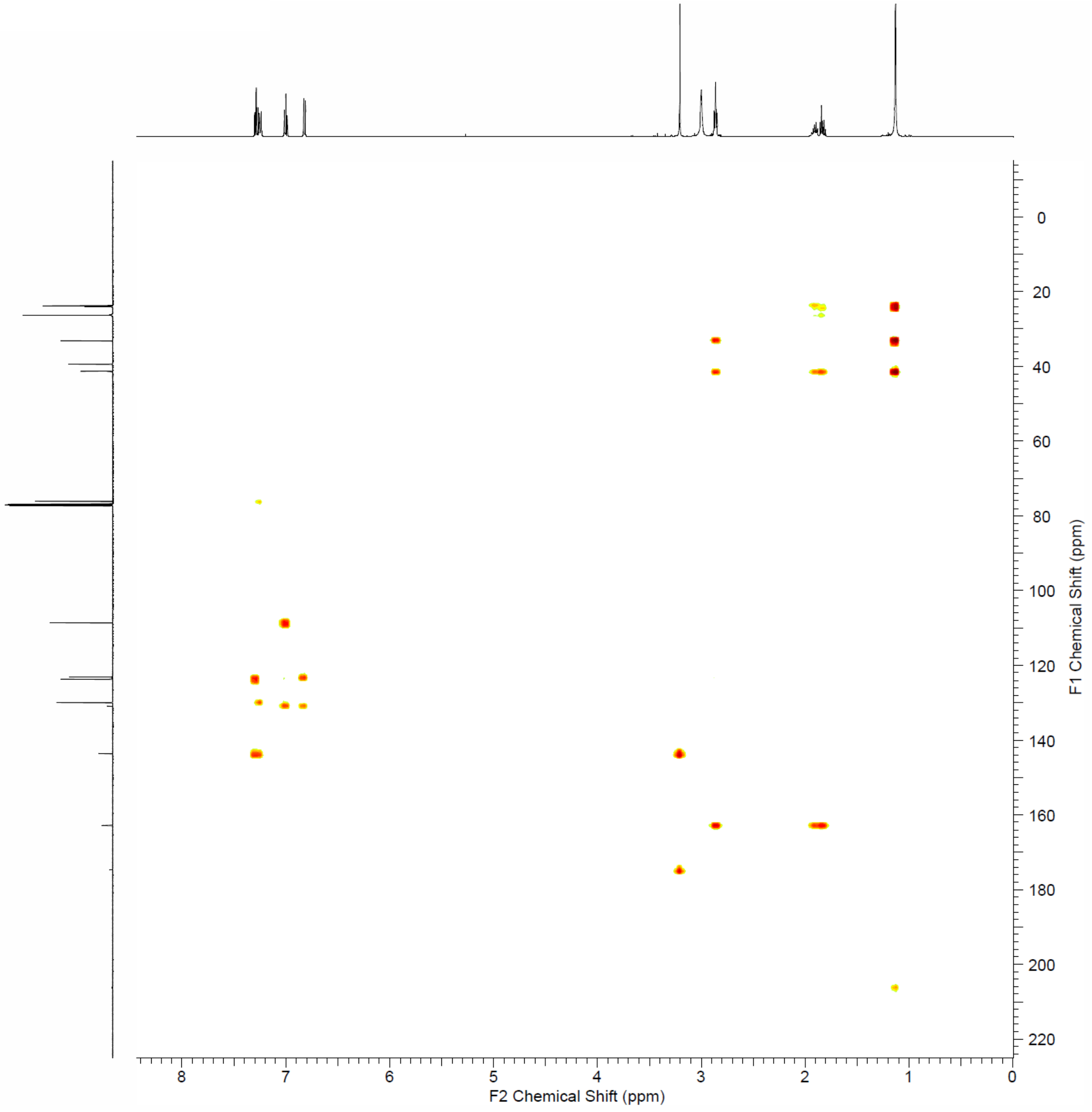

Key HMBC correlations of compound 6h.

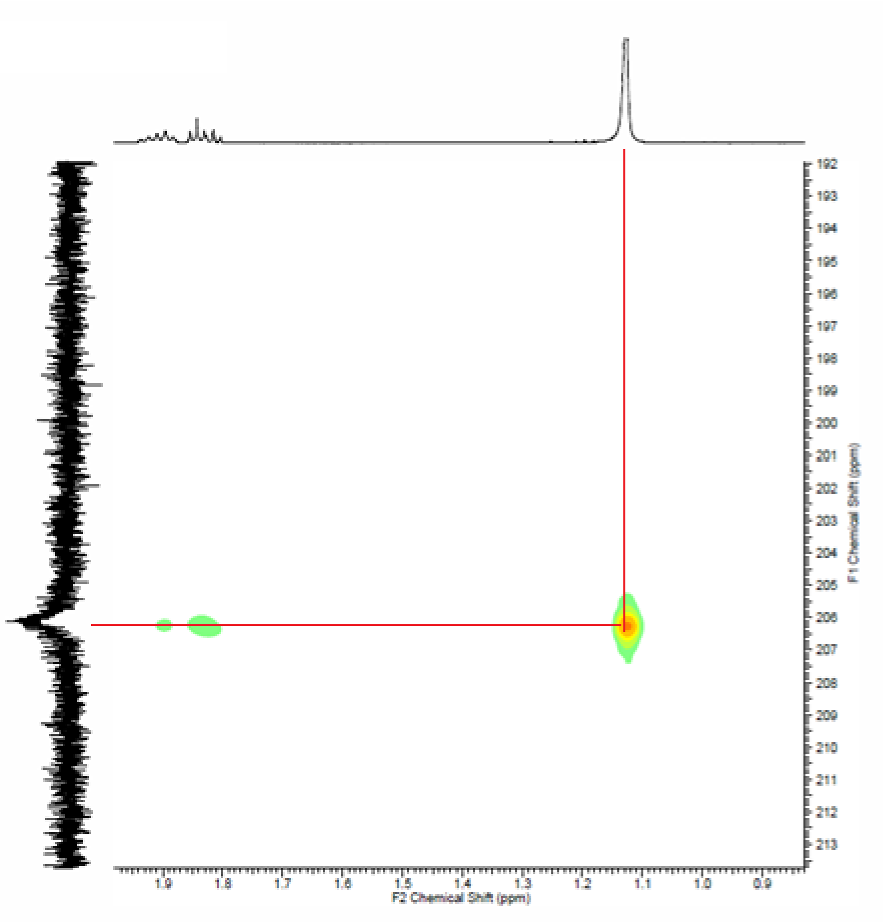

Supplement: File 1 — Full experimental details and analytical data and crystallographic information. [file Beilstein_J_Org_Chem-08-986-s001.pdf]
